# Using simulations to evaluate Mantel‐based methods for assessing landscape resistance to gene flow

**DOI:** 10.1002/ece3.2154

**Published:** 2016-05-21

**Authors:** Katherine A. Zeller, Tyler G. Creech, Katie L. Millette, Rachel S. Crowhurst, Robert A. Long, Helene H. Wagner, Niko Balkenhol, Erin L. Landguth

**Affiliations:** ^1^ Department of Environmental ConservationUniversity of MassachusettsAmherstMassachusetts01003; ^2^ Department of Fisheries and WildlifeOregon State UniversityCorvallisOregon97331; ^3^ Department of BiologyMcGill UniversityMontrealQuebecH3A 1B1Canada; ^4^ Field Conservation ProgramWoodland Park ZooSeattleWashington98103; ^5^ Department of Ecology and Evolutionary BiologyUniversity of TorontoMississaugaOntarioL5L 1C6Canada; ^6^ Department of Wildlife SciencesUniversity of GöttingenBüsgenweg 337077GöttingenGermany; ^7^ Division of Biological SciencesUniversity of MontanaMissoulaMontana59846

**Keywords:** CDPOP, landscape fragmentation, landscape genetics, landscape resistance, simulations

## Abstract

Mantel‐based tests have been the primary analytical methods for understanding how landscape features influence observed spatial genetic structure. Simulation studies examining Mantel‐based approaches have highlighted major challenges associated with the use of such tests and fueled debate on when the Mantel test is appropriate for landscape genetics studies. We aim to provide some clarity in this debate using spatially explicit, individual‐based, genetic simulations to examine the effects of the following on the performance of Mantel‐based methods: (1) landscape configuration, (2) spatial genetic nonequilibrium, (3) nonlinear relationships between genetic and cost distances, and (4) correlation among cost distances derived from competing resistance models. Under most conditions, Mantel‐based methods performed poorly. Causal modeling identified the true model only 22% of the time. Using relative support and simple Mantel *r* values boosted performance to approximately 50%. Across all methods, performance increased when landscapes were more fragmented, spatial genetic equilibrium was reached, and the relationship between cost distance and genetic distance was linearized. Performance depended on cost distance correlations among resistance models rather than cell‐wise resistance correlations. Given these results, we suggest that the use of Mantel tests with linearized relationships is appropriate for discriminating among resistance models that have cost distance correlations <0.85 with each other for causal modeling, or <0.95 for relative support or simple Mantel *r*. Because most alternative parameterizations of resistance for the same landscape variable will result in highly correlated cost distances, the use of Mantel test‐based methods to fine‐tune resistance values will often not be effective.

## Introduction

A primary goal of landscape genetics is to understand how landscape features influence observed spatial genetic structure (Manel et al. 2003). Mantel tests (Mantel 1967; Sokal 1979) and partial Mantel tests (Smouse et al. 1986) have been the predominant analytical methods for accomplishing this goal (Storfer et al. 2010). Mantel tests assess the correlation between two distance matrices—in landscape genetics, typically a matrix of interindividual or interpopulation genetic distances, and a matrix of geographic distances. Genetic distance can be quantified using individual‐based (e.g., proportion of shared alleles, *D*_*ps*_; Bowcock et al. 1994) or population‐based (e.g., local pairwise *F*_ST_; Nei 1973) measures. In all but the simplest models (i.e., isolation‐by‐distance or isolation‐by‐barrier), geographic distance is typically replaced by “effective distance” (Ferreras 2001) or “cost distance” (Adriaensen et al. 2003), which reflects both the geographic distance between individuals or populations and the degree to which the intervening landscape is hypothesized to impede gene flow and underlying dispersal movements (e.g., isolation‐by‐resistance; McRae 2006). Cost distance is calculated across a resistance surface wherein each cell in a geographic information system (GIS) raster is assigned a value based on a hypothesized species‐specific resistance to traversing the landscape feature the cell represents (Spear et al. 2010).

In a typical landscape genetics approach, cost distances among populations or individuals are calculated based on multiple, competing resistance hypotheses. These cost distances are then evaluated against empirical genetic distances among these same populations or individuals using Mantel tests or partial Mantel tests, sometimes within a causal modeling (CM) framework (Legendre and Legendre 2012; e.g., Cushman et al. 2006). Multiple regression with distance matrices (MRM; e.g., Wang 2013) extends Mantel methods to a multiple regression framework and is likely to suffer from similar limitations, with the additional problem that model selection with AIC and similar criteria is not valid for distance matrices (Wagner and Fortin 2015), and hence this method was not included in this study. Although there are other methods emerging for evaluating the relationship between genetic divergence and landscape features (Richardson et al. 2016), such as multimodel optimization with uncertainty estimates (Dudaniec et al. 2016), machine learning (Peterman et al. 2014; Ruiz‐Lopez et al. 2016), and ordination techniques (e.g., Kierepka and Latch 2014), Mantel‐based methods are still the mainstay of landscape genetic analyses.

Mantel‐based approaches for understanding landscape effects on genetic structure have been examined extensively using simulations. For example, many studies have tested the ability of Mantel tests to identify the true resistance model from a series of competing hypotheses (e.g., Balkenhol et al. 2009; Legendre and Fortin 2010; Jaquiéry et al. 2011; Cushman et al. 2013a). Other studies have explored the effect of landscape composition and configuration on the ability to discern the correct resistance model (e.g., Jaquiéry et al. 2011; Cushman et al. 2013b; Kierepka and Latch 2014), or the effect of sampling strategy and number of loci on the performance of these methods (Landguth et al. 2012a; Oyler‐McCance et al. 2012).

From this rich body of literature, we can identify at least four major challenges associated with the use of Mantel tests in landscape genetics. First, the performance of Mantel tests depends on the structure of the study landscape. Generally, less fragmented landscapes allow relatively unencumbered movement of individuals, resulting in only weak genetic structure and poor performance of Mantel‐based tests to correctly identify the true resistance model from a suite of options (Cushman et al. 2013a; Kierepka and Latch 2014). In contrast, landscapes with higher levels of fragmentation result in more pronounced genetic differentiation and an improved ability of Mantel tests to correctly identify the pattern–process relationship. Second, a substantial amount of time may be required before the genetic signature of a change in landscape structure is detectable with Mantel tests. Specifically, if the genetic signature of a particular landscape change has not yet equilibrated and become detectable, Mantel‐based tests may perform poorly compared to other approaches (Landguth et al. 2010). We, hereafter, refer to the point at which the landscape‐genetic relationship (i.e., the correlation among genetic and cost distances) has manifested as “spatial genetic equilibrium.” Third, Mantel tests are sensitive to violations of the underlying assumptions of linearity and independence (Diniz‐Filho et al. 2013; Legendre et al. 2015). Violating the independence assumption can lead to inflated type‐I error rates, while nonlinear relationships will reduce the power of Mantel tests to detect significant relationships between genetic and cost distances (i.e., increased type‐II error). Fourth, and perhaps most importantly, highly correlated cost distances among competing resistance hypotheses decrease the ability of Mantel‐based tests to identify the resistance surface that gave rise to the observed genetic structure (Balkenhol et al. 2009; Cushman et al. 2013b).

Despite substantial focus and research on the benefits and limitations of the Mantel test for landscape genetic analyses, knowledge gaps still exist. For example, many of the simulation studies that evaluated Mantel tests used only a binary habitat/nonhabitat classification. In reality, resistance surfaces are often much more complex and show gradients of cost values associated with continuous habitat variables (e.g., elevation, percentage forest cover). In landscapes with such gradients, the effects of composition and configuration on Mantel tests remain poorly understood. Furthermore, most studies use only a single landscape for their evaluations, despite the demonstrated relevance of composition and configuration for the performance of Mantel approaches. We are unaware of any current studies that assessed Mantel‐based tests using various gradient landscapes within a replicated study design.

Our goal is to address this current knowledge gap by assessing the four issues described above: landscape configuration, spatial genetic nonequilibrium, nonlinear relationships between genetic and cost distances, and correlation among competing resistance models. To accomplish this, we performed a spatially explicit, individual‐based, genetic simulation study using a variety of heterogeneous landscape configurations while representing resistance as a gradient. Based on previous studies, we hypothesized that the performance of Mantel‐based methods would increase (1) in more fragmented landscapes, (2) under spatial genetic equilibrium conditions, (3) when relationships between cost and genetic distances were linearized, and (4) when cost distances associated with competing resistance models were less correlated. We used this framework to test the ability of partial Mantel tests to discern the true resistance model using a suite of methods currently employed in the literature (hereafter, collectively referred to as “Mantel‐based methods”): (1) causal modeling (CM) with a significance level of *α *= 0.05, (2) CM with a stricter significance level of *α *= 0.005, (3) relative support from partial Mantel *r* values (i.e., relativized Mantel *r* values; Cushman et al. 2013b; Castillo et al. 2014), and (4) simple Mantel *r* values.

## Materials and Methods

### Simulating landscape variables and resistance models

**Figure 1:**
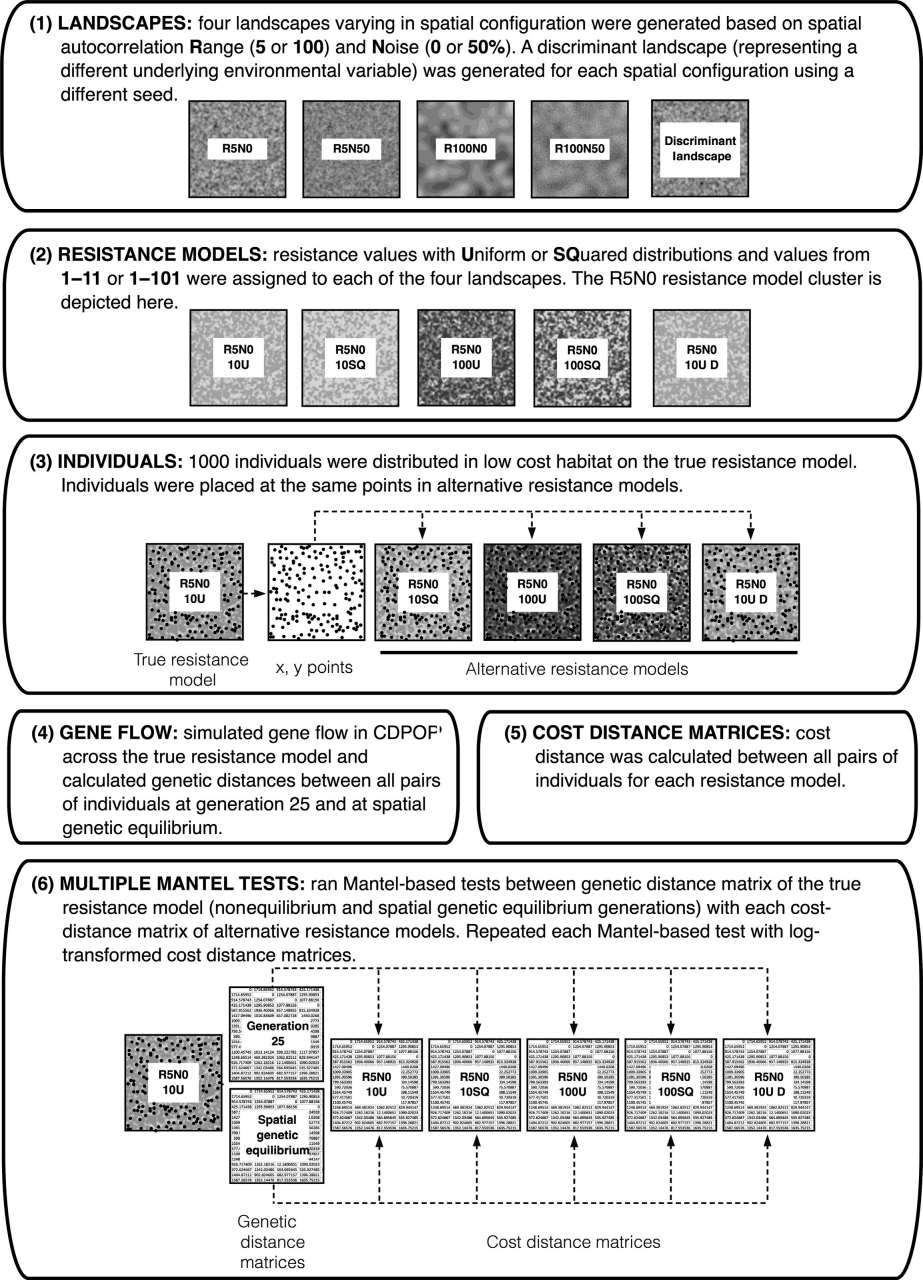
Summary of simulation methods used in this investigation. Each landscape was replicated five times and simulated using the same seed. A set of discriminant landscapes (using a different seed) was generated to represent a different underlying environmental variable. Gene flow was simulated across the true resistance model for 50 Monte Carlo replications, and Mantel‐based tests were evaluated using causal modeling (*α *= 0.05, 0.005), relative support, and simple Mantel *r*.

**Figure 2:**
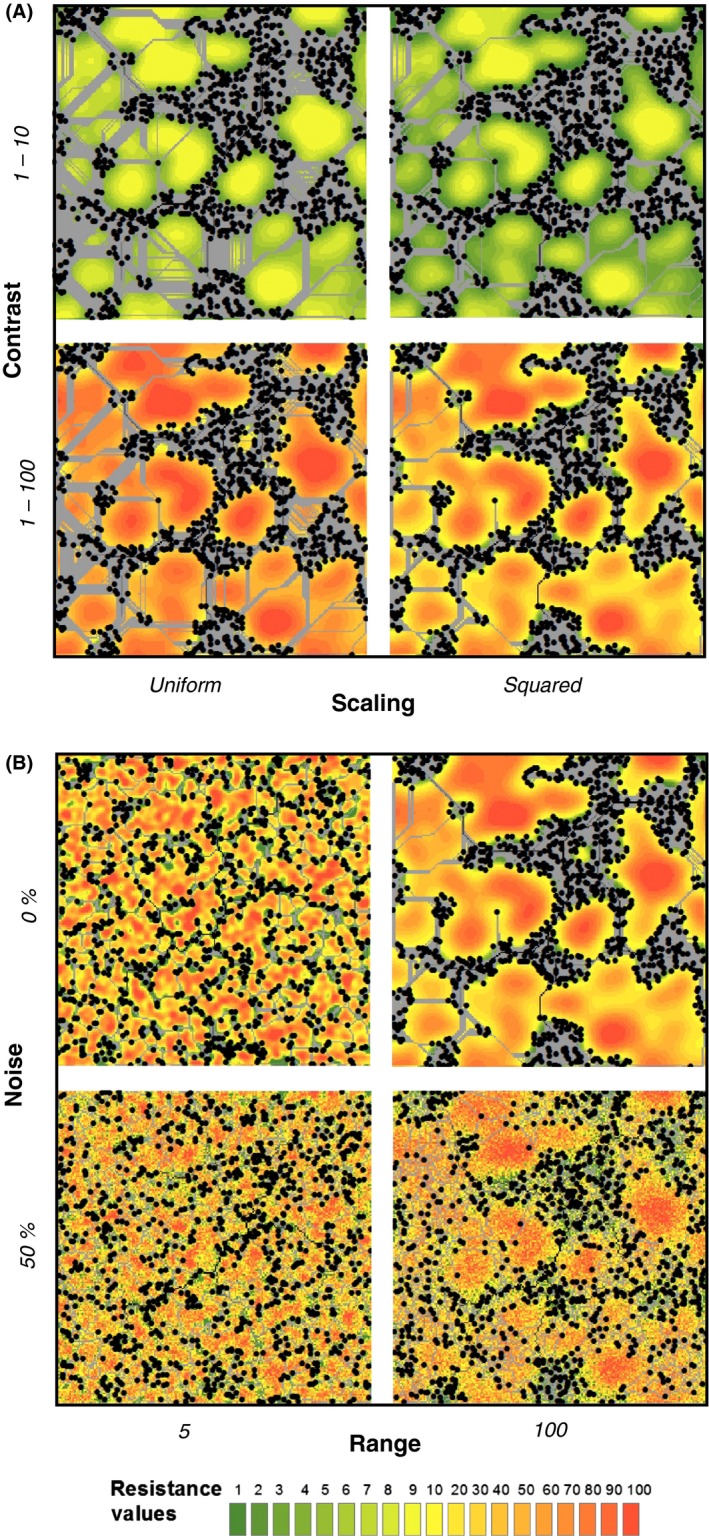
Examples of simulated resistance models. (A) Resistance models with equal levels of spatial autocorrelation scale and strength (autocorrelation range = 100, noise = 0%), but different resolution and scaling of resistance values. This represents a resistance model cluster for a R100N0 landscape. (B) Resistance surfaces with the same resolution and scaling of resistance values (1–101 with a squared distribution), but differing in the strength and scale of spatial autocorrelation. Black dots represent the locations of 1,000 simulated individuals. Gray lines represent least‐cost paths between all individuals.

A conceptual diagram for our study design is provided in Figure 1. We generated gradient landscapes 200 × 200 cells in size using R software (R Development Core Team 2011) and mimicked the effects of fragmentation by incorporating different scales of spatial autocorrelation and levels of noise into the underlying geospatial structure (Fig. 1.1). Starting with a random normal variable, we averaged cells within a moving window of 5 × 5 or 100 × 100 cells to produce landscape variables with short‐ or long‐range spatial autocorrelation, which we denoted as R5 and R100, respectively. In addition, we incorporated two levels of strength of spatial autocorrelation: strong autocorrelation without noise (N0) or weak autocorrelation with 50% noise (N50) by adding a random error. Noise was added by standardizing the landscape variable and adding a random variable with a standard deviation of one (see Appendix S14 for R code). This resulted in a factorial design with four types of landscape structure (Fig. 2B), which formed the basis for our four resistance model clusters (see below): R5N0, R5N50, R100N0, and R100N50.

We derived four alternative parameterizations of landscape resistance for each landscape variable to mimic the subtleties of fine‐tuning resistance values for a single landscape feature (e.g., elevation). The four resistance models represent four combinations of linear (U; values followed a uniform distribution) versus nonlinear functions (SQ; values followed a squared distribution) of the values of the landscape variables, and low (10; values between 1 and 11) versus high resolution (100; values between 1 and 101) of the resistance values (Fig. 1.2). These four parameterizations of each landscape comprise a single resistance model cluster. An example of a resistance model cluster for a R100N0 landscape is provided in Figure 2A. One example from all four clusters is provided in Appendix S1. For each resistance model cluster, we selected either the 10U or the 100SQ resistance model to represent our “truth”; these models were chosen because they represent opposite ends of the spectrum for maximum resistance value (10 vs. 100) and distribution (uniform vs. squared). To mimic the difficulty in discriminating between two different landscape features (e.g., elevation and percent forest cover), we used a second random seed to simulate a different, uncorrelated environmental variable for each resistance model cluster with the same level of fragmentation (Fig. 1.1). For this second landscape, we defined only one resistance model (hereafter referred to as the “discriminant” resistance model), using the same parameterization as our true resistance model (i.e., either 10U or 100SQ). One discriminant landscape was added to each resistance model cluster. Thus, a “cluster” was a set of five resistance models: the truth, three alternative resistance parameterizations of that same landscape variable, and the discriminant model representing a different landscape variable. We simulated eight resistance model cluster types: four with the 100SQ weighting scheme as the true model and four with the 10U weighting scheme as the true model. We created five replicates of each resistance model cluster, for a total of 8 resistance model cluster types × 5 landscape replicates × 50 replicate simulations.

For each of the true resistance models, we identified the 40th percentile of cell resistance values and considered all cells with resistance values less than this arbitrary cutoff to be suitable habitat. We used this 40th percentile to distribute individuals across our landscapes (see *“* Simulating gene flow *”* below). To quantify the amount of fragmentation in each landscape and facilitate the comparison of spatial structure across studies with different designs, we calculated Moran's *I*, correlation length, and patch cohesion. Moran's *I* (Moran 1950) measures the degree of spatial autocorrelation of a continuous spatial variable and was calculated directly from the resistance values for each model. Correlation length measures the distance that a randomly placed individual can move before exiting a patch, while patch cohesion measures patch continuity (Schumaker 1996). Correlation length and patch cohesion were calculated from the binary habitat maps using FRAGSTATS (McGarigal et al. 2012).

### Simulating gene flow

We randomly selected 1,000 habitat cells without replacement and placed one individual in each cell (Figs. 1.3, 2). Individual locations were consistent within landscape clusters, but varied among landscape clusters and replicates. We used CDPOP (Landguth and Cushman 2010) to simulate dispersal and gene flow for 1,000 individuals over 1,000 nonoverlapping generations across the true resistance model for each resistance model cluster (Fig. 1.4). We conducted 50 replicate simulations of 1,000 generations of gene flow for each true model. Sexual reproduction with an equal male–female sex ratio was specified, and reproduction followed a Poisson distribution with a mean litter size of four and an equal offspring sex ratio. Because this version of CDPOP would simulate immigrants from outside populations if the number of offspring was too low to recolonize all 1,000 habitat patches, we prevented unfilled habitat patches by specifying high reproductive levels. At the end of each generation, offspring that had not colonized an available habitat cell emigrated (i.e., were removed from the landscape). We randomly initialized genotypes for 30 loci with 30 alleles per locus and specified no mutation or selection. The maximum individual movement for mate‐seeking and dispersal was set to 15% of the maximum cost distance between individuals across the resistance surface, mimicking a species with limited dispersal. Sexes were assigned equal movement potential, and mate‐seeking and dispersal movements were assigned equal cost functions (based on cost distance matrices calculated for each resistance surface; details below). An inverse‐squared distance function was used to describe movement probabilities, such that most individuals travelled relatively short distances and few individuals travelled long distances.

Genotypes at generation 25 were used to generate genetic distance matrices of the proportion of shared alleles between all pairs of individuals; these data represented a population at spatial genetic disequilibrium. We then identified the generation after which spatial genetic equilibrium had been reached for each true resistance model by plotting Mantel *r* versus generation for each replicate and selecting the generation at which Mantel *r* began to plateau. We used the genotypes from this generation to construct a second genetic distance matrix for each true resistance model that represented a population at spatial genetic equilibrium. Our simulated populations reached spatial genetic equilibrium by generation 500 to 1,000 after initialization (see Appendix S2). Both equilibrium and nonequilibrium generations were used to compare each of the Mantel‐based methods.

### Calculating cost distances and evaluating relationships with genetic distance

Cost distance matrices for each resistance model were generated using UNICOR (Landguth et al. 2012b), which implements Dijkstra's (1959) algorithm to identify least‐cost pathways among individuals and calculate the cumulative path resistance (Fig. 1.5). Pearson's product–moment correlation coefficient (*r*) was calculated on a cell‐wise basis between the true resistance model and all competing resistance models for each cluster (cell‐wise correlations; Appendix S3). Pearson's correlation was also calculated between the cost distances from the true resistance model and the cost distances from competing resistance models for each cluster (cost distance correlations; Appendix S4).

We visually examined the relationship between genetic and cost distances using scatterplots (Appendix S5). In all cases, curvilinear relationships were observed. We attempted to linearize the relationship between genetic and cost distance by log‐transforming the cost distance values. The efficacy of the transformation was assessed by comparing the *R* ^2^ values from a linear regression model applied to the genetic data as a function of the transformed and untransformed cost distances (Appendix S5) and also by visual reexamination of the scatterplots posttransformation. If *R* ^2^ increased when using the log‐transformed values, we assumed the relationship became more linear. Both the transformed and untransformed cost distances were used with each of the Mantel‐based methods.

### Causal modeling with partial Mantel tests

We tested the ability of causal modeling (CM) with partial Mantel tests (Cushman et al. 2006; Cushman and Landguth 2010) to correctly identify the true resistance model from alternative resistance models for each landscape cluster. Our approach was similar to that used by Wasserman et al. (2010), in which all resistance models directly compete against one another in partial Mantel tests, in addition to competing against simple Euclidean distance models. This method tests all resistance models that meet the CM expectations in the Cushman et al. (2006) method against one another so that a single top model may be identified. R code is provided in Appendix S14.

For each resistance model cluster, we conducted partial Mantel tests of the general form *GD* ~ *CD1* | *CD2*, where the relationship between the simulated genetic distance matrix (*GD*) and cost distance matrix of a resistance model of interest (*CD1*) is assessed with an alternative model's cost distance matrix (*CD2*) partialled out (Fig. 1.6). All possible combinations of the five resistance models (four alternative parameterizations and one discriminant) were tested according to this general form. We also ran all possible tests with Euclidean distance partialled out to serve as a null model of isolation‐by‐distance; Euclidean distance was calculated from a raster in which all cells were assigned a value of one. Partial Mantel tests were run using genetic distances at generation 25 and at spatial genetic equilibrium, and with untransformed and log‐transformed cost distances, using the mantel() function in the “ecodist” (Goslee and Urban 2007) package in R.

Causal modeling supports a particular resistance model when: (1) all partial Mantel tests of the resistance model of interest (C1) are significant after cost distance matrices of alternative resistance models or Euclidean distances (C2) have been partialled out (five tests), and (2) all tests are nonsignificant when the resistance model of interest is partialled out for alternative model Mantel tests (five tests). To determine whether CM successfully identified the true resistance model, we counted the number of tests that was consistent with expectations (i.e., *P *< *α* for expected significant tests, or *P *> *α* for expected nonsignificant tests, where *α *= 0.05) for each of the five resistance models within each cluster replicate, assuming that each model could be the true model. For each cluster replicate, we calculated the success rate as the proportion of Monte Carlo (MC) replicates in which all ten tests were consistent with expectations for the true resistance model (i.e., the true model was correctly identified by CM).

### Alternative Mantel‐based approaches

We considered three alternative Mantel‐based methods. First, we used a more stringent criterion to assess statistical significance of Mantel test results by repeating the above procedure with *α *= 0.005. This was suggested by Oden and Sokal (1992), Diniz‐Filho et al. (2013), and Cushman et al. (2013b) to reduce type‐I error rates. Second, we used relative support (*RS*) based on Mantel *r* values (Cushman et al. 2013b; Castillo et al. 2014), calculated as the difference in Mantel *r* from the complementary set of partial Mantel tests:

RS=[GD∼CD1|CD2]−[GD∼CD2|CD1]

For each resistance model, we averaged *RS* values across all five tests with that resistance model as *CD1* in order to obtain a summary measure of the relative support for that resistance model compared to other resistance models within the cluster; we denote this quantity $\bar{RS}$. The model with the highest $\bar{RS}$ was considered the best‐supported, regardless of the magnitude of this value. We calculated the success rate as the proportion of MC replicates in which $\bar{RS}$ for the true resistance model was higher than for any of the alternative resistance models in the cluster. We repeated the entire process for each cluster.

Third, we used the Mantel *r* value from the simple Mantel test of the form *GD* ~ *CD1* to identify the best fitting resistance model, whereby resistance models with higher simple Mantel *r* were interpreted as having greater support. We calculated the success rate as the proportion of MC replicates in which simple Mantel *r* for the true resistance model was higher than for any of the alternative resistance models in the cluster.

### Performance of Mantel‐based tests when resistance models are based on different landscape variables

We also assessed the ability of Mantel‐based methods to distinguish between the true resistance model and the less correlated discriminant resistance model. To do this, we repeated the above analysis but compared the truth only to the discriminant surface, removing all other alternative surfaces from the set of candidate models. We calculated success rate for each method using the criteria described above (however, with a smaller number of partial mantel tests for CM due to the reduced number of resistance models being compared).

## Results

Each of the Mantel‐based methods performed poorly when all resistance models within a resistance model cluster were tested against each other, that is, when including alternative parameterizations of resistance values of the same landscape variable. Traditional CM, in which Mantel test results are evaluated using *P*‐values and a significance level of *α *= 0.05, identified the true resistance model only 22% of the time (Table 1; mean across approaches). There was a negligible improvement in method performance to 23% success when *α *= 0.005 was used. However, there was considerable variation in performance depending on whether the cost distances were linearized and if the equilibrium generation was used. The best performance of traditional CM (41% success rate) occurred when *α *= 0.005, cost distances were linearized, and a spatial equilibrium generation was used (Table 1). Using $\bar{RS}$ rather than significance‐based CM to evaluate resistance models boosted CM performance to 47% (Table 1; mean across approaches). Similar results were obtained using simple Mantel *r* values, while linearizing cost distances increased performance of both $\bar{RS}$ and simple Mantel *r* methods. There was also a slight performance increase when an equilibrium generation was used, though $\bar{RS}$ and simple Mantel *r* seemed fairly robust to whether genetic equilibrium had been reached or not (Table 1, Log = Y/Eq = N and Log = Y/Eq = Y columns).

**Table 1:** Success rate of Mantel‐based methods to identify the true resistance model when compared to all the competing resistance models in a landscape cluster (including the discriminant surface). Success rate is the proportion of all 50 MC replicates in which the true resistance model outperformed all other resistance models in a cluster. Results are pooled across truth 10U and truth 100SQ. *Log* refers to whether the cost distances were log‐transformed to better linearize their relationship with genetic distance. *Eq* indicates whether the genetic data used were from a generation that had reached genetic equilibrium or whether a prior generation was used. Numbers in table are means with standard deviations in parentheses

	Log = N Eq = N	Log = N Eq = Y	Log = Y Eq = N	Log = Y Eq = Y	Mean across approaches
Causal modeling (*α *= 0.05)	0.009 (0.044)	0.313 (0.426)	0.153 (0.310)	0.388 (0.450)	0.216 (0.374)
Causal modeling (*α *= 0.005)	0.013 (0.052)	0.326 (0.427)	0.173 (0.323)	0.414 (0.448)	0.231 (0.379)
Relative support (RS)	0.430 (0.425)	0.397 (0.424)	0.511 (0.405)	0.547 (0.423)	0.471 (0.420)
Simple Mantel *r*	0.462 (0.414)	0.468 (0.455)	0.522 (0.419)	0.554 (0.437)	0.501 (0.429)
Mean across methods	0.228 (0.367)	0.376 (0.434)	0.339 (0.405)	0.475 (0.442)	0.354 (0.421)

**Figure 3:**
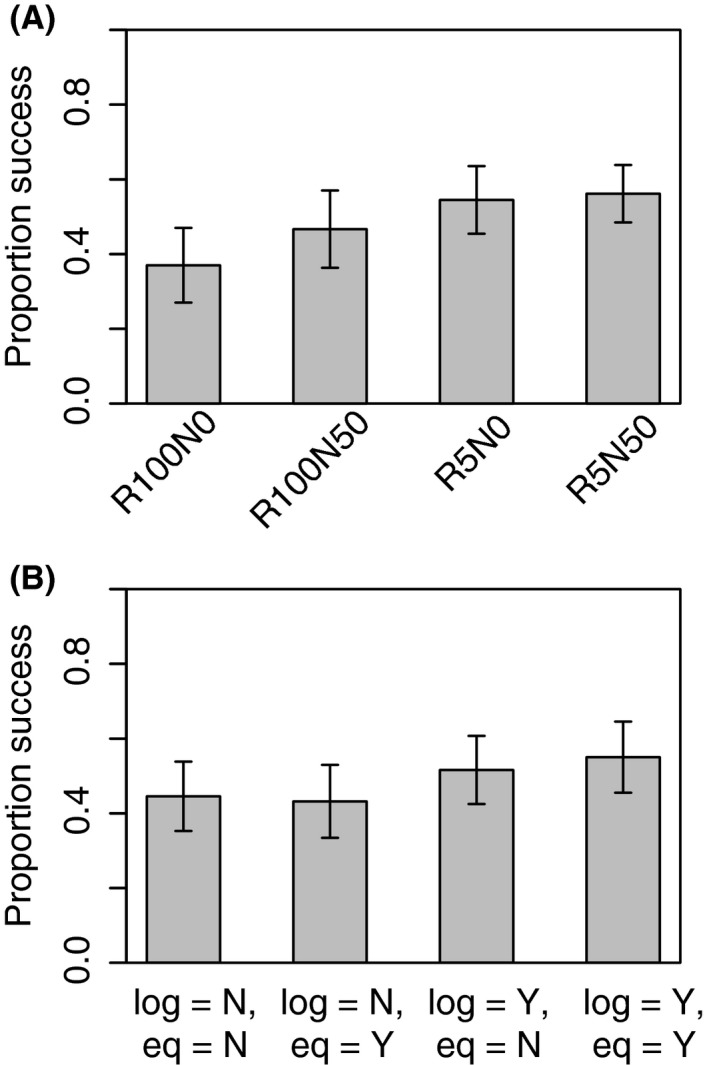
Model performance as judged by the proportion of MC replicates in which the true resistance model outperformed all other resistance models. Proportion success is averaged across the $\bar{RS}$ and simple Mantel *r* methods. Error bars represent the 95% confidence intervals. Causal modeling results have been omitted. Truth 10U and 100SQ results are pooled. (A) Model performance by landscape cluster. (B) Model performance by all possible combinations of linearity and spatial genetic equilibrium. *Log* refers to whether the cost distances were log‐transformed to linearize their relationship with genetic distance. *Eq* indicates whether the genetic data used were from a generation that had reached spatial genetic equilibrium or whether a prior generation was used.

Given the poor performance of the traditional CM methods, we focus our landscape structure results on the $\bar{RS}$ and simple Mantel *r* results. We observed considerable variation in the performance of the $\bar{RS}$ and simple Mantel *r* methods among landscape clusters. Landscape variables with fine‐scale spatial autocorrelation (R5) outperformed those with large‐scale spatial autocorrelation (R100; Fig. 3; Appendix S8). For instance, across clusters with no noise, the correct resistance model was selected 54% of the time for fine‐scale autocorrelation (R5N0), but only 37% of the time with large‐scale autocorrelation (R100N0). Again, performance increased when the cost distances were linearized and when an equilibrium generation was used (Fig. 3; Appendix S8). The level of noise had a smaller but noticeable effect. Landscape variables with noise of 50% tended to outperform those with no noise (e.g., 47% vs. 37% for the landscape cluster with large‐scale spatial autocorrelation, R100; Fig. 3; Appendix S8). Landscape variables with a greater range of autocorrelation exhibited less fragmentation: Moran's *I* values were higher, and patch cohesion and correlation length metrics were larger for the R100 landscape variables compared with the R5 landscape variables (Appendices S6 and S7). Landscape variables with noise exhibited more fragmentation with lower Moran's I values and small patches becoming more disjointed than in landscapes without noise (Appendices S6 and S7).

When the true resistance model was tested only against the discriminant resistance model for each cluster, mimicking the problem of discriminating between resistance due to two different landscape variables (e.g., elevation vs. percent forest cover), performance of the two CM methods increased to 57% and 61%, respectively, while the performance of $\bar{RS}$ and simple Mantel *r* increased to above 98% (Table 2). The discriminant resistance models had much lower cell‐wise correlations with the true model (≤0.11) than other resistance models in the same cluster (>0.96; Appendices S3 and S9). However, cost distance correlations with the true model were much more variable than cell‐wise correlations (Appendix S9), ranging approximately from 0.45 to 0.98 for discriminant resistance models and from 0.85 to 0.99 for alternative parameterizations of resistance for the same landscape factor. We found that performance of Mantel‐based methods varied with cost distance correlations (Appendix S10). Despite relatively high cost distance correlations, $\bar{RS}$ and simple Mantel *r* performed very well at selecting the true resistance model over the discriminant model, especially when the relationship between cost and genetic distance was linearized. The success rate of the CM methods was more sensitive to these cost distance correlations, with the success rate dropping for cost distance correlations >0.85, even when the relationship between cost and genetic distance was linearized (Appendix S10A).

**Table 2:** Success rate of Mantel‐based methods to identify the true resistance model over the discriminant resistance model (i.e., proportion of 50 MC replicates in which true model outperformed discriminant model) when only those two models are included as competing hypotheses. Truth 10U and truth 100SQ results are pooled. *Log* refers to whether the cost distances were log‐transformed to linearize their relationship with genetic distance. *Eq* indicates whether the genetic data used were from a generation that had reached genetic equilibrium or whether a prior generation was used. Numbers in table are means with standard deviations in parentheses

	Log = N Eq = N	Log = N Eq = Y	Log = Y Eq = N	Log = Y Eq = Y	Mean across approaches
Causal modeling (*α* = 0.05)	0.156 (0.300)	0.783 (0.317)	0.629 (0.403)	0.739 (0.321)	0.577 (0.418)
Causal modeling (*α *= 0.005)	0.186 (0.323)	0.820 (0.289)	0.665 (0.394)	0.784 (0.293)	0.613 (0.412)
Relative support (RS)	0.999 (0.006)	0.976 (0.094)	1.000 (0.000)	0.981 (0.084)	0.989 (0.063)
Simple Mantel *r*	0.999 (0.006)	0.976 (0.094)	1.000 (0.000)	0.981 (0.084)	0.989 (0.063)
Mean across methods	0.585 (0.470)	0.889 (0.239)	0.823 (0.331)	0.871 (0.249)	0.792 (0.356)

## Discussion

We simulated multiple continuous landscape variables to examine the performance of four Mantel‐based methods for assessing landscape resistance. While our results are generally consistent with findings from previous simulations studies, they provide several novel insights that further clarify the utility of the various Mantel‐based methods for landscape genetics. We note that our simulated landscapes represented extremes of spatial autocorrelation, and the effects of landscape characteristics on method performance should be further studied. Furthermore, the specific results may depend to some degree on the details of our study design and analysis (e.g., we did not attempt to linearize the relationship between genetic distance and Euclidean distance). We had a larger sample size and thus higher power than most real‐world studies. To determine whether our results were applicable to smaller sample sizes, we repeated our analyses with a random sample of 100 individuals and found that our conclusions remain unchanged (Appendices S12 and S13). This corroborates the findings of Landguth et al. (2012a) who found that the results of Mantel tests were relatively unaffected by sample size when compared with the number of loci and number of alleles. Oyler‐McCance et al. (2012) found sample size and sampling strategy to affect performance of Mantel‐based tests, particularly when populations are not at genetic equilibrium. Therefore, to fully understand how sampling would affect our results, further research is needed that examines sample size, sampling strategy, and the number of loci and alleles.

### Comparison of Mantel‐based methods

Each of the Mantel‐based methods performed poorly when all resistance models within a cluster were tested against each other, underscoring the difficulties in discriminating between alternative parameterizations of the same underlying landscape variable. However, substantial differences became evident when contrasting resistance models derived from different landscape variables (i.e., the true resistance model vs. the discriminant resistance model). CM methods did not perform as well as $\bar{RS}$ and simple Mantel *r*. This was especially true when alternative parameterizations of resistance for the same landscape variable were included. The sheer number of tests that need to be passed for CM to select a resistance model made it difficult for CM to identify the true resistance model, particularly when competing models were highly correlated. In contrast, $\bar{RS}$ and simple Mantel *r* performed very well at discriminating among resistance models derived from different underlying landscape variables and were >98% successful when the relationship was linearized. This suggests that these Mantel‐based methods may be very effective for discriminating among different landscape variables and that their use is justified in this narrow application.

### Challenge 1: Effects of landscape structure

Previous studies have shown that the composition and configuration of resistant features across landscapes of interest can affect Mantel test performance. Cushman et al. (2013a) and Kierepka and Latch (2014) found that landscapes with lower levels of fragmentation allowed relatively unencumbered movement of individuals, resulting in only weak genetic structure and poor performance of Mantel‐based tests to correctly identify the true resistance model from a suite of options. Landscapes with higher levels of fragmentation resulted in more pronounced genetic differentiation and the ability to correctly identify the pattern–process relationship. This finding was also confirmed by an empirical study that was unable to associate spatial genetic structure in bobcats (*Lynx rufus*) with habitat connectivity in landscapes with low levels of fragmentation (Reding et al. 2013). However, Jaquiéry et al. (2011) found that performance of Mantel‐based methods was poorer with increasing fragmentation. This seemingly conflicting result is actually quite plausible under very high levels of fragmentation, when movement and gene flow across the landscape become so severely restricted that a significant relationship between gene flow and landscape resistance is no longer present. Importantly, all three studies found an effect of landscape composition, whereby higher degrees of contrast in resistance values led to better performance of Mantel‐based tests. These findings were largely corroborated by our simulations based on gradient landscapes with varying degrees of spatial autocorrelation and random noise.

In landscapes with more pronounced levels of fragmentation (i.e., finer‐scale spatial autocorrelation and greater noise, resulting in lower aggregation), performance of Mantel‐based methods increased. We also found that Mantel‐based methods performed better when the true resistance model had a higher degree of contrast in resistance values (100SQ vs. 10U; Appendix S11). This means that Mantel‐based methods will work best in highly fragmented landscapes that show strongly contrasting resistance values, regardless of whether a binary or continuous landscape representation is used.

### Challenge 2: Effect of time lags

As in previous studies, we expected that the performance of Mantel‐based methods in our gradient landscapes would be higher when the landscape effect had already manifested itself in the spatial genetic structure of the population. However, the use of a spatial genetic equilibrium generation only mildly improved the performance of traditional CM methods, while $\bar{RS}$ and simple Mantel *r* methods were robust to whether spatial genetic equilibrium was reached or not. In comparison with the importance of linearity and the testing of different landscape variables (in lieu of alternative parameterizations of resistance for the same landscape variable), spatial genetic equilibrium appears to play a more minor role in the accuracy of Mantel‐based methods. This is somewhat comforting, as many environments are changing rapidly and most empirical studies will be unable to confirm spatial genetic equilibrium of their target populations without repeated sampling over long timescales and extensive knowledge of a species' history in an area. Previous research has demonstrated that Mantel tests can detect effects of barriers in otherwise homogeneous landscapes in as few as 15 generations (Landguth et al. 2010); to our knowledge, our study is the first to examine this issue of time lag using a gradient of resistance values.

### Challenge 3: Effects of nonlinearity

In support of previous studies, our results provide strong evidence for the importance of checking the assumptions of linearity when using the Mantel test (see also Diniz‐Filho et al. 2013 and Legendre et al. 2015). While there are many reasons why the linearity assumption may be violated (e.g., genetic or demographic stochasticity; Diniz‐Filho et al. 2013; Graves et al. 2013), most landscape genetic studies do not assess this relationship before applying Mantel tests (but see Shirk et al. 2010 and Graves et al. 2013). This is actually quite surprising, considering that evaluating linearity is a relatively straightforward task to carry out prior to the final analysis. For example, we assessed the shape of our cost/genetic distance relationships visually, and by determining whether the *R* ^2^ value increased when we transformed the cost distance values. A more flexible approach would be to use a Box‐Cox analysis (Box and Cox 1964) to identify the exponent value (*λ*) that would transform the data in a way that best meets the assumptions of linearity and normality. The Box‐Cox analysis is useful in that it not only indicates whether data meet assumptions, but also identifies the appropriate transformation (if needed). The power of Mantel‐based methods may be further improved by reducing the full distance matrix to a subset of pairs (Wagner and Fortin 2013), either using a spatial graph model (Dale and Fortin 2010) or conditional genetic distance (Dyer et al. 2010; Garroway et al. 2011).

### Challenge 4: Effects of correlated resistance models and cost distances

Poor performance across methods was likely due to the inability of the Mantel‐based tests to accurately discern the truth from a suite of very highly correlated resistance models and their associated cost distances. In our simulations, all alternative resistance models in a cluster (i.e., alternative resistance parameterizations based on the same underlying landscape variable as the true model) had cell‐wise correlations ≥0.97 and cost distance correlations ≥0.93 with the true model. Changing only the scaling (U vs. SQ) and not the resolution (10 vs. 100) reduced the cell‐wise correlation, but had a relatively small effect on the cost distance correlations, whereas changing only the resolution of resistance values (10 vs. 100) reduced cost distance correlations but not cell‐wise correlations. Changing both scaling and resolution led to the largest reductions in cost distance correlations, but correlations among alternative resistance models and the true model still remained high (>0.85). As predicted, the discriminant resistance models had very low cell‐wise correlations with the true model (<0.11). However, even very low cell‐wise correlations could result in very high cost distance correlations as evidenced by the R510U discriminant models (correlations >0.9), and it is the latter that determine performance of Mantel‐based methods (see Appendix S10). In general, however, the cost distance correlations between the discriminant model and the true model were often lower than correlations among the true model and alternative resistance models in the same cluster. Our results agreed with Cushman et al. (2013b), in that we found a drop in the performance of CM methods when linearized cost distance correlations increased above 0.85, which is higher than the commonly used 0.7 collinearity threshold for regression analysis that is not based on distance matrices (Dormann et al. 2013). $\bar{RS}$ and simple Mantel *r* had higher performance than CM, indicating that they were better able to select the true model even when cost distance correlations were high.

Our results suggest that Mantel‐based methods should not be used for fine‐tuning relative weights of resistance values when competing resistance models exhibit high‐cost distance correlations (>0.85). Even lower cost distance correlations were sometimes problematic for CM, particularly when spatial genetic equilibrium had not been reached (Appendix S10B). Given that most alternative parameterizations of resistance for the same landscape variables will result in very high‐cost distance correlations, the use of Mantel‐based methods to fine‐tune resistance values will not be effective. Mantel‐based methods seem better suited to discriminate among resistance models with different underlying landscape variables, and $\bar{RS}$ and simple Mantel *r* outperform CM in this regard.

## Conclusions and Recommendations

Our analysis is the first to examine all four challenges mentioned above using spatially explicit simulations with continuous landscape variables representing different spatial compositions and configurations. We recognize that Mantel‐based tests are often used to compare resistance models based on different parameterizations of a single landscape variable and that comparing resistance models that are based on independent landscape variables is often not possible or not helpful for answering a question of interest. We believe these methods still have applicability for landscape genetics, but under a much more restricted set of circumstances than they have been applied in the past. Due to the limitations of the Mantel test found here and in other studies (e.g., Graves et al. 2013; Guillot and Rousset 2013), the field of landscape genetics is currently at a crossroads. There are several alternative methods to the Mantel test that have been proposed, but none are able to simultaneously address spatial autocorrelation, the testing of alternative resistance models, and the ability to perform hypothesis testing (Richardson et al. 2016). Until a silver bullet is found, the Mantel test will likely continue to be used. In the absence of other methods to discern between resistance models using the same variable, Mantel‐based tests would provide the best outcome if (1) the research was conducted in more fragmented (less aggregated) landscapes, (2) the target population had reached spatial genetic equilibrium, (3) the cost/genetic distance relationship was linearized, (4) the resistance parameterizations were very different from each other, resulting in lower correlations among cost distances (<0.85 for causal modeling, <0.95 for $\bar{RS}$ or simple Mantel *r*), and (5) either $\bar{RS}$ or simple Mantel *r* was used as the metric of choice. Unfortunately, options (1) and (2) above are rarely under the control of the researcher, leaving one to rely on options (3), (4), and (5).

## Data Accessibility

Data is available on DRYAD.

R code is available in our Supporting Information (Appendix S14).

CDPOP software and user manual are available on: (http://cel.dbs.umt.edu/cms/CDPOP).

UNICOR software and user manual are available on: (http://cel.dbs.umt.edu/unicor).

## Conflict of Interest

None declared.

## Supporting information


**Appendix S1.** Example of the four landscape clusters.
**Appendix S2.** Generation time for populations to reach spatial genetic equilibrium across our true resistance model for Replicate 1.
**Appendix S3.** Simulated replications of resistance models organized by cluster, showing cell‐wise correlations of true versus competing resistance models (Pearson's *r*).
**Appendix S4.** Simulated replications of resistance models organized by cluster, showing cost‐distance correlations of true versus competing resistance models (Pearson's *r*).
**Appendix S5.** Relationship between cost distance and genetic distance for Replicate 1 of the R5N0_100SQ landscape; (A) untransformed, and (B) with cost distances natural log‐transformed.
**Appendix S6.** Simulated replications of resistance models organized by cluster, showing Moran's *I*.
**Appendix S7.** Correlation length (A) and patch cohesion metrics (B) for the simulated resistance models.
**Appendix S8.** Model performance (proportion of runs in which true resistance model outperformed all other resistance models in a cluster) by cluster.
**Appendix S9.** Scatterplot showing the cell‐wise correlation between each resistance model in a cluster and the true resistance model (*x*‐axis), plotted against the cost‐distance correlation between each resistance model in a cluster and the true resistance model (*y*‐axis).
**Appendix S10.** (A) Cost‐distance correlations plotted against model performance for the discriminant resistance models and for one of the alternative resistance models for each cluster. (B) Cost‐distance correlations plotted against model performance for the discriminant resistance models (circles) and for one of the alternative resistance models for each cluster (triangles).
**Appendix S11.** Model performance as judged by the proportion of MC replicates in which the true resistance model outperformed all other resistance models in a cluster.
**Appendix S12.** Success rate of Mantel‐based methods when comparing amongst all resistance models within a cluster, including those based on different parameterizations of the same landscape variable (i.e., proportion of MC replicates in which the true resistance model outperformed all other resistance models), and using a random subsample of 100 individuals.
**Appendix S13.** Success rate of Mantel‐based methods to select the true resistance model over the discriminant resistance model (i.e., proportion of MC replicates in which true model outperformed discriminant model) when only those two models are included as competing hypotheses, and using a random subsample of 100 individuals.

**Appendix S14.** R and Python code for Mantel‐based test simulation analysis.
