# Supplementary material for: Using simulations to evaluate Mantel‐based methods for assessing landscape resistance to gene flow
Source: Ecol Evol. 2016 May 21;6(12):4115–28. doi: 10.1002/ece3.2154 (PMC4879002; doi:10.1002/ece3.2154)
Supplement: Supplementary file 1 — Appendix S1. Example of the four landscape clusters. Appendix S2. Generation time for populations to reach spatial genetic equilibrium across our true resistance model for Replicate 1. Appendix S3. Simulated replications of resistance models organized by cluster, showing cell‐wise correlations of true versus competing resistance models (Pearson's r). Appendix S4. Simulated replications of resistance models organized by cluster, showing cost‐distance correlations of true versus competing resistance models (Pearson's r). Appendix S5. Relationship between cost distance and genetic distance for Replicate 1 of the R5N0_100SQ landscape; (A) untransformed, and (B) with cost distances natural log‐transformed. Appendix S6. Simulated replications of resistance models organized by cluster, showing Moran's I. Appendix S7. Correlation length (A) and patch cohesion metrics (B) for the simulated resistance models. Appendix S8. Model performance (proportion of runs in which true resistance model outperformed all other resistance models in a cluster) by cluster. Appendix S9. Scatterplot showing the cell‐wise correlation between each resistance model in a cluster and the true resistance model (x‐axis), plotted against the cost‐distance correlation between each resistance model in a cluster and the true resistance model (y‐axis). Appendix S10. (A) Cost‐distance correlations plotted against model performance for the discriminant resistance models and for one of the alternative resistance models for each cluster. (B) Cost‐distance correlations plotted against model performance for the discriminant resistance models (circles) and for one of the alternative resistance models for each cluster (triangles). Appendix S11. Model performance as judged by the proportion of MC replicates in which the true resistance model outperformed all other resistance models in a cluster. Appendix S12. Success rate of Mantel‐based methods when comparing amongst all resistance models within a cluster, includin [file ECE3-6-4115-s001.docx]

Appendix S1. Example of the four landscape clusters. The following figures display the landscape clusters for replicate 1 of the 10U truths. Each 200 x 200 landscape was simulated with one of two autcorrelation ranges (5 or 100) and one of two levels of noise (0% or 50%). The base resistance surface for each landscape cluster was created by ordering all the original values (0 - 1) and reclassifying them to follow a uniform distribution (U) ranging from 1 – 11 (referred to as our 10U landscapes). We created three additional resistance surfaces: (1) a uniform distribution and a range of resistances from 1 – 101, (2) a squared distribution (SQ) and a range of resistances from 1 – 11, and (3) a squared distribution and a range of resistances from 1 – 101. Black dots indicate the locations of 1,000 simulated individuals. Grey lines represent the least cost paths between all individuals.


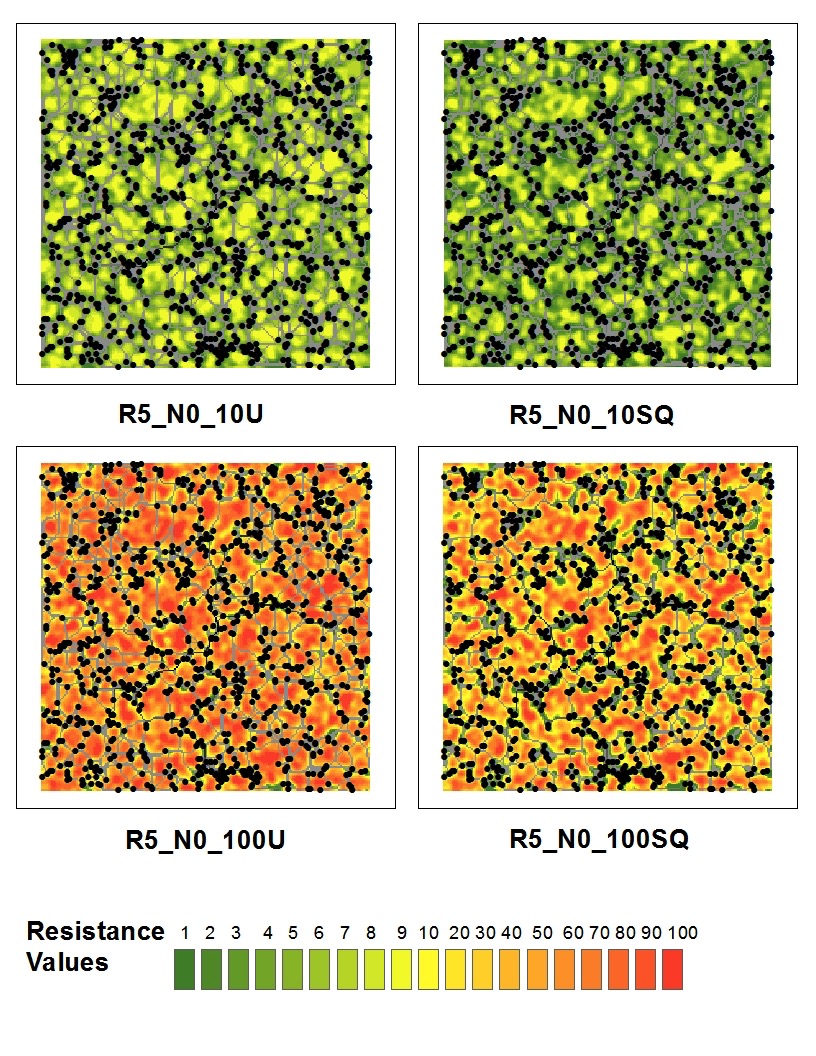


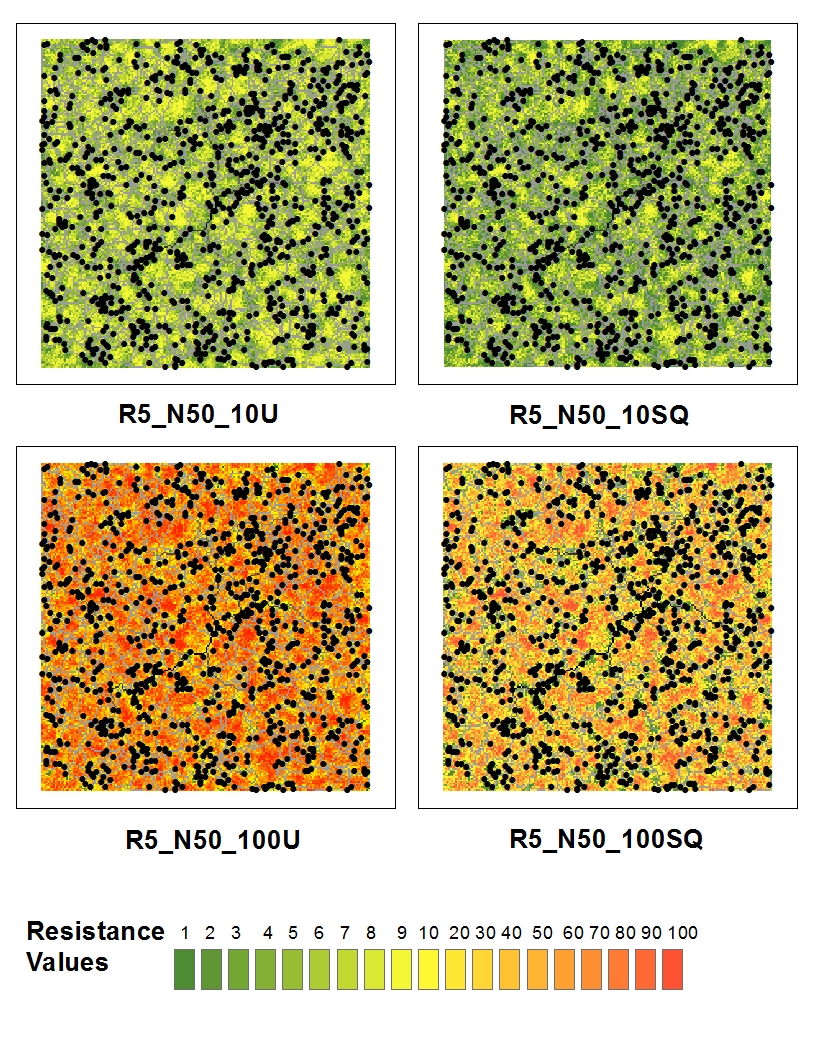


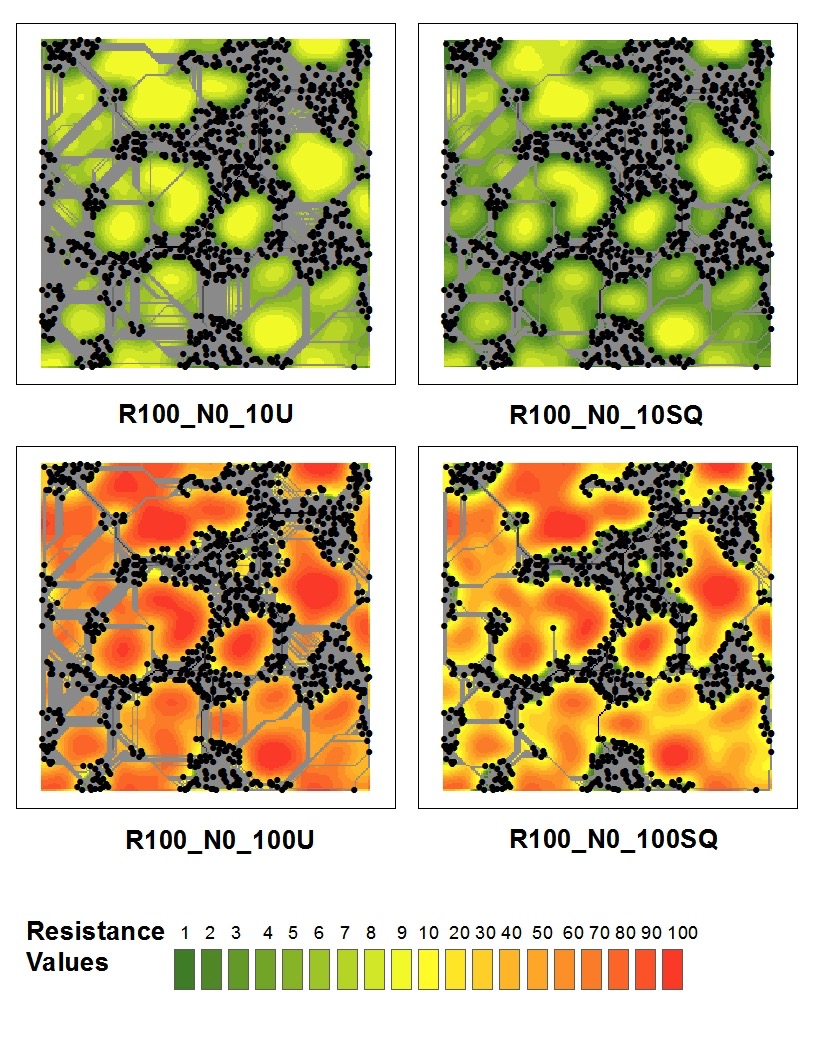


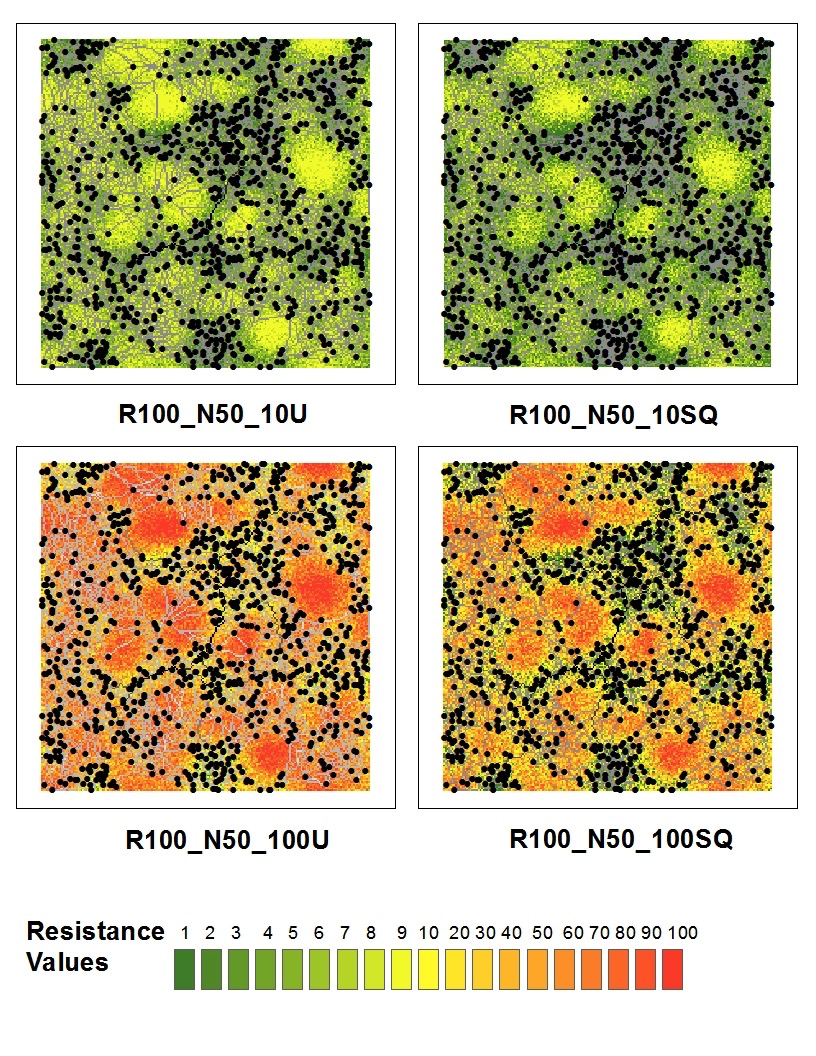


Appendix S2. Generation time for populations to reach spatial genetic equilibrium across our true resistance for replicate 1. Equilibrium was reached at the same or earlier generations in subsequent replicates, therefore, the same generation was used for all five replicates within a cluster.

| **Resistance Surface** |  |
| --- | --- |
|  | **Equilibrium Generation** |
| Cluster 1 |  |
| R5N0100SQ  R5N010U | 1000  700 |
| Cluster 2 |  |
| R5N50100SQ  R5N5010U | 600  500 |
| Cluster 3 |  |
| R100N0100SQ  R100N010U | 1000  1000 |
| Cluster 4 |  |
| R100N50100SQ  R100N5010U | 500  700 |
|  | |

Appendix S3. Simulated replications of resistance models organized by cluster, showing cell-wise correlations of true versus competing resistance models (Pearson’s *r*). The naming convention is as follows: R is the autocorrelation range (5 or 100); N is the noise introduced (0% or 50%); 10 or 100 represents the resistance value contrast (1-11 or 1-101); and U or SQ indicates the scaling of resistance values (uniform or squared). The discriminant resistance model (Dis.) refers to the model derived with a different random seed (a different hypothetical geospatial layer). True resistance models are in boldface type and have an *r* of 1 with themselves

| Resistance Surface  10U Truths | | Pearson’s *r ^1^* | | | | | | | | | | | | | | |  | | Pearson’s *r ^1^* | | | | | | | | | | |  |
| --- | --- | --- | --- | --- | --- | --- | --- | --- | --- | --- | --- | --- | --- | --- | --- | --- | --- | --- | --- | --- | --- | --- | --- | --- | --- | --- | --- | --- | --- | --- |
|  |  | *Rep1* | | | *Rep2* | | *Rep3* | | | *Rep4* | | *Rep5* | | | | Resistance Surface  100SQ Truths | | | | | *Rep1* | | | *Rep2* | | *Rep3* | | *Rep4* | *Rep5* | |
| Cluster 1 | |  | |  | | |  | |  | | | |  | | Cluster 1 | | | | |  | | |  | | |  | |  |  | |
| **R5N010U** | | **1** | | **1** | | | **1** | | **1** | | | | **1** | | R5N010U | | | | | 0.968 | | | 0.968 | | | 0.968 | | 0.968 | 0.968 | |
| R5N010SQ | | 0.968 | | 0.968 | | | 0.968 | | 0.968 | | | | 0.968 | | R5N010SQ | | | | | 1 | | | 1 | | | 1 | | 1 | 1 | |
| R5N0100U | | 1 | | 1 | | | 1 | | 1 | | | | 1 | | R5N0100U | | | | | 0.968 | | | 0.968 | | | 0.968 | | 0.968 | 0.968 | |
| R5N0100SQ | | 0.968 | | 0.968 | | | 0.968 | | 0.968 | | | | 0.968 | | **R5N0100SQ** | | | | | **1** | | | **1** | | | **1** | | **1** | **1** | |
| Dis. R5N010U ^2^ | | -0.015 | | 0.019 | | | -0.003 | | -0.008 | | | | 0.047 | | Dis. R5N0100SQ ^2^ | | | | | -0.008 | | | 0.015 | | | -0.012 | | -0.008 | 0.039 | |
| Cluster 2 | |  | |  | | |  | |  | | | |  | | Cluster 2 | | | | |  | | |  | | |  | |  |  | |
| **R5N5010U** | | **1** | | **1** | | | **1** | | **1** | | | | **1** | | R5N5010U | | | | | 0.968 | | | 0.968 | | | 0.968 | | 0.968 | 0.968 | |
| R5N5010SQ | | 0.968 | | 0.968 | | | 0.968 | | 0.968 | | | | 0.968 | | R5N5010SQ | | | | | 1 | | | 1 | | | 1 | | 1 | 1 | |
| R5N50100U | | 1 | | 1 | | | 1 | | 1 | | | | 1 | | R5N50100U | | | | | 0.968 | | | 0.968 | | | 0.968 | | 0.968 | 0.968 | |
| R5N50100SQ | | 0.968 | | 0.968 | | | 0.968 | | 0.968 | | | | 0.968 | | **R5N50100SQ** | | | | | **1** | | | **1** | | | **1** | | **1** | **1** | |
| Dis. R5N5010U ^2^ | | -0.006 | | 0.008 | | | -0.006 | | 0.004 | | | | -0.028 | | Dis. R5N50100SQ ^2^ | | | | | 0.005 | | | 0.005 | | | -0.012 | | -0.001 | -0.029 | |
| Cluster 3 | |  | |  | | |  | |  | | | |  | | Cluster 3 | | | | |  | | |  | | |  | |  |  | |
| **R100N010U** | | **1** | | **1** | | | **1** | | **1** | | | | **1** | | R100N010U | | | | | 0.968 | | | 0.968 | | | 0.968 | | 0.968 | 0.968 | |
| R100N010SQ | | 0.968 | | 0.968 | | | 0.968 | | 0.968 | | | | 0.968 | | R100N010SQ | | | | | 1 | | | 1 | | | 1 | | 1 | 1 | |
| R100N0100U | | 1 | | 1 | | | 1 | | 1 | | | | 1 | | R100N0100U | | | | | 0.968 | | | 0.968 | | | 0.968 | | 0.968 | 0.968 | |
| R100N0100SQ | | 0.968 | | 0.968 | | | 0.968 | | 0.968 | | | | 0.968 | | **R100N0100SQ** | | | | | **1** | | | **1** | | | **1** | | **1** | **1** | |
| Dis. R100N010U ^2^ | | -0.066 | | -0.020 | | | 0.110 | | 0.098 | | | | 0.067 | | Dis. R100N0100SQ ^2^ | | | | | -0.059 | | | 0.016 | | | 0.100 | | 0.102 | 0.033 | |
| Cluster 4 | |  | |  | | |  | |  | | | |  | | Cluster 4 | | | | |  | | |  | | |  | |  |  | |
| **R100N5010U** | | **1** | | **1** | | | **1** | | **1** | | | | **1** | | R100N5010U | | | | | 0.968 | | | 0.968 | | | 0.968 | | 0.968 | 0.968 | |
| R100N5010SQ | | 0. 968 | | 0.968 | | | 0.968 | | 0.968 | | | | 0.968 | | R100N5010SQ | | | | | 1 | | | 1 | | | 1 | | 1 | 1 | |
| R100N50100U | | 1 | | 1 | | | 1 | | 1 | | | | 1 | | R100N50100U | | | | | 0.968 | | | 0.968 | | | 0.968 | | 0.968 | 0.968 | |
| R100N50100SQ | | 0.968 | | 0.968 | | | 0.968 | | 0.968 | | | | 0.968 | | **R100N50100SQ** | | | | | **1** | | | **1** | | | **1** | | **1** | **1** | |
| Dis. R100N5010U ^2^ | | -0.030 | | -0.025 | | | 0.040 | | -0.046 | | | | -0.029 | | Dis. R100N50100SQ ^2^ | | | | | 0.023 | | | -0.015 | | | 0.039 | | 0.041 | -0.032 | |
|  |  | |  | | |  | |  | | |  | | |  | | | |  | | | |  | | |  | |  |  |  |  |

^1^ Pearson’s *r* is the product-moment correlation between the true resistance surface and the competing resistance hypotheses in the same landscape cluster.

Appendix S4. Simulated replications of resistance models organized by cluster, showing cost-distance correlations of true versus competing resistance models (Pearson’s *r*). The naming convention is as follows: R is the autocorrelation range (5 or 100); N is the noise introduced (0% or 50%); 10 or 100 represents the resistance value ranges (1-11 or 1-101); and U or SQ indicates the scaling of resistance values (uniform or squared). The discriminant resistance model (Dis.) refers to the model derived with a different random seed (a different hypothetical geospatial layer). True resistance models are in boldface type and have an *r* of 1 with themselves.

| Resistance Surface  10U Truths | | Pearson’s *r ^1^* | | | | | | | | | | | | | | |  | | Pearson’s *r ^1^* | | | | | | | | | | |  |
| --- | --- | --- | --- | --- | --- | --- | --- | --- | --- | --- | --- | --- | --- | --- | --- | --- | --- | --- | --- | --- | --- | --- | --- | --- | --- | --- | --- | --- | --- | --- |
|  |  | *Rep1* | | | *Rep2* | | *Rep3* | | | *Rep4* | | *Rep5* | | | | Resistance Surface  100SQ Truths | | | | | *Rep1* | | | *Rep2* | | *Rep3* | | *Rep4* | *Rep5* | |
| Cluster 1 | |  | |  | | |  | |  | | | |  | | Cluster 1 | | | | |  | | |  | | |  | |  |  | |
| **R5N010U** | | **1** | | **1** | | | **1** | | **1** | | | | **1** | | R5N010U | | | | | 0.961 | | | 0.945 | | | 0.958 | | 0.964 | 0.973 | |
| R5N010SQ | | 0.997 | | 0.996 | | | 0.997 | | 0.997 | | | | 0.997 | | R5N010SQ | | | | | 0.959 | | | 0.952 | | | 0.965 | | 0.966 | 0.978 | |
| R5N0100U | | 0.992 | | 0.991 | | | 0.994 | | 0.993 | | | | 0.993 | | R5N0100U | | | | | 0.980 | | | 0.970 | | | 0.979 | | 0.982 | 0.985 | |
| R5N0100SQ | | 0.956 | | 0.944 | | | 0.960 | | 0.963 | | | | 0.963 | | **R5N0100SQ** | | | | | **1** | | | **1** | | | **1** | | **1** | **1** | |
| Dis. R5N010U ^2^ | | 0.940 | | 0.953 | | | 0.958 | | 0.965 | | | | 0.965 | | Dis. R5N0100SQ ^2^ | | | | | 0.757 | | | 0.786 | | | 0.830 | | 0.841 | 0.77 | |
| Cluster 2 | |  | |  | | |  | |  | | | |  | | Cluster 2 | | | | |  | | |  | | |  | |  |  | |
| **R5N5010U** | | **1** | | **1** | | | **1** | | **1** | | | | **1** | | R5N5010U | | | | | 0.971 | | | 0.951 | | | 0.972 | | 0.983 | 0.983 | |
| R5N5010SQ | | 0.998 | | 0.998 | | | 0.999 | | 0.997 | | | | 0.971 | | R5N5010SQ | | | | | 0.97 | | | 0.950 | | | 0.973 | | 0.981 | 0.982 | |
| R5N50100U | | 0.993 | | 0.990 | | | 0.994 | | 0.994 | | | | 0.994 | | R5N50100U | | | | | 0.988 | | | 0.978 | | | 0.987 | | 0.991 | 0.993 | |
| R5N50100SQ | | 0.968 | | 0.949 | | | 0.972 | | 0.971 | | | | 0.971 | | **R5N50100SQ** | | | | | **1** | | | **1** | | | **1** | | **1** | **1** | |
| Dis. R5N5010U ^2^ | | 0.965 | | 0.968 | | | 0.976 | | 0.972 | | | | 0.972 | | Dis. R5N50100SQ ^2^ | | | | | 0.899 | | | 0.823 | | | 0.895 | | 0.901 | 0.863 | |
| Cluster 3 | |  | |  | | |  | |  | | | |  | | Cluster 3 | | | | |  | | |  | | |  | |  |  | |
| **R100N010U** | | **1** | | **1** | | | **1** | | **1** | | | | **1** | | R100N010U | | | | | 0.921 | | | 0.887 | | | 0.921 | | 0.911 | 0.921 | |
| R100N010SQ | | 0.990 | | 0.987 | | | 0.990 | | 0.989 | | | | 0.989 | | R100N010SQ | | | | | 0.922 | | | 0.922 | | | 0.951 | | 0.952 | 0.943 | |
| R100N0100U | | 0.986 | | 0.985 | | | 0.989 | | 0.993 | | | | 0.993 | | R100N0100U | | | | | 0.967 | | | 0.939 | | | 0.963 | | 0.943 | 0.966 | |
| R100N0100SQ | | 0.901 | | 0.876 | | | 0.927 | | 0.915 | | | | 0.915 | | **R100N0100SQ** | | | | | **1** | | | **1** | | | **1** | | **1** | **1** | |
| Dis. R100N010U ^2^ | | 0.796 | | 0.822 | | | 0.894 | | 0.913 | | | | 0.912 | | Dis. R100N0100SQ ^2^ | | | | | 0.921 | | | 0.484 | | | 0.600 | | 0.746 | 0.521 | |
| Cluster 4 | |  | |  | | |  | |  | | | |  | | Cluster 4 | | | | |  | | |  | | |  | |  |  | |
| **R100N5010U** | | **1** | | **1** | | | **1** | | **1** | | | | **1** | | R100N5010U | | | | | 0.922 | | | 0.862 | | | 0.925 | | 0.906 | 0.935 | |
| R100N5010SQ | | 0.997 | | 0.996 | | | 0.997 | | 0.997 | | | | 0.997 | | R100N5010SQ | | | | | 0.950 | | | 0.864 | | | 0.925 | | 0.902 | 0.939 | |
| R100N50100U | | 0.979 | | 0.976 | | | 0.981 | | 0.975 | | | | 0.975 | | R100N50100U | | | | | 0.958 | | | 0.938 | | | 0.975 | | 0.967 | 0.975 | |
| R100N50100SQ | | 0.905 | | 0.873 | | | 0.922 | | 0.904 | | | | 0.904 | | **R100N50100SQ** | | | | | **1** | | | **1** | | | **1** | | **1** | **1** | |
| Dis. R100N5010U ^2^ | | 0.872 | | 0.873 | | | 0.923 | | 0.797 | | | | 0.797 | | Dis. R100N50100SQ ^2^ | | | | | 0.57 | | | 0.485 | | | 0.681 | | 0.742 | 0.505 | |
|  |  | |  | | |  | |  | | |  | | |  | | | |  | | | |  | | |  | |  |  |  |  |

^1^ Pearson’s *r* is the product-moment correlation between effective distances calculated on the true resistance surface and the competing resistance hypotheses in the same landscape cluster.

Appendix S5. Relationship between cost distance and genetic distance for Replicate 1 of the R5N0_100SQ landscape; (A) untransformed, and (B) with cost distances natural log-transformed. Red lines are the fitted regression lines; green lines are loess curves. R^2^ for untransformed and transformed regression lines are 0.462 and 0.650 respectively.


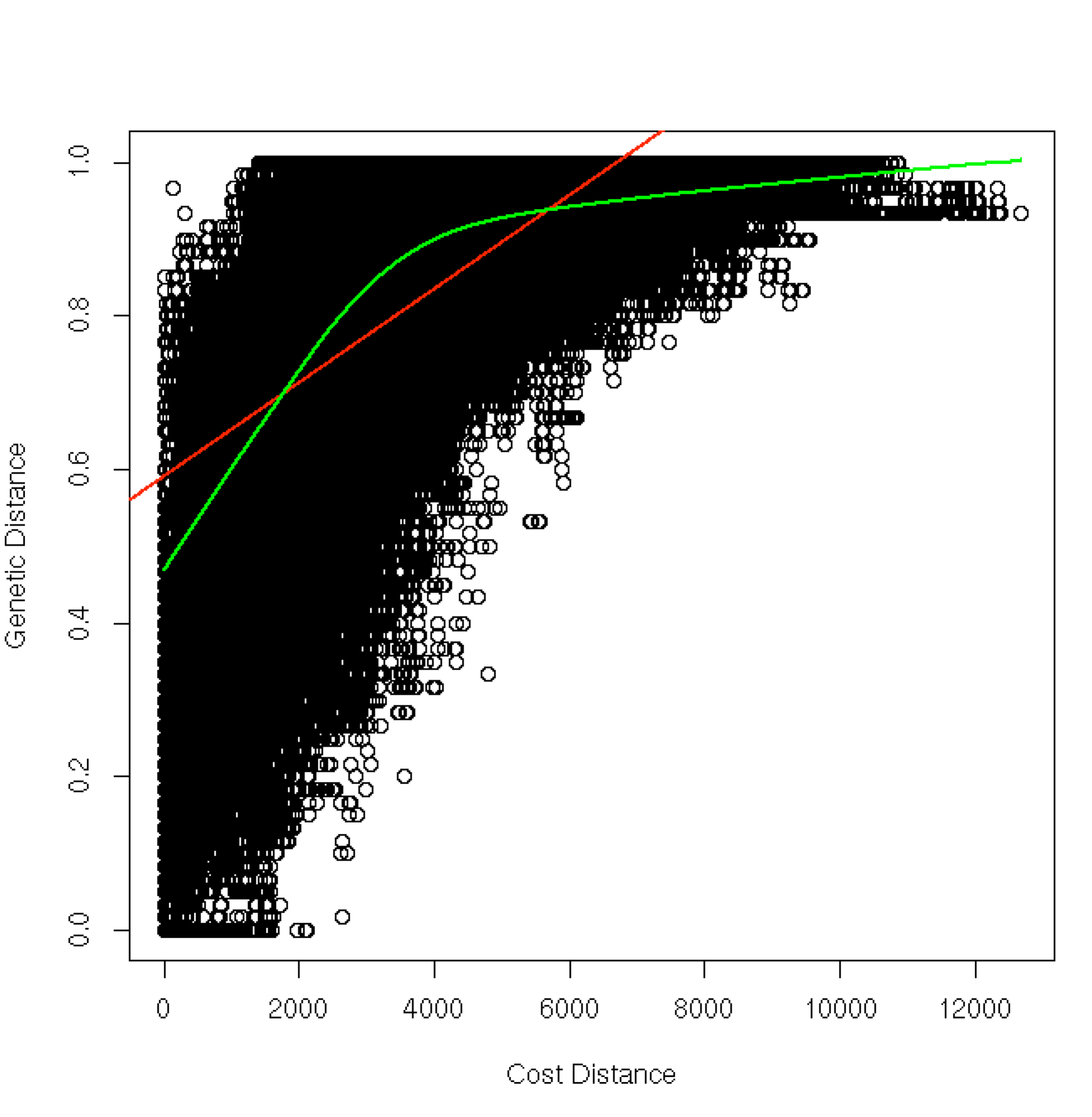


a)

­


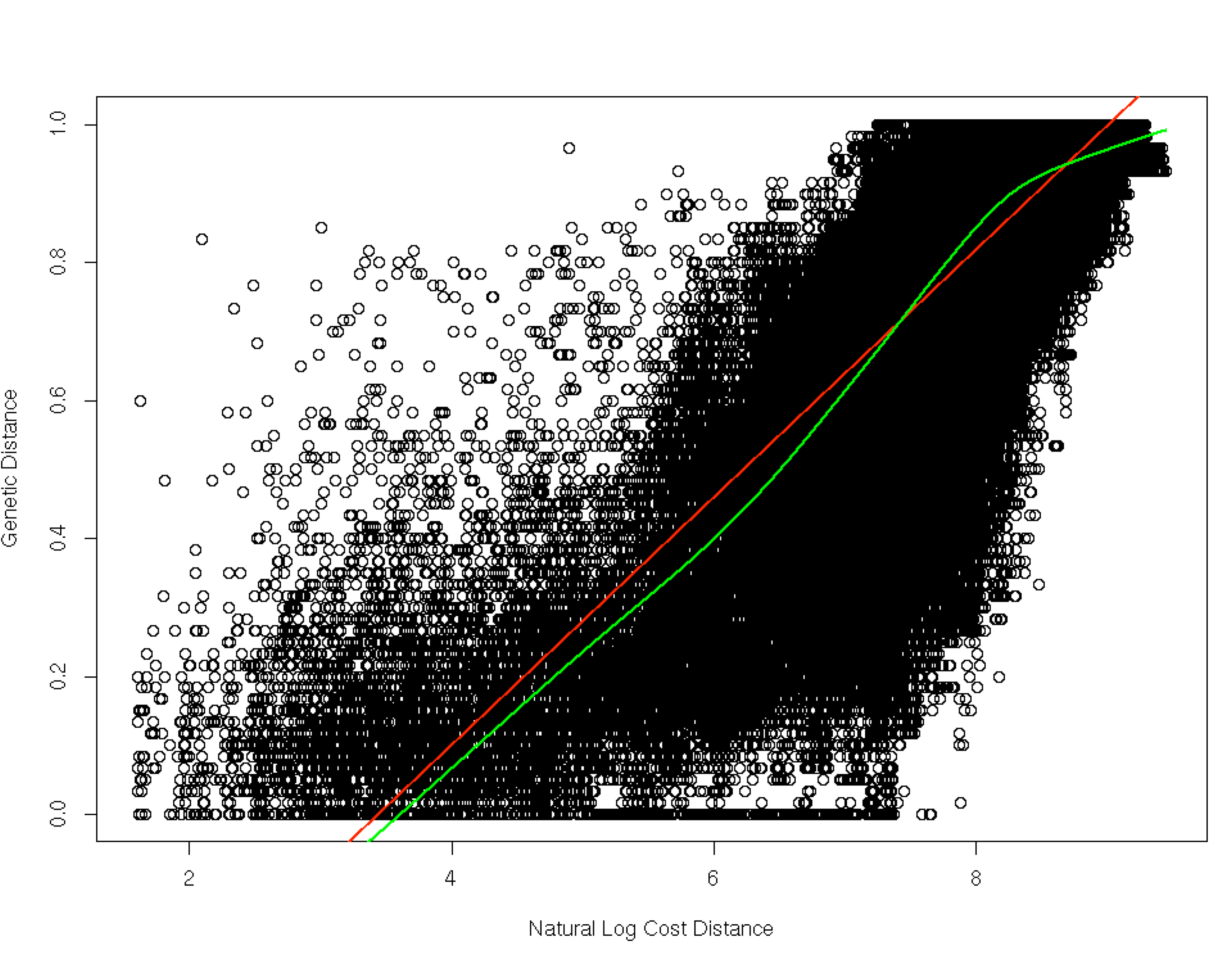


b)

Appendix S6. Simulated replications of resistance models organized by cluster, showing Moran’s *I*. The naming convention is as follows: R is the autocorrelation range (5 or 100); N is the noise introduced (0% or 50%); 10 or 100 represents the resistance value ranges (1-11 or 1-101); and U or SQ indicates the scaling of resistance values (uniform or squared). The discriminant resistance model (Dis.) refers to the model derived with a different random seed (a different hypothetical geospatial layer). True resistance models are in boldface type.

| Resistance Surface  10U Truths | | Moran’s *I* | | | | | | | | | | | | | | |  | | Moran’s *I* | | | | | | | | | | |  |
| --- | --- | --- | --- | --- | --- | --- | --- | --- | --- | --- | --- | --- | --- | --- | --- | --- | --- | --- | --- | --- | --- | --- | --- | --- | --- | --- | --- | --- | --- | --- |
|  |  | *Rep1* | | | *Rep2* | | *Rep3* | | | *Rep4* | | *Rep5* | | | | Resistance Surface  100SQ Truths | | | | | *Rep1* | | | *Rep2* | | *Rep3* | | *Rep4* | *Rep5* | |
| Cluster 1 | |  | |  | | |  | |  | | | |  | | Cluster 1 | | | | |  | | |  | | |  | |  |  | |
| **R5N010U** | | **0.892** | | **0.891** | | | **0.891** | | **0.891** | | | | **0.888** | | R5N010U | | | | | 0.892 | | | 0.891 | | | 0.891 | | 0.891 | 0.888 | |
| R5N010SQ | | 0.887 | | 0.882 | | | 0.884 | | 0.883 | | | | 0.881 | | R5N010SQ | | | | | 0.887 | | | 0.882 | | | 0.884 | | 0.883 | 0.881 | |
| R5N0100U | | 0.892 | | 0.891 | | | 0.891 | | 0.891 | | | | 0.888 | | R5N0100U | | | | | 0.892 | | | 0.891 | | | 0.891 | | 0.891 | 0.888 | |
| R5N0100SQ | | 0.887 | | 0.882 | | | 0.884 | | 0.883 | | | | 0.881 | | **R5N0100SQ** | | | | | **0.887** | | | **0.882** | | | **0.884** | | **0.883** | **0.881** | |
| Dis. R5N010U ^2^ | | 0.903 | | 0.903 | | | 0.903 | | 0.900 | | | | 0.904 | | Dis. R5N0100SQ ^2^ | | | | | 0.885 | | | 0.884 | | | 0.883 | | 0.881 | 0.882 | |
| Cluster 2 | |  | |  | | |  | |  | | | |  | | Cluster 2 | | | | |  | | |  | | |  | |  |  | |
| **R5N5010U** | | **0.434** | | **0.435** | | | **0.432** | | **0.341** | | | | **0.336** | | R5N5010U | | | | | 0.434 | | | 0.435 | | | 0.432 | | 0.341 | 0.336 | |
| R5N5010SQ | | 0.420 | | 0.420 | | | 0.420 | | 0.327 | | | | 0.321 | | R5N5010SQ | | | | | 0.420 | | | 0.420 | | | 0.420 | | 0.327 | 0.321 | |
| R5N50100U | | 0.434 | | 0.435 | | | 0.432 | | 0.341 | | | | 0.336 | | R5N50100U | | | | | 0.434 | | | 0.435 | | | 0.432 | | 0.341 | 0.336 | |
| R5N50100SQ | | 0.420 | | 0.420 | | | 0.420 | | 0.327 | | | | 0.321 | | **R5N50100SQ** | | | | | **0.420** | | | **0.420** | | | **0.420** | | **0.327** | **0.321** | |
| Dis. R5N5010U ^2^ | | 0.494 | | 0.496 | | | 0.498 | | 0.492 | | | | 0.497 | | Dis. R5N50100SQ ^2^ | | | | | 0.315 | | | 0.420 | | | 0.418 | | 0.321 | 0.417 | |
| Cluster 3 | |  | |  | | |  | |  | | | |  | | Cluster 3 | | | | |  | | |  | | |  | |  |  | |
| **R100N010U** | | **0.991** | | **0.990** | | | **0.991** | | **0.992** | | | | **0.990** | | R100N010U | | | | | 0.991 | | | 0.990 | | | 0.991 | | 0.992 | 0.990 | |
| R100N010SQ | | 0.990 | | 0.987 | | | 0.990 | | 0.992 | | | | 0.989 | | R100N010SQ | | | | | 0.990 | | | 0.987 | | | 0.990 | | 0.992 | 0.989 | |
| R100N0100U | | 0.991 | | 0.990 | | | 0.991 | | 0.992 | | | | 0.990 | | R100N0100U | | | | | 0.991 | | | 0.990 | | | 0.991 | | 0.992 | 0.990 | |
| R100N0100SQ | | 0.990 | | 0.987 | | | 0.990 | | 0.992 | | | | 0.989 | | **R100N0100SQ** | | | | | **0.990** | | | **0.987** | | | **0.990** | | **0.992** | **0.989** | |
| Dis. R100N010U ^2^ | | 0.991 | | 0.992 | | | 0.993 | | 0.991 | | | | 0.992 | | Dis. R100N0100SQ ^2^ | | | | | 0.990 | | | 0.990 | | | 0.992 | | 0.989 | 0.993 | |
| Cluster 4 | |  | |  | | |  | |  | | | |  | | Cluster 4 | | | | |  | | |  | | |  | |  |  | |
| **R100N5010U** | | **0.485** | | **0.457** | | | **0.468** | | **0.381** | | | | **0.370** | | R100N5010U | | | | | 0.485 | | | 0.457 | | | 0.468 | | 0.381 | 0.370 | |
| R100N5010SQ | | 0.472 | | 0.450 | | | 0.456 | | 0.360 | | | | 0.342 | | R100N5010SQ | | | | | 0.472 | | | 0.450 | | | 0.456 | | 0.360 | 0.342 | |
| R100N50100U | | 0.485 | | 0.457 | | | 0.468 | | 0.381 | | | | 0.370 | | R100N50100U | | | | | 0.485 | | | 0.457 | | | 0.468 | | 0.381 | 0.370 | |
| R100N50100SQ | | 0.472 | | 0.450 | | | 0.456 | | 0.360 | | | | 0.342 | | **R100N50100SQ** | | | | | **0.472** | | | **0.450** | | | **0.456** | | **0.360** | **0.342** | |
| Dis. R100N5010U ^2^ | | 0.532 | | 0.528 | | | 0.543 | | 0.524 | | | | 0.528 | | Dis. R100N50100SQ ^2^ | | | | | 0.352 | | | 0.456 | | | 0.466 | | 0.342 | 0.455 | |
|  |  | |  | | |  | |  | | |  | | |  | | | |  | | | |  | | |  | |  |  |  |  |

Appendix S7. Correlation length (A) and patch cohesion metrics (B) for the simulated resistance models.


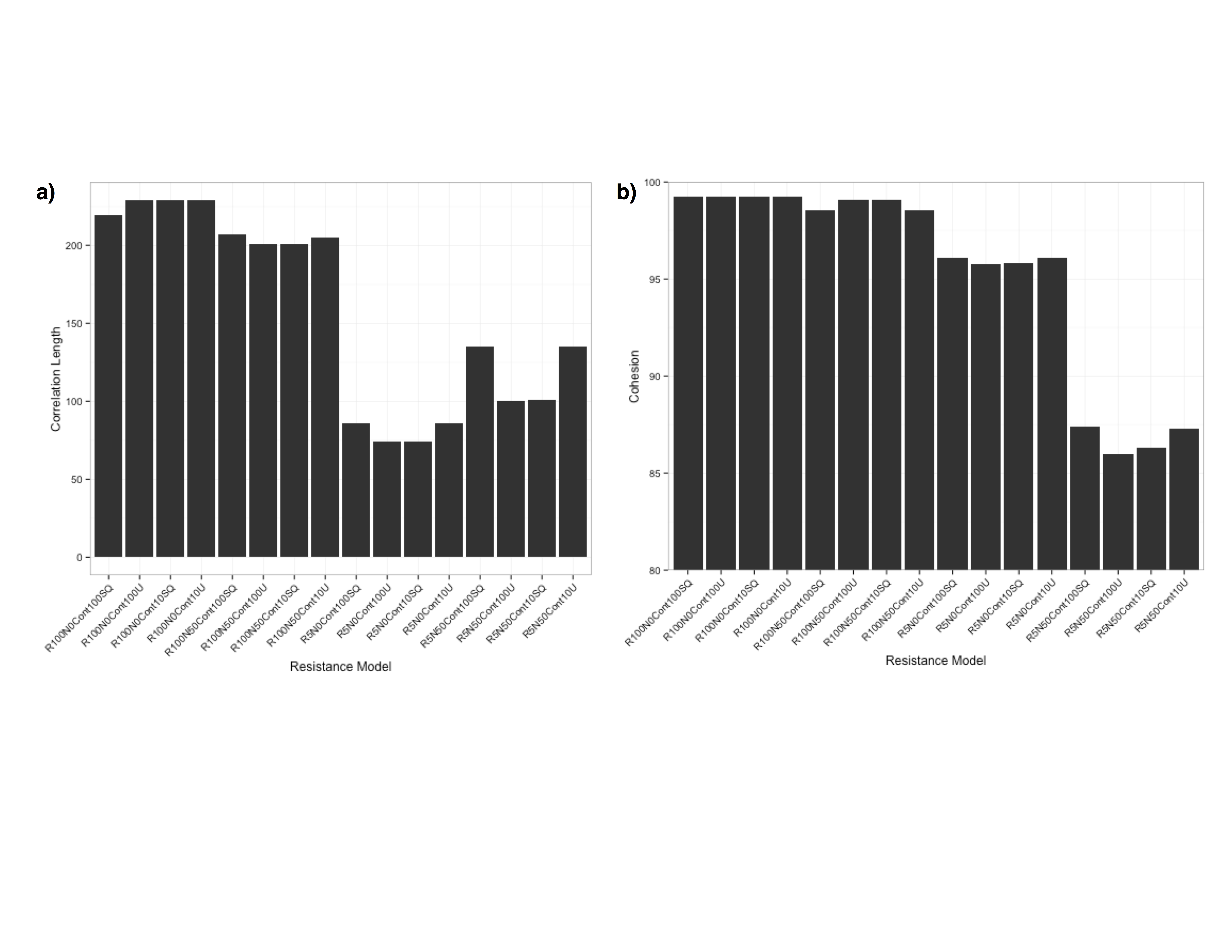


Appendix S8. Model performance (proportion of runs in which true resistance model outperformed all other resistance models in a cluster) by cluster. Proportion success was averaged across the $\bar{RS}$ and simple Mantel *r* methods. Causal modeling results have been omitted. Truth 10U and truth 100SQ results were pooled. “Log” refers to whether the cost distances were log transformed to linearize their relationship with genetic distance. “Eq” indicates whether the genetic data used were from a generation that had reached genetic equilibrium or whether a prior generation was used. Values in table are means with standard deviations in parentheses.

|  | **Log=N**  **Eq=N** | **Log=N**  **Eq=Y** | **Log=Y**  **Eq=N** | **Log=Y**  **Eq=Y** | **Across approaches** |
| --- | --- | --- | --- | --- | --- |
| **R5N0** | 0.431 (0.400) | 0.526 (0.452) | 0.588 (0.408) | 0.635 (0.365) | **0.545 (0.406)** |
| **R5N50** | 0.531 (0.352) | 0.480 (0.377) | 0.693 (0.309) | 0.543 (0.328) | **0.561 (0.345)** |
| **R100N0** | 0.447 (0.478) | 0.229 (0.394) | 0.305 (0.377) | 0.500 (0.513) | **0.370 (0.449)** |
| **R100N50** | 0.373 (0.443) | 0.493 (0.489) | 0.478 (0.454) | 0.523 (0.494) | **0.467 (0.465)** |
| **Across simulations** | **0.446 (0.417)** | **0.432 (0.439)** | **0.516 (0.409)** | **0.550 (0.427)** | **0.486 (0.424)** |

Appendix S9. Scatterplot showing the cell-wise correlation between each resistance model in a cluster and the true resistance model (x-axis), plotted against the cost-distance correlation between each resistance model in a cluster and the true resistance model (y-axis). “Alt.resolution” refers to the alternative resistance models that have a different contrast of resistance values (1-11 or 1-101) from the true model. “Alt.scale” refers to alternative resistance models that have a different scaling of resistance values (U or SQ) from the true model. “Alt.scale.res” refers to alternative resistance models that have a different resistance value contrast and scaling from the true model. “Discriminant” refers to the discriminant resistance models in each cluster. “Truth” refers to the true resistance model in each cluster. “100SQ” and “10U” refer to the resistance model used as the truth in a cluster.


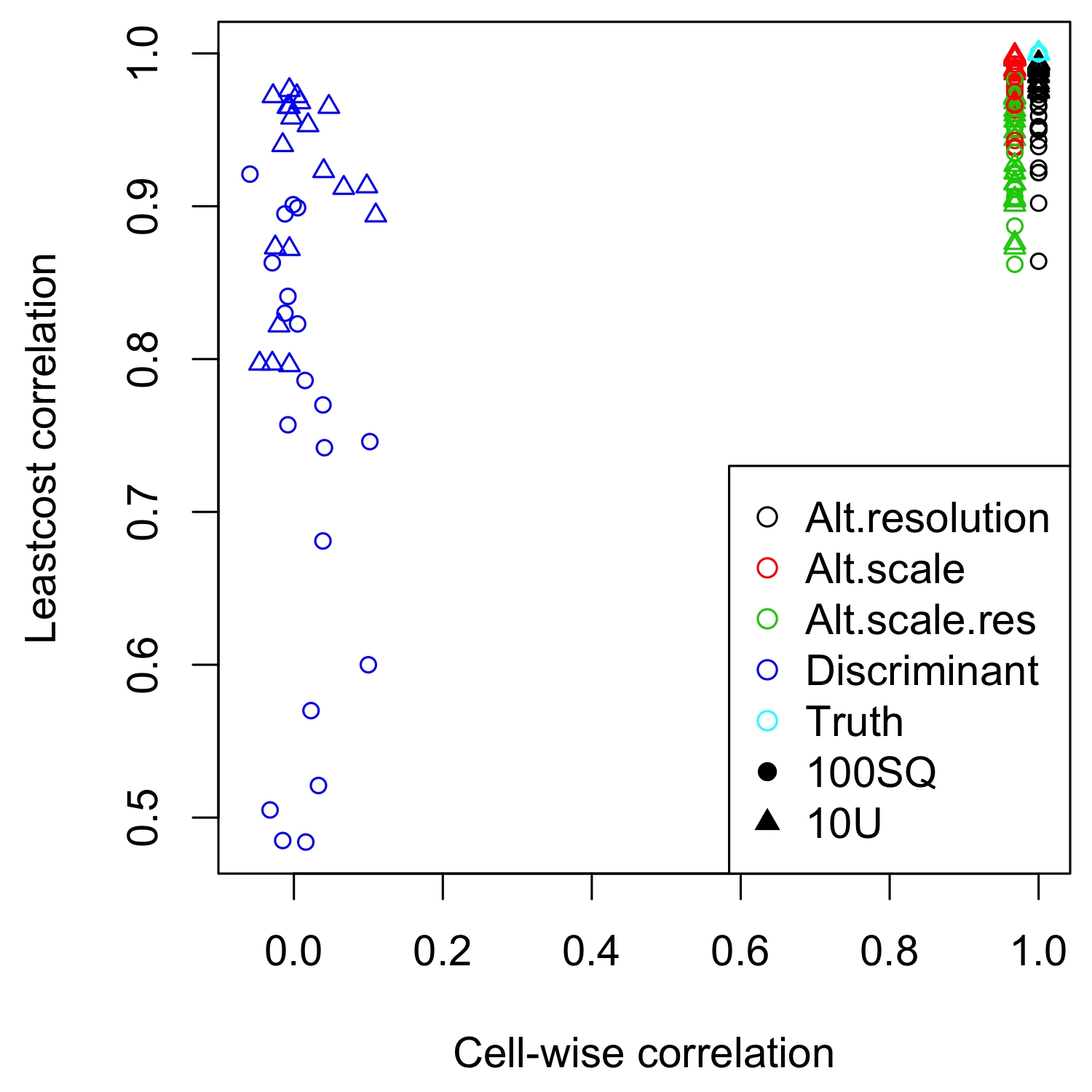


Appendix S10A. Cost-distance correlations plotted against model performance for the discriminant resistance models and for one of the alternative resistance models for each cluster. The alternative resistance models were chosen to be the least similar to that of the truth (e.g. having a different contrast and scaling of resistance values). Results shown are for the approach with the highest success rate (linearized or not, and at spatial genetic equilibrium or not). The four Mantel-based tests used are “CM.p05” (refers to CM with an (α =0.05)), “CM.p005” (refers to CM with an (α =0.005)), “RS” (refers to relative support), “simpleR” (refers to simple Mantel *r*).


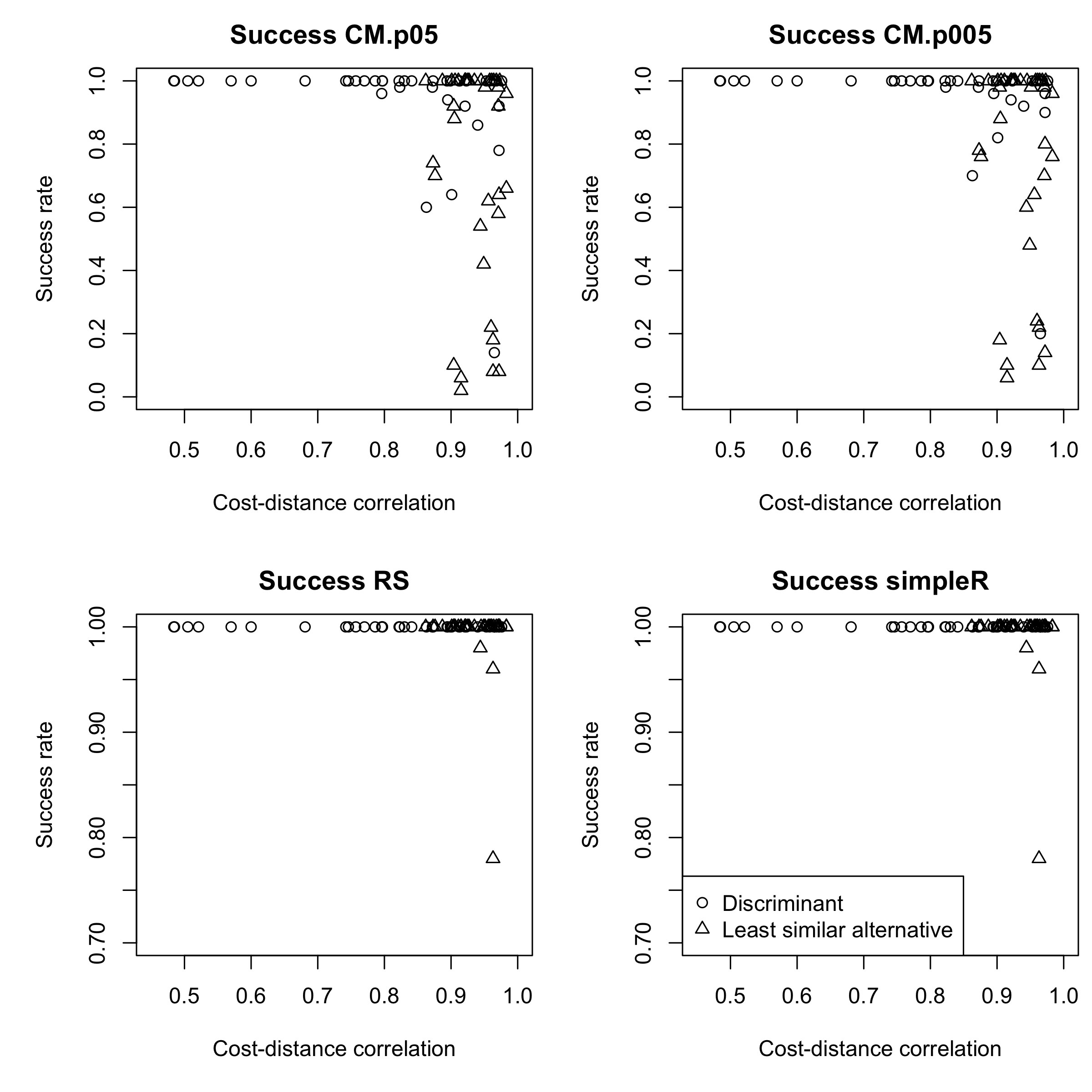


Appendix S10B. Cost-distance correlations plotted against model performance for the discriminant resistance models (circles) and for one of the alternative resistance models for each cluster (triangles). The alternative resistance models were chosen to be the least similar to that of the truth (e.g. having a different contrast and scaling of resistance values). Results shown are for all approaches; ‘Equilibrium,log” refers to runs where a generation at spatial genetic equilibrium was used and the cost to genetic distance relationship was linearized, “Equilibrium, no log”, refers to runs where a generation at spatial genetic equilibrium was used and the cost to genetic distance relationship was not linearized, ‘Non-equilibrium,log” refers to runs where a generation at non-spatial genetic equilibrium was used and the cost to genetic distance relationship was linearized, “Non-equilibrium, no log”, refers to runs where a generation at non-spatial genetic equilibrium was used and the cost to genetic distance relationship was not lin­­earized.


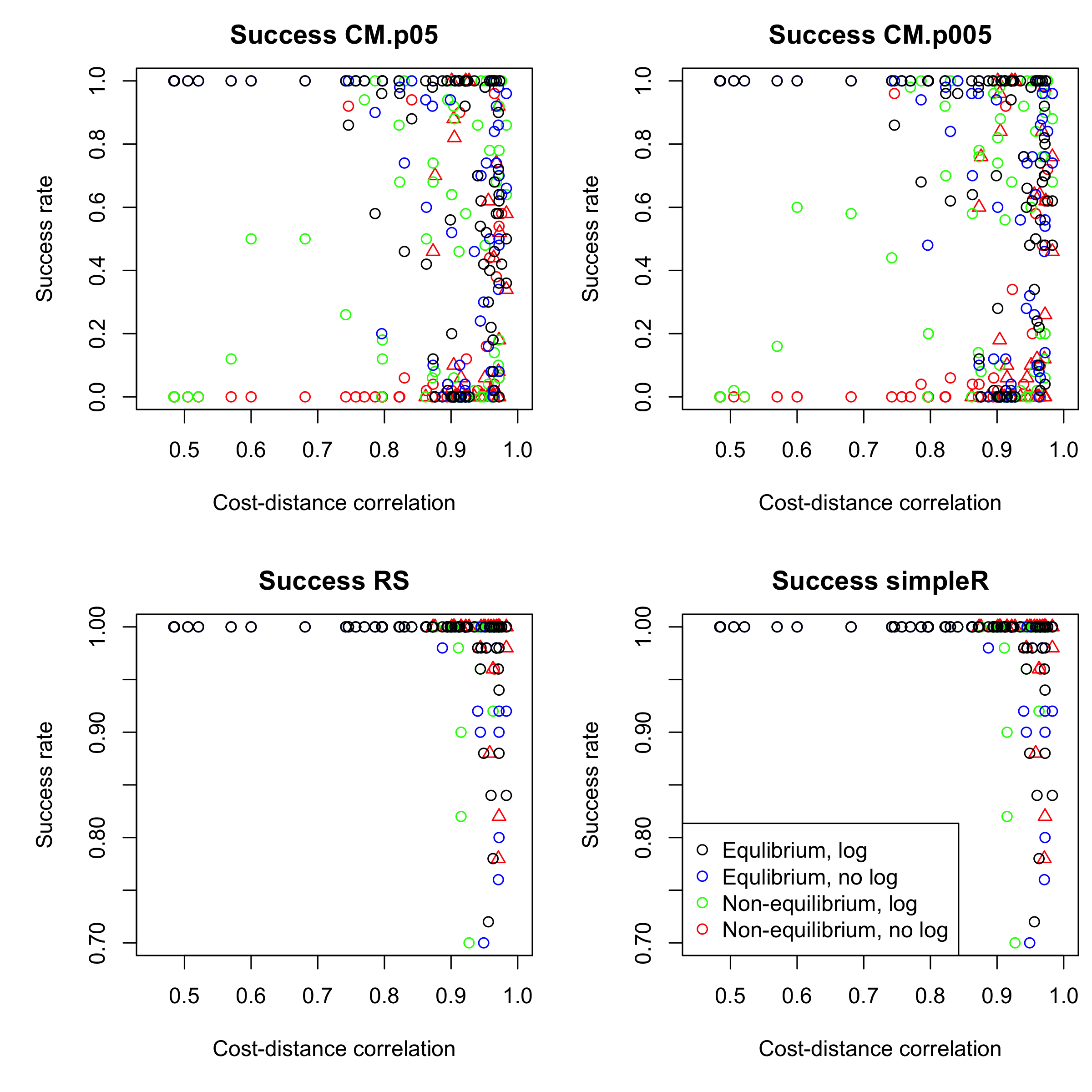


Appendix S11. Model performance as judged by the proportion of MC replicates in which the true resistance model outperformed all other resistance models in a cluster. Proportion success is averaged across the $\bar{RS}$ and simple Mantel *r* methods. Causal modeling results have been omitted. Truth 10U and 100SQ results are presented separately. The first column presents model performance by landscape cluster. The second column presents performance by all possible combinations of linearity and spatial genetic equilibrium. *Log* refers to whether the cost-distances were log-transformed to linearize their relationship with genetic distance. *Eq* indicates whether the genetic data used was from a generation that had reached spatial genetic equilibrium or whether a prior generation was used.

a

Appendix S12. Success rate of Mantel-based methods when comparing amongst all resistance models within a cluster, including those based on different parameterizations of the same landscape variable (i.e., proportion of MC replicates in which the true resistance model outperformed all other resistance models), and **using a random subsample of 100 individuals**. Truth 10U and truth 100SQ results are pooled. *Log* refers to whether the cost distances were log-transformed to better linearize their relationship with genetic distance. *Eq* indicates whether the genetic data used was from a generation that had reached genetic equilibrium or whether a prior generation was used. Numbers in table are means with standard deviations in parentheses.

|  | **Log=N**  **Eq=N** | **Log=N**  **Eq=Y** | **Log=Y**  **Eq=N** | **Log=Y**  **Eq=Y** | **Mean across approaches** |
| --- | --- | --- | --- | --- | --- |
| **Causal modeling**  **(α =0.05)** | 0.093 (0.136) | 0.337 (0.412) | 0.229 (0.303) | 0.418 (0.436) | **0.269 (0.361)** |
| **Causal modeling**  **(α = 0.005)** | 0.155 (0.212) | 0.346 (0.423) | 0.290 (0.329) | 0.428 (0.452) | **0.305 (0.376)** |
| **Relative support (RS)** | 0.362 (0.328) | 0.372 (0.384) | 0.423 (0.316) | 0.500 (0.415) | **0.414 (0.364)** |
| **Simple Mantel *r*** | 0.410 (0.349) | 0.456 (0.431) | 0.451 (0.342) | 0.509 (0.440) | **0.456 (0.391)** |
| **Mean across methods** | **0.255 (0.300)** | **0.377 (0.412)** | **0.348 (0.333)** | **0.464 (0.434)** | **0.361 (0.380)** |

Appendix S13. Success rate of Mantel-based methods to select the true resistance model over the discriminant resistance model (i.e., proportion of MC replicates in which true model outperformed discriminant model) when only those two models are included as competing hypotheses, and using a random subsample of 100 individuals. Truth 10U and truth 100SQ results are pooled. *Log* refers to whether the cost distances were log transformed to linearize their relationship with genetic distance. *Eq* indicates whether the genetic data used was from a generation that had reached genetic equilibrium or whether a prior generation was used. Numbers in table are means with standard deviations in parentheses.

|  | **Log=N**  **Eq=N** | **Log=N**  **Eq=Y** | **Log=Y**  **Eq=N** | **Log=Y**  **Eq=Y** | **Mean across approaches** |
| --- | --- | --- | --- | --- | --- |
| **Causal modeling**  **(α =0.05)** | 0.346 (0.312) | 0.833 (0.268) | 0.708 (0.292) | 0.823 (0.260) | **0.677 (0.344)** |
| **Causal modeling**  **(α = 0.005)** | 0.486 (0.336) | 0.866 (0.249) | 0.791 (0.267) | 0.879 (0.226) | **0.755 (0.314)** |
| **Relative support (RS)** | 0.967 (0.159) | 0.942 (0.178) | 0.973 (0.158) | 0.947 (0.179) | **0.957 (0.168)** |
| **Simple Mantel *r*** | 0.967 (0.159) | 0.942 (0.178) | 0.973 (0.158) | 0.947 (0.179) | **0.957 (0.168)** |
| **Mean across methods** | **0.691 (0.378)** | **0.896 (0.225)** | **0.861 (0.253)** | **0.899 (0.218)** | **0.837 (0.288)** |
