# Supplementary material for: Using simulations to evaluate Mantel‐based methods for assessing landscape resistance to gene flow
Source: Ecol Evol. 2016 May 21;6(12):4115–28. doi: 10.1002/ece3.2154 (PMC4879002; doi:10.1002/ece3.2154)
Supplement: Supplementary file 2 — Appendix S14. R and Python code for Mantel‐based test simulation analysis. [file ECE3-6-4115-s002.docx]

# Appendix S14. R and Python code for Mantel-based test simulation analysis

# R code for generating landscapes and resistance models

## Source code for 'landscapes' function

landscapes <- function(size = 25, pnoise = 0.5, plot.it=TRUE, seed = 8, R=c(5,10,15,20))
{

 # Initialize random number generator

 RNGkind(kind = NULL, normal.kind = NULL)
 set.seed(seed, kind = NULL)

 # Autocorrelated pattern

 Xinit <- matrix(rnorm(size^2), size,size)
 Xmean <- list()

 for(i in 1:length(R)){
 X <- Xinit
 for(r in 1:R[i]){
 X2 <- X

 for(x in 1:(nrow(X))){
 for(y in 1:(ncol(X))){
 X2[x,y]<-
 mean(X[(((x-1+size):(x+1+size))+size)%%size,
 (((y-1+size):(y+1+size))+size)%%size], na.rm=TRUE)
 }
 }
 X <- X2
 }

 Xmean[[i]] <- matrix(scale(as.vector(X)),size,size)
 }

 if(plot.it==TRUE)
 {
 par(mfrow=c(2,2), omi=c(0,0,0,0), mai=c(0.2,0.2,0.2,0.2))
 for(i in 1:length(R)) image(Xmean[[i]], axes=FALSE, asp=1, col=gray(1:11/12))
 par(mfrow=c(1,1))
 }

 # Adding local random noise

 Xsum <- list()
 X <- matrix(rnorm(size*size), size,size)

 p <- pnoise

 for(i in 1:length(R))
 {
 X2 <- p*X + (1-p)*Xmean[[i]]
 Xsum[[i]] <- matrix(scale(as.vector(X2)),size,size)
 }

 if(plot.it==TRUE)
 {
 par(mfrow=c(2,2), omi=c(0,0,0,0), mai=c(0.2,0.2,0.2,0.2))
 for(i in 1:length(R)) image(Xsum[[i]], axes=FALSE, asp=1, col=gray(1:11/12))
 par(mfrow=c(1,1))
 }

 Xsum
}

## Running 'landscapes' function

'landscapes' function arguments: > size: number of cells in each dimension (a single number)
 > pnoise: proportion of random noise
 > plot.it: Flag whether map should be plotted. Will be plotted in matrix of 2 x 2 maps
 > seed: random seed (a single integer). The landscapes simulated will only differ in the range R
 > R: range parameter (vector: for each value, one landscape will be simulated)

Example below is for creating a 200x200 pixel landscape with a range of 5 and 50% noise

require (raster)
Pattern0 <- landscapes(size=200, R =5,pnoise=0.5,seed=6)

## Transforming simulated patterns into spatial resistance surfaces

Import list 'Pattern' that contains series of n = length (R) simulated maps into raster stack, Series0

Series0 <- stack(lapply(Pattern0, raster))
 plot(Series0)

Modify raster values to manipulate to continuous resistance values. First chunk of code creates continuous values with a uniform distribution from 0-1

Model.dist<-runif(dim(Series0)[1]*dim(Series0)[2],min=0,max=1) # create uniform values with min=0 and max = 1.
 Model.dist10<-(Model.dist*9)+1 # Rescale from 1-10
 Model.dist10 <- sort(Model.dist10)
 hist(Model.dist10)

 tmp <- list()
 for(i in 1: dim(Series0)[3])
 {
 tmp[[i]] <- raster(Series0, layer=i)
 values(tmp[[i]])[order(values(tmp[[i]]))] <- Model.dist10

 }

 Series0.unif.10 <- stack(tmp)

 hist(Series0.unif.10)
 plot(Series0.unif.10)
 table(values(Series0.unif.10))

The following chunk of code creates a continuous surface with a uniform distribution from 1-100

Model.dist100<-(Model.dist*99)+1

 Model.dist100 <- sort(Model.dist100)
 hist(Model.dist100)

 tmp <- list()
 for(i in 1: dim(Series0)[3])
 {
 tmp[[i]] <- raster(Series0, layer=i)
 values(tmp[[i]])[order(values(tmp[[i]]))] <- Model.dist100

 }

 Series0.unif.100 <- stack(tmp)

 hist(Series0.unif.100)
 plot(Series0.unif.100)
 table(values(Series0.unif.100))

The following chunk of code creates a continuous surface with a squared distribution from 1-10

Model.distsq10<-((Model.dist^2)*9)+1
Model.distsq10 <- sort(Model.distsq10)
hist(Model.distsq10)

tmp <- list()
for(i in 1: dim(Series0)[3])
{
 tmp[[i]] <- raster(Series0, layer=i)
 values(tmp[[i]])[order(values(tmp[[i]]))] <- Model.distsq10
}

Series0.uneven.10 <- stack(tmp)

hist(Series0.uneven.10)
plot(Series0.uneven.10)
table(values(Series0.uneven.10))

The following chunk of code creates a continuous surface with a squared distribution from 1-100

Model.distsq100<-((Model.dist^2)*99)+1
Model.distsq100 <- sort(Model.distsq100)
hist(Model.distsq100)

tmp <- list()
for(i in 1: dim(Series0)[3])
{
 tmp[[i]] <- raster(Series0, layer=i)
 values(tmp[[i]])[order(values(tmp[[i]]))] <- Model.distsq100
}

Series0.uneven.100 <- stack(tmp)

hist(Series0.uneven.100)
plot(Series0.uneven.100)
table(values(Series0.uneven.100))

## Extract and write out rasters as ascii files

Code below uses our naming convention for a resistance surface with a range of 5 and 50% noise

require(rgdal)

R5N50Cont10U<-raster(Series0.unif.10, layer=1)
writeRaster(R5N50Cont10U, filename="R5N50Cont10U.asc", format="ascii", overwrite=T)
plot(R5N50Cont10U)

R5N50Cont100U<- raster(Series0.unif.100, layer=1)
writeRaster(R5N50Cont100U, filename="R5N50Cont100U.asc", format="ascii",overwrite=T)
plot(R5N50Cont100U)

R5N50Cont10SQ<- raster(Series0.uneven.10, layer=1)
writeRaster(R5N50Cont10SQ, filename="R5N50Cont10SQ.asc", format="ascii",overwrite=T)
plot(R5N50Cont10SQ)

R5N50Cont100SQ<-raster(Series0.uneven.100, layer=1)
writeRaster(R5N50Cont100SQ, filename="R5N50Cont100SQ.asc", format="ascii",overwrite=T)
plot(R5N50Cont100SQ)

# R code for distributing individuals across landscapes and calculating cost distances

Load packages

library(maptools)
library(raster)
library(MASS)
library(gdistance)

Set working directory and list landscapes. Working directory should contain all the landscapes (as ascii files) across which individuals will be distributed. No other files should exist in this folder.

setwd("")
landscapes <- list.files(getwd()) # create list of landscape .asc file names

Set parameters

no.individuals <- 1000 # the desired number of individuals to be placed in each landscape
proportion.habitat <- 0.4 # the proportion of the landscape that will be considered habitat
same.start.cell <- FALSE # FALSE if every individual must start in a different cell (i.e., sampling without replacement from habitat cells)
 # TRUE if individuals may share a starting cell (i.e., sampling with replacement)
neighbors <- 8 # neighbor cell rule (4, 8, 12, or 16)

Distribute individuals across all landscapes in working directory and calculate cost distances between each pair of individuals

for(i in 1:length(landscapes)) {
 table <- data.frame(readAsciiGrid(landscapes[i])) # read in asc file in dataframe for (each row is a cell in the landscape)
 names(table) <- c("resistance", "x", "y") # name dataframe columns
 attach(table)
 cutoff <- as.numeric(quantile(resistance, proportion.habitat)) # find resistance value cutoff associated with chosen proportion.habitat
 habitat.rows <- which(resistance<=cutoff) # get indices of rows that represent "habitat" cells
 sample.rows <- sample(habitat.rows, size=no.individuals, replace=same.start.cell) # pick a random habitat cell for each individual
 xy.data <- table[sample.rows,2:3] # create table of randomly selected habitat cells
 names(xy.data) <- c("XCOORD","YCOORD")
 temp <- paste("xydata_",landscapes[i], sep="") # new file name
 filename <- paste(substr(temp,1,nchar(temp)-4),".csv", sep="") # remove ".asc" from name and add ".csv"
 write.csv(xy.data, filename, quote=FALSE, row.names=FALSE) # write .csv file including x and y coordinates only (will appear in folder specified as working directory)
 # add other fields required for CDpop xy input file;leave values as 'NA' for now
 Subpopulation <- rep(1,nrow(xy.data))
 ID <- c(1:nrow(xy.data))
 sex <- sample(0:1,nrow(xy.data),replace=TRUE)
 # create xy input file
 xy.table <- cbind(Subpopulation, xy.data, ID, sex)
 names(xy.table) <- c("Subpopulation", "XCOORD","YCOORD", "ID", "sex")
 temp2 <- paste("xycdpop_",landscapes[i], sep="") # new file name
 filename2 <- paste(substr(temp2,1,nchar(temp2)-4),".csv", sep="") # remove ".asc" from name and add ".csv"
 write.csv(xy.table, filename2, quote=FALSE, row.names=FALSE) # write .csv file including all necessary fields for CPpop input file (will appear in folder specified as working directory)

 ### Calculate cost distance between each pair of individuals
 landscape <- raster(landscapes[i]) # create raster object from .asc file
 tr <- transition(landscape, transitionFunction = mean, directions = neighbors) # create transition layer
 cdmat <- matrix(NA,nrow=no.individuals, ncol=no.individuals) # preallocate matrix that will hold pairwise cost distances
 for(j in 1:no.individuals) { # loop through pairs of individuals
 for(k in 1:no.individuals) {
 if(j>k) {
 next # # we only want to analyze each pair once
 } else {
 cdmat[j,k] <- costDistance(tr, fromCoords=c(xy.table$XCOORD[j],xy.table$YCOORD[j]), toCoords=c(xy.table$XCOORD[k],xy.table$YCOORD[k])) # calculate cost distance for pair j,k
 cdmat[k,j] <- cdmat[j,k] # assign same value for pair k,j
 }
 }
 }
 temp3 <- paste("cdmat_",landscapes[i], sep="") # new file name
 filename3 <- paste(substr(temp3,1,nchar(temp3)-4),".csv", sep="") # remove ".asc" from name and add ".csv"
 write.matrix(cdmat, filename3, sep=",") # write distance matrix to .csv file (will appear in folder specified as working directory)
}

**Python Code for CDPOP Software**

# CDPOP.py

# Author: Erin L Landguth

# Created: February 2008

# v 1.0 Release: May 2010

#

----------------------------------------------------------------------

------

# Import Modules with Except/Try statements

# CDPOP functions

try:

from CDPOP_Modules import *

from CDPOP_PostProcess import *

from CDPOP_PreProcess import *

except ImportError:

raise ImportError, "CDPOP_Modules required."

# Numpy functions

try:

import numpy as np

except ImportError:

raise ImportError, "Numpy required."

# Python specific functions

import datetime, time,pdb

# ----------------------------------------------------------

# Global symbols, if any :))

#-----------------------------------------------------------

# when set True, routes session log traffic to BOTH the

# screen and to the log file. When False, log traffic just

# sent to log file alone.

msgVerbose = False

#------------------------------------------------------------

# Begin main file execution

#------------------------------------------------------------

if __name__ == '__main__':

#freeze_support() #Windows suppport, function unclear

# General CDPOP information

appName = "CDPOP"

appVers = "version 1.0"

appRele = "2010.11.19-14:09:01EDT"

authorNames = "Erin L Landguth"

# ------------------------------------------------------

# Start timer, get script arguments, create log writeout

# ------------------------------------------------------

# Timing events: start

start_time = datetime.datetime.now()

foldertime = int(time.time())

if len(sys.argv) >= 2:

fileans = sys.argv[1]

# If user did not specify .rip file

else:

print "User must specify input file name (e.g., at

command line type CDPOP.py user_input.csv)."

sys.exit(-1)

# If .ip file does not exist

if not os.path.exists(fileans):

(fileans))

print("Cannot find or open runtime inputs file(%s)"%

sys.exit(-1)

# This properly names log file

logSessionPath = "cdpop.log"

logfHndl =open(logSessionPath,'w')

msgVerbose = True

logMsg(logfHndl,"\n%s Release %s Version %s\n"%

(appName,appRele,appVers))

logMsg(logfHndl,"Author(s): %s"%(authorNames)+'\n')

logMsg(logfHndl,"Session runtime inputs from: %s"%(fileans)

+'\n\n')

msgVerbose = False

# ------------------------------------

# Call DoUserInput()

# ------------------------------------

# Timing events: start

start_time1 = datetime.datetime.now()

# Call function and store inputvarialbes

inputvariables = DoUserInput(fileans)

# Print to log

stringout = 'DoUserInput(): '+str(datetime.datetime.now() -

start_time1) + ''

logMsg(logfHndl,stringout)

print 'DoUserInput(): ',str(datetime.datetime.now() -

start_time1),''

# Timing events: start

start_time1 = datetime.datetime.now()

# -------------------------------------

# Begin Batch Looping

# -------------------------------------

# This loop is defined by the number of rows in

inputvariables.csv

for ibatch in xrange(len(inputvariables)-1):

# Timing events: start

start_timeB = datetime.datetime.now()

# Store all information and the type of each, also do

some error checks

[2]) [3])

xyfilename = str(inputvariables[ibatch+1][0])

agefilename = str(inputvariables[ibatch+1][1])

matecdmatrixfilename = str(inputvariables[ibatch+1]

dispcdmatrixfilename = str(inputvariables[ibatch+1]

mcruns = int(inputvariables[ibatch+1][4])

looptime = int(inputvariables[ibatch+1][5])

nthfile_choice = str(inputvariables[ibatch+1][6])

nthfile_list = inputvariables[ibatch+1][7]

nthfile_seq = inputvariables[ibatch+1][8]

oldmortperc = float(inputvariables[ibatch+1][9])/100

matemoveno = str(inputvariables[ibatch+1][10])

matemoveparA = float(inputvariables[ibatch+1][11])

matemoveparB = float(inputvariables[ibatch+1][12])

matemovethresh = str(inputvariables[ibatch+1][13])

freplace = str(inputvariables[ibatch+1][14])

mreplace = str(inputvariables[ibatch+1][15])

selfans = str(inputvariables[ibatch+1][16])

sexans = str(inputvariables[ibatch+1][17])

reproage = int(str(inputvariables[ibatch+1][18]))

Fdispmoveno = str(inputvariables[ibatch+1][19])

FdispmoveparA = float(inputvariables[ibatch+1][20])

FdispmoveparB = float(inputvariables[ibatch+1][21])

Fdispmovethresh = str(inputvariables[ibatch+1][22])

Mdispmoveno = str(inputvariables[ibatch+1][23])

MdispmoveparA = float(inputvariables[ibatch+1][24])

MdispmoveparB = float(inputvariables[ibatch+1][25])

Mdispmovethresh = str(inputvariables[ibatch+1][26])

offno = str(inputvariables[ibatch+1][27])

lmbda = str(inputvariables[ibatch+1][28])

Femalepercent = int(inputvariables[ibatch+1][29])

equalsexratio = str(inputvariables[ibatch+1][30])

newmortperc = float(inputvariables[ibatch+1][31])/100

Edmatans = str(inputvariables[ibatch+1][32])

gendmatans = str(inputvariables[ibatch+1][33])

gridformat = str(inputvariables[ibatch+1][34])

geneswapgen = int(inputvariables[ibatch+1][35])

muterate = float(inputvariables[ibatch+1][36])

loci = int(inputvariables[ibatch+1][37])

intgenesans = str(inputvariables[ibatch+1][38])

allefreqfilename = str(inputvariables[ibatch+1]

[39]).strip('\n')

alleles = int(inputvariables[ibatch+1]

[40])*np.ones(loci,int)

mtdna = str(inputvariables[ibatch+1][41])

[43])

[44])

[45])

+1][46])

+1][47])

+1][48])

+1][49])

+1][50])

+1][51])

+1][52])

+1][53])

+1][54])

[56])

list or sequence

'sequence':

sequence

cdevolveans = str(inputvariables[ibatch+1][42])

offspringfitsurfaceAA = str(inputvariables[ibatch+1]

offspringfitsurfaceAa = str(inputvariables[ibatch+1]

offspringfitsurfaceaa = str(inputvariables[ibatch+1]

offspringfitsurfaceAABB = str(inputvariables[ibatch

offspringfitsurfaceAaBB = str(inputvariables[ibatch

offspringfitsurfaceaaBB = str(inputvariables[ibatch

offspringfitsurfaceAABb = str(inputvariables[ibatch

offspringfitsurfaceAaBb = str(inputvariables[ibatch

offspringfitsurfaceaaBb = str(inputvariables[ibatch

offspringfitsurfaceAAbb = str(inputvariables[ibatch

offspringfitsurfaceAabb = str(inputvariables[ibatch

offspringfitsurfaceaabb = str(inputvariables[ibatch

cdinfect = str(inputvariables[ibatch+1][55])

transmissionprob = float(inputvariables[ibatch+1]

cdclimate = str(inputvariables[ibatch+1][57])

cdclimgentimelist = inputvariables[ibatch+1][58]

futmatecdmatfilelist = inputvariables[ibatch+1][59]

futdispcdmatfilelist = inputvariables[ibatch+1][60]

# Grab the nthfile list range specific to user input,

if nthfile_choice == 'Sequence' or nthfile_choice ==

# Check if mod == 0, to compute nthfile

if np.mod(looptime,nthfile_seq) == 0:

nthfile = range(0,looptime

+int(nthfile_seq),int(nthfile_seq))

# If mod != 0 then truncate nthfile by one

else:

nthfile = range(0,looptime

+int(nthfile_seq),int(nthfile_seq))

'list':

appending to nthfile

del(nthfile[-1])

if nthfile_choice == 'List' or nthfile_choice ==

nthfile = []

# Split up list, removing space values, and

for inum in

xrange(len(nthfile_list.split('|'))):

# Don't append 0, this gets written

if int(nthfile_list.split('|')

out automatically

[inum]) != 0:

then what user entered, due to the

grid values

nthfile.append(int(nthfile_list.split('|')[inum])-1)

# Error check on nthfile, must be 1 less than

looptime for indexing

if max(nthfile) >= looptime:

print 'nthfile selection maximum value must

be 1 less than your looptime.'

sys.exit(-1)

# Split up cd climate generations by |

if cdclimate == 'Y':

cdclimgentime = []

futmatecdmatfile = []

futdispcdmatfile = []

# Split up list, removing space values, and

appending to nthfile

for inum in

xrange(len(cdclimgentimelist.split('|'))):

# Append individual values to list

cdclimgentime.append(int(cdclimgentimelist.split('|')[inum]))

futmatecdmatfile.append(str(futmatecdmatfilelist.split('|')

[inum].strip('\n')))

futdispcdmatfile.append(str(futdispcdmatfilelist.split('|')

# And then append 1 less # way I have indexed

[inum].strip('\n')))

+1][40]) != 2:

is turned on.'

a long time.

# ---------------------------------

# Some Error checking

# ---------------------------------

# If cdevolve is turned on must have 2 alleles

if cdevolveans != 'N' and int(inputvariables[ibatch

print 'Use 2 alleles per locus when CDEVOLVE

sys.exit(-1)

# ---------------------------------------------

# Begin Monte-Carlo Looping

# ---------------------------------------------

# xrange(mcruns) is typically 10 - 50...and it takes

for ithmcrun in xrange(mcruns):

# Timing events: start

start_timeMC = datetime.datetime.now()

# -----------------------------------------

# Create storage variables

# ------------------------------------------

# These variables will be stored in

output.csv at the end of the simulation

Population = []

Emigrants = []

Deaths = []

Births = []

Immigrants = []

ToTFemales = []

ToTMales = []

BreedFemales = []

BreedMales = []

Alleles = []

He = []

Ho = []

AllelesMutated = []

MateDistED = []

DispDistED = []

MateDistCD = []

DispDistCD = []

MateDistEDstd = []

DispDistEDstd = []

MateDistCDstd = []

DispDistCDstd = []

Infected = []

p1 = []

p2 = []

q1 = []

q2 = []

subpopmigration = []

FAvgMate = []

MAvgMate = []

FSDMate = []

MSDMate = []

# ------------------------------------

# Call DoPreProcess()

# ------------------------------------

# Timing events: start

start_time1 = datetime.datetime.now()

# Prepare fitness surface grid file

if cdevolveans == '1':

offspringfitsurfaceAA =

PrepTextFile(offspringfitsurfaceAA)

offspringfitsurfaceAa =

PrepTextFile(offspringfitsurfaceAa)

offspringfitsurfaceaa =

PrepTextFile(offspringfitsurfaceaa)

# Prepare fitness surface grid file

if cdevolveans == '2':

offspringfitsurfaceAABB =

PrepTextFile(offspringfitsurfaceAABB)

offspringfitsurfaceAaBB =

PrepTextFile(offspringfitsurfaceAaBB)

offspringfitsurfaceaaBB =

PrepTextFile(offspringfitsurfaceaaBB)

offspringfitsurfaceAABb =

PrepTextFile(offspringfitsurfaceAABb)

offspringfitsurfaceAaBb =

PrepTextFile(offspringfitsurfaceAaBb)

offspringfitsurfaceaaBb =

PrepTextFile(offspringfitsurfaceaaBb)

offspringfitsurfaceAAbb =

PrepTextFile(offspringfitsurfaceAAbb)

offspringfitsurfaceAabb =

PrepTextFile(offspringfitsurfaceAabb)

offspringfitsurfaceaabb =

PrepTextFile(offspringfitsurfaceaabb)

# Call function

tupPreProcess =

DoPreProcess(foldertime,ibatch,ithmcrun,\

xyfilename,agefilename,matecdmatrixfilename,dispcdmatrixfilename,\

matemovethresh,Fdispmovethresh,Mdispmovethresh,\

equalsexratio,loci,intgenesans,allefreqfilename,alleles,\

gridformat,

0,logfHndl,cdevolveans,offspringfitsurfaceAA,\

offspringfitsurfaceAa,offspringfitsurfaceaa,cdinfect,Infected,\

offspringfitsurfaceAABB,offspringfitsurfaceAaBB,\

offspringfitsurfaceaaBB,offspringfitsurfaceAABb,\

offspringfitsurfaceAaBb,offspringfitsurfaceaaBb,\

offspringfitsurfaceAAbb,offspringfitsurfaceAabb,\

offspringfitsurfaceaabb,subpopmigration,matemoveno,\

matemoveparA,matemoveparB,Fdispmoveno,FdispmoveparA,\

FdispmoveparB,Mdispmoveno,MdispmoveparA,MdispmoveparB)

ithmcrundir = tupPreProcess[0]

matecdmatrix = tupPreProcess[1]

Fdispcdmatrix = tupPreProcess[2]

Mdispcdmatrix = tupPreProcess[3]

matemovethresh = tupPreProcess[4]

Fdispmovethresh = tupPreProcess[5]

Mdispmovethresh = tupPreProcess[6]

FID = tupPreProcess[7]

id = tupPreProcess[8]

sex = tupPreProcess[9]

age = tupPreProcess[10]

xgrid = tupPreProcess[11]

xgridcopy = copy.deepcopy(xgrid)

ygrid = tupPreProcess[12]

ygridcopy = copy.deepcopy(ygrid)

genes = tupPreProcess[13]

nogrids = tupPreProcess[14]

subpop = tupPreProcess[15]

fitvals1 = tupPreProcess[16]

infection = tupPreProcess[17]

Infected = tupPreProcess[18]

fitvals2 = tupPreProcess[19]

subpopmigration = tupPreProcess[20]

# Print to log

stringout = 'DoPreProcess():

'+str(datetime.datetime.now() -start_time1) + ''

logMsg(logfHndl,stringout)

print 'DoPreProcess():

',str(datetime.datetime.now() -start_time1),''

== cdclimgentime

cdclimgentime

xrange(len(cdclimgentime)):

cdclimgentime[icdtime]:

events: start

= datetime.datetime.now()

----------------------------------------------

cdmatrix.csv for future climate

----------------------------------------------

# -------------------------------------------

# Start Generation Looping

# -------------------------------------------

# Begin generation loop

for gen in xrange(looptime):

# If cdclimate is turned on and gen

if cdclimate == 'Y' and gen:

# Check gen time equal to

for icdtime in

if gen ==

# Timing

start_time1

# # Read in #

# If mate

and disp are the same, then only read in once.

futmatecdmatfile[icdtime] == futdispcdmatfile[icdtime]:

tupReadMat = ReadCDMatrix(futmatecdmatfile[icdtime],matemoveno,\

matemovethresh,matemoveparA,matemoveparB)

Unpack tuple

matecdmatrix = tupReadMat[0]

if

#

Then Set disp = mate

dispcdmatrix = matecdmatrix

and disp are the same, then only read in once.

#

# If mate

if

(futmatecdmatfile[icdtime] == futdispcdmatfile[icdtime]) \

and

(Fdispmoveno == Mdispmoveno) \

(Fdispmovethresh == Mdispmovethresh == matemovethresh):

tupReadMat = ReadCDMatrix(futmatecdmatfile[icdtime],matemoveno,\

matemovethresh,matemoveparA,matemoveparB)

Unpack tuple

matecdmatrix = np.asarray(tupReadMat[0])

Then Set disp = mate

Fdispcdmatrix = matecdmatrix

Mdispcdmatrix = matecdmatrix

#

#

---------------------------------------

Read in cdmatrix.csv - For Mating

---------------------------------------

tupReadMat = ReadCDMatrix(futmatecdmatfile[icdtime],matemoveno,\

matemovethresh,matemoveparA,matemoveparB)

# #

#

matecdmatrix = np.asarray(tupReadMat[0])

and

else: #

--------------------------------------------

Read in cdmatrix.csv - For Female Dispersal

--------------------------------------------

tupReadMat = ReadCDMatrix(futdispcdmatfile[icdtime],Fdispmoveno,\

Fdispmovethresh,FdispmoveparA,FdispmoveparB)

Fdispcdmatrix = np.asarray(tupReadMat[0])

# #

# # #

# Print to

--------------------------------------------

Read in cdmatrix.csv - For Male Dispersal

--------------------------------------------

tupReadMat = ReadCDMatrix(futdispcdmatfile[icdtime],Mdispmoveno,\

Mdispmovethresh,MdispmoveparA,MdispmoveparB)

Mdispcdmatrix = np.asarray(tupReadMat[0])

log

'CDCLIMATE: '+str(datetime.datetime.now() -start_time1) + ''

logMsg(logfHndl,stringout)

'CDCLIMATE: ',str(datetime.datetime.now() -start_time1),'\n'

datetime.datetime.now()

PreProcess step for first

# generation, else use the following updated grid information

datetime.datetime.now()

stringout =

print

# -------------------------------

# Call ReadGrid0()

# -------------------------------

# Use information generated from

# Timing events: start

start_timeGen =

if gen != 0:

# Timing events: start

start_time1 =

ReadGrid(FIDnew,idnew,agenew,xgridnew,\

ygridnew,genesnew,equalsexratio,sexnew,subpopnew,\

infectionnew)

FID = tupReadGrid[0]

sex = tupReadGrid[1]

id = tupReadGrid[2]

age = tupReadGrid[3]

xgrid = tupReadGrid[4]

xgridcopy = tupReadGrid[5]

ygrid = tupReadGrid[6]

ygridcopy = tupReadGrid[7]

genes = tupReadGrid[8]

nogrids = tupReadGrid[9]

subpop = tupReadGrid[10]

infection = tupReadGrid[11]

# Print to log

stringout = 'ReadGrid():

'+str(datetime.datetime.now() -start_time1) + ''

logMsg(logfHndl,stringout)

print 'ReadGrid():

',str(datetime.datetime.now() -start_time1),''

datetime.datetime.now()

tupGetMetrics =

GetMetrics(nogrids,loci,alleles,genes,\

gen,Ho,Alleles,He,subpop,p1,p2,q1,q2)

Ho = tupGetMetrics[0]

Alleles = tupGetMetrics[1]

He = tupGetMetrics[2]

allelefreqlst = tupGetMetrics[3]

p1 = tupGetMetrics[4]

p2 = tupGetMetrics[5]

q1 = tupGetMetrics[6]

q2 = tupGetMetrics[7]

tupReadGrid =

# ---------------------------------

# Call GetMetrics()

# ---------------------------------

# Timing events: start

start_time1 =

# Print to log

stringout = 'GetMetrics():

'+str(datetime.datetime.now() -start_time1) + ''

logMsg(logfHndl,stringout)

print 'GetMetrics():

',str(datetime.datetime.now() -start_time1),''

#

---------------------------------------

# Call DoMate()

#

---------------------------------------

datetime.datetime.now()

# Timing events: start

start_time1 =

tupDoMate =

DoMate(nogrids,sex,reproage,age,\

freplace,mreplace,matemoveno,matemovethresh,\

matecdmatrix,MateDistED,MateDistCD,xgridcopy,\

ygridcopy,ToTMales,ToTFemales,BreedMales,BreedFemales,Population,\

sexans,selfans,FID,matemoveparA,matemoveparB,\

MateDistCDstd,FAvgMate,MAvgMate,\

MateDistEDstd,

FSDMate,MSDMate)

ToTMales = tupDoMate[0]

ToTFemales = tupDoMate[1]

BreedMales = tupDoMate[2]

BreedFemales = tupDoMate[3]

Population = tupDoMate[4]

Bearpairs = tupDoMate[5]

CDpairs = tupDoMate[5]

MateDistED = tupDoMate[6]

MateDistCD = tupDoMate[7]

MateDistEDstd = tupDoMate[8]

MateDistCDstd = tupDoMate[9]

FAvgMate = tupDoMate[10]

MavgMate = tupDoMate[11]

FSDMate = tupDoMate[12]

MSDMate = tupDoMate[13]

# Print to log

stringout = 'DoMate():

'+str(datetime.datetime.now() -start_time1) + ''

logMsg(logfHndl,stringout)

print 'DoMate():

',str(datetime.datetime.now() -start_time1),''

#

---------------------------------------

# Call DoOffspring()

#

---------------------------------------

# Timing events: start

start_time1 =

datetime.datetime.now()

tupDoOff =

DoOffspring(offno,lmbda,Bearpairs,CDpairs,Femalepercent,\

Births,genes,infection,transmissionprob)

offspring = tupDoOff[0]

offspringno = tupDoOff[1]

# Print to log

stringout = 'DoOffspring():

'+str(datetime.datetime.now() -start_time1) + ''

logMsg(logfHndl,stringout)

print 'DoOffspring():

',str(datetime.datetime.now() -start_time1),''

#

---------------------------------------

# Call InheritGenes()

#

---------------------------------------

# Timing events: start

start_time1 =

datetime.datetime.now()

tupDoGenes =

InheritGenes(gen,geneswapgen,AllelesMutated,offspringno,\

offspring,genes,loci,muterate,mtdna)

offspring = tupDoGenes[0]

AllelesMutated = tupDoGenes[1]

# Print to log

stringout = 'InheritGenes():

'+str(datetime.datetime.now() -start_time1) + ''

logMsg(logfHndl,stringout)

print 'InheritGenes():

',str(datetime.datetime.now() -start_time1),''

#

------------------------------------------

# Call DoAdultMortality()

#

------------------------------------------

# Timing events: start

start_time1 =

datetime.datetime.now()

tupAMort =

DoAdultMortality(nogrids,oldmortperc,sex,id,\

age,xgrid,ygrid,gen,geneswapgen,genes,FID,Deaths)

freegrid = tupAMort[0]

Deaths = tupAMort[1]

FID = tupAMort[2]

id = tupAMort[3]

sex = tupAMort[4]

age = tupAMort[5]

xgrid = tupAMort[6]

ygrid = tupAMort[7]

genes = tupAMort[8]

oldmort = tupAMort[9]

# Print to log

stringout = 'DoAdultMortality():

'+str(datetime.datetime.now() -start_time1) + ''

logMsg(logfHndl,stringout)

print 'DoAdultMortality():

',str(datetime.datetime.now() -start_time1),''

#

------------------------------------------

# Call DoDisperse()

#

------------------------------------------

datetime.datetime.now()

# Timing events: start

start_time1 =

tupDoDisp =

DoDisperse(offspringno,freegrid,offspring,Fdispmoveno,\

Mdispmoveno,Fdispcdmatrix,Mdispcdmatrix,Fdispmovethresh,Mdispmovethres

h,gen,\

Emigrants,Immigrants,loci,alleles,nogrids,geneswapgen,\

xgridcopy,ygridcopy,DispDistED,DispDistCD,allelefreqlst,\

logfHndl,cdevolveans,fitvals1,newmortperc,FdispmoveparA,\

FdispmoveparB,MdispmoveparA,MdispmoveparB,fitvals2,DispDistEDstd,\

DispDistCDstd,subpop,subpopmigration)

OffDisperseIN = tupDoDisp[0]

Newimmigrants = tupDoDisp[1]

Emigrants = tupDoDisp[2]

Immigrants = tupDoDisp[3]

DispDistED = tupDoDisp[4]

DispDistCD = tupDoDisp[5]

DispDistEDstd = tupDoDisp[6]

DispDistCDstd = tupDoDisp[7]

# Print to log

stringout = 'DoDisperse():

'+str(datetime.datetime.now() -start_time1) + ''

logMsg(logfHndl,stringout)

print 'DoDisperse():

',str(datetime.datetime.now() -start_time1),''

#

------------------------------------------

# Call DoOutput()

#

------------------------------------------

# Timing events: start

start_time1 =

datetime.datetime.now()

tupDoOut =

DoOutput(nogrids,FID,OffDisperseIN,Newimmigrants,\

xgridcopy,ygridcopy,gen,geneswapgen,id,sex,age,xgrid,\

ygrid,genes,nthfile,ithmcrundir,loci,alleles,subpop,\

logfHndl,gridformat,intgenesans,infection,Infected,cdinfect)

FIDnew = tupDoOut[0]

idnew = tupDoOut[1]

sexnew = tupDoOut[2]

agenew = tupDoOut[3]

xgridnew = tupDoOut[4]

ygridnew = tupDoOut[5]

genesnew = tupDoOut[6]

subpopnew = tupDoOut[7]

infectionnew = tupDoOut[8]

# Print to log

stringout = 'DoOutput():

'+str(datetime.datetime.now() -start_time1) + ''

logMsg(logfHndl,stringout)

print 'DoOutput():

',str(datetime.datetime.now() -start_time1),''

# Print to log

stringout = 'End Generation

Loop'+str(gen)+': '+str(datetime.datetime.now() -start_timeGen) + '\n'

logMsg(logfHndl,stringout)

print 'End Generation

Loop',str(gen),': ',str(datetime.datetime.now() -start_timeGen),'\n'

# End::generation loop

# ------------------------------------------

# Call DoPostProcess()

# ------------------------------------------

# Timing events: start

start_time1 = datetime.datetime.now()

DoPostProcess(ithmcrundir,nogrids,\

matecdmatrix,Fdispcdmatrix,Mdispcdmatrix,Edmatans,xgridcopy,ygridcopy,

gendmatans,\

loci,alleles,looptime,Population,ToTFemales,ToTMales,\

BreedFemales,BreedMales,Emigrants,Immigrants,Births,\

Deaths,Alleles,He,Ho,AllelesMutated,\

MateDistED,DispDistED,MateDistCD,DispDistCD,nthfile,\

gen,logfHndl,p1,p2,q1,q2,Infected,subpop,MateDistEDstd,\

**Python Code for Calculating Genetic Distance Matrices**

 # v5.0 - 2012April5 -- Will calculate on specified generations.

# v4.0 - 2012March29 -- Will list folders in directory. Will not # work for NA.

# v3.0 - 2011June27 -- Assumes files in grid*.csv, but for new version of # CDPOP v1.0. Just Dps.

 # v2.0 - 2008Dec22 -- Assumes files are in the gridNNN format, but reads in

 # *.csv, not grid*.csv

 # v1.0 - March 2008 -- Assumes files are named grid*.csv

 # GeneticDistance.py

 # Author: Erin L Landguth

# Created: March 2008

# Description: This program calculates a genetic distance matrix using:

- #  Bray-Curtis Method:
- #  formula 1 - 2W/(A+B), where W is the minimum value between the two comp-  arison's A and B. The specific application is to calculate this distance # matrix for genotypes of a population with n individuals: Genetic Distance.
- #  Proportion of Shared Alleles:
- #  Nei's:
- #  1 - sqrt(ithfreq*jthfreq)/loci
- #  Proportion of Shared alleles:
- #  1 - proportion of shared alleles between individuals. # Program Input: directory of *.csv files # Program Output: oldname+Gdmatrix.csv # Program Steps:
- #  1. User input information.
- #  2. fileList all of the *.csv in directory
- #  3. Run genetic distance method # ---------------------------------------------------------------------- ------

import glob

The power of glob

from numpy import *

commands and functions

from numpy.random import *

calculations

import time, datetime,os,pdb

# Timing events: start

start_time = datetime.datetime.now()

# # General

# Random/statistic

# Other libraries

# ------------------------------------------

# Step 1: Get user information

# ------------------------------------------

# Store directory path name

directory = 'C:\\CDPOP\\'

# Number of loci

loci = 30

# Number of alleles per locus

noalleperlocus = 30

alleles = int(noalleperlocus)*ones(loci,int)

# If variable alleles per locus

#alleles = array([6,10])

# The number of individuals

nogrids = 1000

# The generations to run

gen =

[0,1,2,3,4,5,10,25,50,75,100,150,200,225,250,275,300,325,350,400,450,5

00,550,600,650,700,750,800,850,900,950,1000]

# -------------------------------

# Step 2: List files in directory

# -------------------------------

# List folders in this dir

def listdirs(folder):

return [d for d in (os.path.join(folder, d1) for d1 in

os.listdir(folder)) if os.path.isdir(d)]

folderList = listdirs(directory)

# Loop through folderList

for ifold in xrange(len(folderList)):

# -----------------------------------

# Step 3: Run genetic distance method

# -----------------------------------

# ------------ Genetic Distance Matrix: Proportion of shared

alleles -----------------------

# List all files with .csv extensions (but only the grid ones)

print '\n'

print 'Creating the proportion of shared alleles genetic

distance matrices...'

# Get the first globbed file read in

for i in xrange(len(gen)):

# Open file for reading

inputfile = open(folderList[ifold]+'\

\grid'+str(gen[i])+'.csv','r')

matrix, x

# Read lines from the file

lines = inputfile.readlines()

#Close the file

inputfile.close()

# Create an empty matrix to append to

x = []

# Split up each line in file and append to empty

for l in lines:

thisline = l.split(',')

x.append(thisline)

# Store genetic information: genes[bear], but need

them as float values

genes = []

tempgenes = []

for k in range(len(x)-1):

[7:int(7+sum(alleles))])

[j]))

inidividual j

written over for each comparison

# Get list from read in file

tempgenes.append(x[k+1]

# Create spot in genes

genes.append([])

for j in range(sum(alleles)):

# Make each list spot an integer

genes[k].append(float(tempgenes[k]

# Create a matrix of zeros to be filled

gendmatrix = zeros((nogrids,nogrids),float)

# Loop through each individual k

for k in range(nogrids):

# Compare individual k to every other

for j in range(nogrids):

# Create a tempvariable to be

tempmin=[]

# Loop through each allele value

for alle in range(sum(alleles)):

between k and j checking the 4 conditions

[alle]==2.0:

tempmin.append(2)

[alle]==1.0:

tempmin.append(1)

[alle]==1.0:

tempmin.append(1)

[alle]==2.0:

tempmin.append(1)

float(sum(tempmin))/(2*loci)

# Strip directory/filename of grid and add

'Gdmatrix.csv'

\Gdmatrix'+str(gen[i])+'.csv'

element to outputfile

# Grab each element in each row and write

for ele in range(len(seqrow)):

outputfile.write(str(seqrow[ele]))

# Add comma

outputfile.write(',')

# Return line

outputfile.write('\n')

gdpathname = folderList[ifold]+'\

# Create file to write matrix to

outputfile = open(gdpathname,'w')

# Sequence each row in the matrix

for seqrow in gendmatrix:

# Find the shared alleles

if genes[k][alle]==2.0:

if genes[j]

elif genes[j]

elif genes[k][alle]==1.0:

if genes[j]

elif genes[j]

# Write the Dps value to gendmatrix

gendmatrix[k][j] = 1-

# Close file

outputfile.close()

print '\n'

print 'The genetic distance matrix '+gdpathname+' has

been created in '+str(datetime.datetime.now() -start_time)

DispDistEDstd,MateDistCDstd,DispDistCDstd,subpopmigration,\

FAvgMate,MAvgMate,FSDMate,MSDMate)

# Print to log

stringout = 'DoPostProcess():

'+str(datetime.datetime.now() -start_time1) + ''

logMsg(logfHndl,stringout)

print 'DoPostProcess():

',str(datetime.datetime.now() -start_time1),''

# Print to log

stringout = 'End Monte Carlo

Loop'+str(ithmcrun)+': '+str(datetime.datetime.now() -start_timeMC) +

'\n'

logMsg(logfHndl,stringout)

print 'End Monte Carlo Loop',str(ithmcrun),':

',str(datetime.datetime.now() -start_timeMC),'\n'

# End::Monte Carlo Loop

# Print to log

stringout = 'End Batch Loop'+str(ibatch)+':

'+str(datetime.datetime.now() -start_timeB) + '\n'

logMsg(logfHndl,stringout)

print 'End Batch Loop',str(ibatch),':

',str(datetime.datetime.now() -start_timeB),'\n'

#End::Batch Loop

# End::Main Loop

# Print to log

stringout = 'Total CDPOP Simulation Time:

'+str(datetime.datetime.now() -start_time) + ''

logMsg(logfHndl,stringout)

logfHndl.close()

print 'Total CDPOP Simulation Time: ',str(datetime.datetime.now() -

start_time),''

# R code for mantel.mc function that runs Mantel tests

v MultipleLandscapes: 05.25.2012 v Animal Movement Group Functions: 09.23.2010 v 5 - Simulation Group Project Functions: 03.13.2013 Erin L. Landguth MRMCFun_MultipleLandscapes.R
 Project Description: 1. mantel.mc: Function to run simple and partial mantel test on distance, barrier, and genetic matrices extracting information from mutliple folders that were created through a batch or Monte Carlo process. Results outputted to MRMCgentoXXXXXX.csv - the corresponding Mantel test. 2. mantel.mc.analysis: Function to read in MRMCgentoXXX.csv, calculate mean, sd, confidence intervals and plot these results. Libraries Needed: 1. Spatial 2. Ecodist Project Input: 1. Function parameter inputs...

Project Date: December 10, 2008

Function code for Mantel tests

mantel.mc <- function(batchno,mcrunno,N,nthfile,gddir,gdfilename,barrdir,barrfilename,barrans,
 distdir,distfilename,distans,landdir,landfilename,landans,samplestyle,sampleno,sampledir,gentodist.ans,
 gentobarr.ans,gentoland.ans,gentodist.barr.ans,gentodist.land.ans,gentobarr.dist.ans,gentobarr.land.ans,
 gentoland.dist.ans,gentoland.barr.ans,gentoland.land.ans,mperms,mgram.gentodist.ans,mgram.gentobarr.ans,mgram.gentoland.ans,
 mgram.gentodist.barr.ans,mgram.gentodist.land.ans,mgram.gentobarr.dist.ans,mgram.gentobarr.land.ans,
 mgram.gentoland.dist.ans,mgram.gentoland.barr.ans,mgram.gentoland.land.ans,mgramruntime,batchstring,logans,mrank,
 nboot, pboot, cboot,mcstart,iptstart)
{
 ################################
 ## 1. Read in sample information
 ################################
 # Random draw
 if (samplestyle == 'random')
 {
 sampledraw <- runif(sampleno,1,N)
 }
 # Known points draw
 if (samplestyle == 'known')
 {
 sampledraw <- read.table(paste(sampledir,sep=""),sep=",",header=TRUE)
 sampledraw <- sampledraw$SelectedID
 }
 # All points draw
 if (samplestyle == 'all')
 {
 sampledraw <- seq(1,N)
 }

 # For AIC calculations
 n <- N*(N-1)/2

 ######################
 ## 2. Batch Loop Begin
 ######################
 for (i in 1:batchno)
 {
 # Create batch directory string
 batchfiledir <- paste(batchstring,'batchrun',as.character(i-1),sep="")
 print(batchfiledir)

 ##########################
 ## 3. Loop through points
 ##########################
 for (ipt in iptstart:length(landfilename))
 {

 ###########################################
 ## 3. Read in Data - Cost Distance Matrices
 ###########################################
 # Read in barrier matrix
 if (barrans == 'Y')
 {
 if (length(barrfilename) == 1)
 {
 barrier <- read.table(paste(barrdir,barrfilename[1],sep=""),sep=",",header=FALSE)
 }
 if (length(barrfilename) != 1)
 {
 barrier <- read.table(paste(barrdir,barrfilename[1],sep=""),sep=",",header=FALSE)
 }
 barriermat <- as.matrix(barrier[,1:N])
 barrier <- lower(barriermat[sampledraw,sampledraw])
 # If log transformed turned on
 if(logans == 'Y')
 {
 barriermat <- log(barriermat)
 barrier <- log(barrier)
 }
 }
 # Read in distance matrix
 if (distans == 'Y')
 {
 if (length(distfilename) == 1)
 {
 distance <- read.table(paste(distdir,distfilename[1],sep=""),sep=",",header=FALSE)
 }
 if (length(distfilename) != 1)
 {
 distance <- read.table(paste(distdir,distfilename[ipt],sep=""),sep=",",header=FALSE)
 }
 distancemat <- as.matrix(distance[,1:N])
 distance <- lower(distancemat[sampledraw,sampledraw])
 # If log transformed turned on
 if(logans == 'Y')
 {
 distancemat <- log(distancemat)
 distance <- log(distance)
 }

 }
 # Read in landscape matrix
 if (landans == 'Y')
 {
 if (length(landfilename) == 1)
 {
 landscape1 <- read.table(paste(landdir,landfilename[1],sep=""),sep=",",header=FALSE)
 }
 if (length(landfilename) != 1)
 {
 landscape1 <- read.table(paste(landdir,landfilename[ipt],sep=""),sep=",",header=FALSE)
 }
 landscape1mat <- as.matrix(landscape1[,1:N])
 landscape1 <- lower(landscape1mat[sampledraw,sampledraw])
 # If log transformed turned on
 if(logans == 'Y')
 {
 landscape1mat <- log(landscape1mat)
 landscape1 <- log(landscape1)
 }

 }

 ##########################
 ## 4. Landscape2 Loop Begin
 ##########################
 if(gentoland.land.ans == 'Y')
 {
 land2loop <- length(landfilename)
 }
 if(gentoland.land.ans == 'N')
 {
 land2loop <- 1
 }

 for (iland in 1:land2loop)
 {
 # Read in additional landscape matrix
 if (gentoland.land.ans == 'Y')
 {
 if (length(landfilename) == 1)
 {
 landscape2 <- read.table(paste(landdir,landfilename[1],sep=""),sep=",",header=FALSE)
 }
 if (length(landfilename) != 1)
 {
 landscape2 <- read.table(paste(landdir,landfilename[iland],sep=""),sep=",",header=FALSE)
 }
 landscape2mat <- as.matrix(landscape2[,1:N])
 landscape2 <- lower(landscape2mat[sampledraw,sampledraw])
 # If log transformed turned on
 if(logans == 'Y')
 {
 landscape2mat <- log(landscape2mat)
 landscape2 <- log(landscape2)
 }

 # Create landscape that on string
 #landfileon <- paste('L1',landfilename[ipt],'_L2',landfilename[iland],'_LOG',logans,'_MRANK',as.character(mrank),sep="")
 landfileon <- paste('L1',strsplit(strsplit(landfilename[ipt],"_")[[1]][1],".txt")[[1]][1],'_L2',strsplit(strsplit(landfilename[iland],"_")[[1]][1],".txt")[[1]][1],sep="")
 print(landfileon)
 }
 # Read in additional landscape matrix
 if (gentoland.land.ans == 'N')
 {
 # Create landscape that on string
 landfileon <- paste('L1',strsplit(strsplit(landfilename[ipt],"_")[[1]][1],".txt")[[1]][1],sep="")
 print(landfileon)
 }

 ############################
 ## 5. Monte Carlo Loop Begin
 ############################
 for (j in mcstart:mcrunno)
 {

 # Create Monte Carlo directory string
 mcfiledir <- paste('mcrun',as.character(j-1),'/',sep="")
 print(mcfiledir)

 #####################################
 ## 6. Preliminary vector storage work
 #####################################
 # Create empty vectors to append to for mantelr,pval1,llim,ulim: check all cases
 # Simple genetic ~ distance
 if (gentodist.ans=='Y')
 {
 gentodist.mr <- c()
 gentodist.pv1 <- c()
 gentodist.pv2 <- c()
 gentodist.pv3 <- c()
 gentodist.llim <- c()
 gentodist.ulim <- c()
 gentodist.aic <- c()
 gentodist.aicd <- c()
 k.dist <- 1
 }
 # Simple genetic ~ barrier
 if (gentobarr.ans=='Y')
 {
 gentobarr.mr <- c()
 gentobarr.pv1 <- c()
 gentobarr.pv2 <- c()
 gentobarr.pv3 <- c()
 gentobarr.llim <- c()
 gentobarr.ulim <- c()
 }
 # Simple genetic ~ landscape
 if (gentoland.ans=='Y')
 {
 gentoland.mr <- c()
 gentoland.pv1 <- c()
 gentoland.pv2 <- c()
 gentoland.pv3 <- c()
 gentoland.llim <- c()
 gentoland.ulim <- c()
 gentoland.aic <- c()
 gentoland.aicd <- c()
 k.land <- 1
 }
 # Partial genetic ~ distance|barrier
 if (gentodist.barr.ans=='Y')
 {
 gentodist.barr.mr <- c()
 gentodist.barr.pv1 <- c()
 gentodist.barr.pv2 <- c()
 gentodist.barr.pv3 <- c()
 gentodist.barr.llim <- c()
 gentodist.barr.ulim <- c()
 }
 # Partial genetic ~ distance|landscape
 if (gentodist.land.ans=='Y')
 {
 gentodist.land.mr <- c()
 gentodist.land.pv1 <- c()
 gentodist.land.pv2 <- c()
 gentodist.land.pv3 <- c()
 gentodist.land.llim <- c()
 gentodist.land.ulim <- c()
 gentodist.land.aic <- c()
 gentodist.land.aicd <- c()
 k.dist.land <- 2
 }
 # Partial genetic ~ barrier|landscape
 if (gentobarr.land.ans=='Y')
 {
 gentobarr.land.mr <- c()
 gentobarr.land.pv1 <- c()
 gentobarr.land.pv2 <- c()
 gentobarr.land.pv3 <- c()
 gentobarr.land.llim <- c()
 gentobarr.land.ulim <- c()
 }
 # Partial genetic ~ barrier|distance
 if (gentobarr.dist.ans=='Y')
 {
 gentobarr.dist.mr <- c()
 gentobarr.dist.pv1 <- c()
 gentobarr.dist.pv2 <- c()
 gentobarr.dist.pv3 <- c()
 gentobarr.dist.llim <- c()
 gentobarr.dist.ulim <- c()
 }
 # Partial genetic ~ landscape|distance
 if (gentoland.dist.ans=='Y')
 {
 gentoland.dist.mr <- c()
 gentoland.dist.pv1 <- c()
 gentoland.dist.pv2 <- c()
 gentoland.dist.pv3 <- c()
 gentoland.dist.llim <- c()
 gentoland.dist.ulim <- c()
 gentoland.dist.aic <- c()
 gentoland.dist.aicd <- c()
 k.land.dist <- 2
 }
 # Partial genetic ~ landscape|barrier
 if (gentoland.barr.ans=='Y')
 {
 gentoland.barr.mr <- c()
 gentoland.barr.pv1 <- c()
 gentoland.barr.pv2 <- c()
 gentoland.barr.pv3 <- c()
 gentoland.barr.llim <- c()
 gentoland.barr.ulim <- c()
 }

 # Partial genetic ~ landscape|landscape
 if (gentoland.land.ans=='Y')
 {
 gentoland.land.mr <- c()
 gentoland.land.pv1 <- c()
 gentoland.land.pv2 <- c()
 gentoland.land.pv3 <- c()
 gentoland.land.llim <- c()
 gentoland.land.ulim <- c()
 gentoland.land.aic <- c()
 gentoland.land.aicd <- c()
 k.land.land <- 2
 }

 ###################################################################
 ## 6. Mantel Test:
 ## Simple: genetic ~ distance
 ## Simple: genetic ~ barrier
 ## Simple: genetic ~ barrier
 ## Partial: genetic ~ distance|barrier
 ## Partial: genetic ~ barrier|distance
 ## Partial: genetic ~ distance|distance
 ## Partial: genetic ~ landscape|landscape
 ## Mantel Correlogram for each of the above or specified.
 ###################################################################

 # Start for loop through each Gdmatrix
 for (k in 1:length(nthfile))
 {
 # Read in genetic distance matrix - piece it up for character read
 file1 <- as.character(nthfile[k]) # For specified nthfile
 file2 <- ".csv"
 genetic <- read.table(paste(gddir,batchfiledir,mcfiledir,gdfilename,file1,file2,sep=""),sep=",",header=FALSE)
 geneticmat <- as.matrix(genetic[,1:N])
 print(nthfile[k])

 # Make matrices lower
 genetic <- lower(geneticmat[sampledraw,sampledraw])

 # Run Mantel appending results to empty vector: distance
 if (gentodist.ans == 'Y')
 {
 mantelrun <- mantel(genetic~distance, nperm=mperms, mrank=mrank, nboot=nboot, pboot=pboot,cboot=cboot)
 # Append loop information
 gentodist.mr <- append(gentodist.mr,mantelrun[1])
 gentodist.pv1 <- append(gentodist.pv1,mantelrun[2])
 gentodist.pv2 <- append(gentodist.pv2,mantelrun[3])
 gentodist.pv3 <- append(gentodist.pv3,mantelrun[4])
 gentodist.llim <- append(gentodist.llim,mantelrun[5])
 gentodist.ulim <- append(gentodist.ulim,mantelrun[6])
 # Check for correlogram
 if (mgram.gentodist.ans == 'Y')
 {
 if (nthfile[k] == mgramruntime)
 {
 gentodist.mgram <- mgram(genetic,distance,nperm=mperms, mrank=mrank)
 }
 }
 } # End gentodist

 # Run Mantel appending results to empty vector: barrier
 if (gentobarr.ans == 'Y')
 {
 mantelrun <- mantel(genetic~barrier, nperm=mperms, mrank=mrank, nboot=nboot, pboot=pboot,cboot=cboot)
 # Append loop information
 gentobarr.mr <- append(gentobarr.mr,mantelrun[1])
 gentobarr.pv1 <- append(gentobarr.pv1,mantelrun[2])
 gentobarr.pv2 <- append(gentobarr.pv2,mantelrun[3])
 gentobarr.pv3 <- append(gentobarr.pv3,mantelrun[4])
 gentobarr.llim <- append(gentobarr.llim,mantelrun[5])
 gentobarr.ulim <- append(gentobarr.ulim,mantelrun[6])
 # Check for correlogram
 if (mgram.gentobarr.ans == 'Y')
 {
 if (nthfile[k] == mgramruntime)
 {
 gentobarr.mgram <- mgram(genetic,barrier,nperm=mperms,mrank=mrank)
 }
 }
 }

 # Run Mantel appending results to empty vector: landscape
 if (gentoland.ans == 'Y')
 {
 mantelrun <- mantel(genetic~landscape1, nperm=mperms, mrank=mrank, nboot=nboot, pboot=pboot,cboot=cboot)
 # Append loop information
 gentoland.mr <- append(gentoland.mr,mantelrun[1])
 gentoland.pv1 <- append(gentoland.pv1,mantelrun[2])
 gentoland.pv2 <- append(gentoland.pv2,mantelrun[3])
 gentoland.pv3 <- append(gentoland.pv3,mantelrun[4])
 gentoland.llim <- append(gentoland.llim,mantelrun[5])
 gentoland.ulim <- append(gentoland.ulim,mantelrun[6])
 # Check for correlogram
 if (mgram.gentoland.ans == 'Y')
 {
 if (nthfile[k] == mgramruntime)
 {
 gentoland.mgram <- mgram(genetic,landscape1,nperm=mperms,mrank=mrank)
 }
 }
 }

 # Run Mantel appending results to empty vector: distance|barrier
 if (gentodist.barr.ans == 'Y')
 {
 mantelrun <- mantel(genetic~distance+barrier, nperm=mperms, mrank=mrank, nboot=nboot, pboot=pboot,cboot=cboot)
 # Append loop information
 gentodist.barr.mr <- append(gentodist.barr.mr,mantelrun[1])
 gentodist.barr.pv1 <- append(gentodist.barr.pv1,mantelrun[2])
 gentodist.barr.pv2 <- append(gentodist.barr.pv2,mantelrun[3])
 gentodist.barr.pv3 <- append(gentodist.barr.pv3,mantelrun[4])
 gentodist.barr.llim <- append(gentodist.barr.llim,mantelrun[5])
 gentodist.barr.ulim <- append(gentodist.barr.ulim,mantelrun[6])
 # Check for correlogram
 if (mgram.gentodist.barr.ans == 'Y')
 {
 if (nthfile[k] == mgramruntime)
 {
 gentodist.barr.mgram <- mgram(genetic,distance+barrier,nperm=mperms,mrank=mrank)
 }
 }
 }

 # Run Mantel appending results to empty vector: distance|landscape
 if (gentodist.land.ans == 'Y')
 {
 mantelrun <- mantel(genetic~distance+landscape1, nperm=mperms, mrank=mrank, nboot=nboot, pboot=pboot,cboot=cboot)
 # Append loop information
 gentodist.land.mr <- append(gentodist.land.mr,mantelrun[1])
 gentodist.land.pv1 <- append(gentodist.land.pv1,mantelrun[2])
 gentodist.land.pv2 <- append(gentodist.land.pv2,mantelrun[3])
 gentodist.land.pv3 <- append(gentodist.land.pv3,mantelrun[4])
 gentodist.land.llim <- append(gentodist.land.llim,mantelrun[5])
 gentodist.land.ulim <- append(gentodist.land.ulim,mantelrun[6])
 # Check for correlogram
 if (mgram.gentodist.land.ans == 'Y')
 {
 if (nthfile[k] == mgramruntime)
 {
 gentodist.land.mgram <- mgram(genetic,distance+landscape1,nperm=mperms,mrank=mrank)
 }
 }
 }

 # Run Mantel appending results to empty vector: barrier|distance
 if (gentobarr.dist.ans == 'Y')
 {
 mantelrun <- mantel(genetic~barrier+distance, nperm=mperms, mrank=mrank, nboot=nboot, pboot=pboot,cboot=cboot)
 # Append loop information
 gentobarr.dist.mr <- append(gentobarr.dist.mr,mantelrun[1])
 gentobarr.dist.pv1 <- append(gentobarr.dist.pv1,mantelrun[2])
 gentobarr.dist.pv2 <- append(gentobarr.dist.pv2,mantelrun[3])
 gentobarr.dist.pv3 <- append(gentobarr.dist.pv3,mantelrun[4])
 gentobarr.dist.llim <- append(gentobarr.dist.llim,mantelrun[5])
 gentobarr.dist.ulim <- append(gentobarr.dist.ulim,mantelrun[6])
 # Check for correlogram
 if (mgram.gentobarr.dist.ans == 'Y')
 {
 if (nthfile[k] == mgramruntime)
 {
 gentobarr.dist.mgram <- mgram(genetic,barrier+distance,nperm=mperms,mrank=mrank)
 }
 }
 }

 # Run Mantel appending results to empty vector: barrier|landscape
 if (gentobarr.land.ans == 'Y')
 {
 mantelrun <- mantel(genetic~barrier+landscape1, nperm=mperms, mrank=mrank, nboot=nboot, pboot=pboot,cboot=cboot)
 # Append loop information
 gentobarr.land.mr <- append(gentobarr.land.mr,mantelrun[1])
 gentobarr.land.pv1 <- append(gentobarr.land.pv1,mantelrun[2])
 gentobarr.land.pv2 <- append(gentobarr.land.pv2,mantelrun[3])
 gentobarr.land.pv3 <- append(gentobarr.land.pv3,mantelrun[4])
 gentobarr.land.llim <- append(gentobarr.land.llim,mantelrun[5])
 gentobarr.land.ulim <- append(gentobarr.land.ulim,mantelrun[6])
 # Check for correlogram
 if (mgram.gentobarr.land.ans == 'Y')
 {
 if (nthfile[k] == mgramruntime)
 {
 gentobarr.land.mgram <- mgram(genetic,barrier+landscape1,nperm=mperms,mrank=mrank)
 }
 }
 }

 # Run Mantel appending results to empty vector: landscape|distance
 if (gentoland.dist.ans == 'Y')
 {
 mantelrun <- mantel(genetic~landscape1+distance, nperm=mperms, mrank=mrank, nboot=nboot, pboot=pboot,cboot=cboot)
 # Append loop information
 gentoland.dist.mr <- append(gentoland.dist.mr,mantelrun[1])
 gentoland.dist.pv1 <- append(gentoland.dist.pv1,mantelrun[2])
 gentoland.dist.pv2 <- append(gentoland.dist.pv2,mantelrun[3])
 gentoland.dist.pv3 <- append(gentoland.dist.pv3,mantelrun[4])
 gentoland.dist.llim <- append(gentoland.dist.llim,mantelrun[5])
 gentoland.dist.ulim <- append(gentoland.dist.ulim,mantelrun[6])
 # Check for correlogram
 if (mgram.gentoland.dist.ans == 'Y')
 {
 if (nthfile[k] == mgramruntime)
 {
 gentoland.dist.mgram <- mgram(genetic,landscape1+distance,nperm=mperms,mrank=mrank)
 }
 }
 }

 # Run Mantel appending results to empty vector: landscape|barrier
 if (gentoland.barr.ans == 'Y')
 {
 mantelrun <- mantel(genetic~landscape1+barrier, nperm=mperms, mrank=mrank, nboot=nboot, pboot=pboot,cboot=cboot)
 # Append loop information
 gentoland.barr.mr <- append(gentoland.barr.mr,mantelrun[1])
 gentoland.barr.pv1 <- append(gentoland.barr.pv1,mantelrun[2])
 gentoland.barr.pv2 <- append(gentoland.barr.pv2,mantelrun[3])
 gentoland.barr.pv3 <- append(gentoland.barr.pv3,mantelrun[4])
 gentoland.barr.llim <- append(gentoland.barr.llim,mantelrun[5])
 gentoland.barr.ulim <- append(gentoland.barr.ulim,mantelrun[6])
 # Check for correlogram
 if (mgram.gentoland.barr.ans == 'Y')
 {
 if (nthfile[k] == mgramruntime)
 {
 gentoland.barr.mgram <- mgram(genetic,landscape1+barrier,nperm=mperms,mrank=mrank)
 }
 }

 }

 # Run Mantel appending results to empty vector: landscape|landscape
 if (gentoland.land.ans == 'Y')
 {
 mantelrun <- mantel(genetic~landscape1+landscape2, nperm=mperms, mrank=, nboot=nboot, pboot=pboot,cboot=cboot)
 # Append loop information
 gentoland.land.mr <- append(gentoland.land.mr,mantelrun[1])
 gentoland.land.pv1 <- append(gentoland.land.pv1,mantelrun[2])
 gentoland.land.pv2 <- append(gentoland.land.pv2,mantelrun[3])
 gentoland.land.pv3 <- append(gentoland.land.pv3,mantelrun[4])
 gentoland.land.llim <- append(gentoland.land.llim,mantelrun[5])
 gentoland.land.ulim <- append(gentoland.land.ulim,mantelrun[6])
 # Check for correlogram
 if (mgram.gentoland.land.ans == 'Y')
 {
 if (nthfile[k] == mgramruntime)
 {
 gentoland.land.mgram <- mgram(genetic,landscape1+landscape2,nperm=mperms,mrank=mrank)
 }
 }

 }# Last Mantel Run End

 }# Mantel Loop End

 #######################################
 ## 7. Output mantel information to file
 #######################################
 ## Transpose and data.frame vectors and then print and write to file
 if (gentodist.ans=='Y')
 {
 gentodist.mr <- t(data.frame(gentodist.mr))
 gentodist.pv1 <- t(data.frame(gentodist.pv1))
 gentodist.pv2 <- t(data.frame(gentodist.pv2))
 gentodist.pv3 <- t(data.frame(gentodist.pv3))
 gentodist.llim <- t(data.frame(gentodist.llim))
 gentodist.ulim <- t(data.frame(gentodist.ulim))
 # File name
 fileoutputname <- paste(landfileon,"MRMCgentodist.csv",sep="")
 write.table(paste(batchfiledir,mcfiledir,landfileon,sep=""),file=paste(gddir,fileoutputname,sep=""),append=TRUE,sep=",",eol=",",
 row.names=FALSE,col.names=FALSE)
 write.table(gentodist.mr,file=paste(gddir,fileoutputname,sep=""),append=TRUE,sep=",",eol=",",
 row.names=TRUE,col.names=FALSE)
 write.table(gentodist.pv1,file=paste(gddir,fileoutputname,sep=""),append=TRUE,sep=",",eol=",",
 row.names=TRUE,col.names=FALSE)
 write.table(gentodist.pv2,file=paste(gddir,fileoutputname,sep=""),append=TRUE,sep=",",eol=",",
 row.names=TRUE,col.names=FALSE)
 write.table(gentodist.pv3,file=paste(gddir,fileoutputname,sep=""),append=TRUE,sep=",",eol=",",
 row.names=TRUE,col.names=FALSE)
 write.table(gentodist.llim,file=paste(gddir,fileoutputname,sep=""),append=TRUE,sep=",",eol=",",
 row.names=TRUE,col.names=FALSE)
 write.table(gentodist.ulim,file=paste(gddir,fileoutputname,sep=""),append=TRUE,sep=",",eol="\n",
 row.names=TRUE,col.names=FALSE)
 if (mgram.gentodist.ans=='Y')
 {
 # File folder header
 fileoutputname1 <- paste(landfileon,"MGramMCgentodist.csv",sep="")
 write.table(t(data.frame(gentodist.mgram$mgram[,3])),file=paste(gddir,fileoutputname1,sep=""),append=TRUE,sep=",",eol=",",
 row.names=TRUE,col.names=FALSE)
 write.table(t(data.frame(gentodist.mgram$mgram[,1])),file=paste(gddir,fileoutputname1,sep=""),append=TRUE,sep=",",eol=",",
 row.names=TRUE,col.names=FALSE)
 write.table(t(data.frame(gentodist.mgram$mgram[,4])),file=paste(gddir,fileoutputname1,sep=""),append=TRUE,sep=",",eol="\n",
 row.names=TRUE,col.names=FALSE)
 }
 }
 # Simple genetic ~ barrier
 if (gentobarr.ans=='Y')
 {
 gentobarr.mr <- t(data.frame(gentobarr.mr))
 gentobarr.pv1 <- t(data.frame(gentobarr.pv1))
 gentobarr.pv2 <- t(data.frame(gentobarr.pv2))
 gentobarr.pv3 <- t(data.frame(gentobarr.pv3))
 gentobarr.llim <- t(data.frame(gentobarr.llim))
 gentobarr.ulim <- t(data.frame(gentobarr.ulim))
 # File name
 fileoutputname <- paste(landfileon,"MRMCgentobarr.csv",sep="")
 write.table(paste(batchfiledir,mcfiledir,landfileon,sep=""),file=paste(gddir,fileoutputname,sep=""),append=TRUE,sep=",",eol=",",
 row.names=FALSE,col.names=FALSE)
 write.table(gentobarr.mr,file=paste(gddir,fileoutputname,sep=""),append=TRUE,sep=",",eol=",",
 row.names=TRUE,col.names=FALSE)
 write.table(gentobarr.pv1,file=paste(gddir,fileoutputname,sep=""),append=TRUE,sep=",",eol=",",
 row.names=TRUE,col.names=FALSE)
 write.table(gentobarr.pv2,file=paste(gddir,fileoutputname,sep=""),append=TRUE,sep=",",eol=",",
 row.names=TRUE,col.names=FALSE)
 write.table(gentobarr.pv3,file=paste(gddir,fileoutputname,sep=""),append=TRUE,sep=",",eol=",",
 row.names=TRUE,col.names=FALSE)
 write.table(gentobarr.llim,file=paste(gddir,fileoutputname,sep=""),append=TRUE,sep=",",eol=",",
 row.names=TRUE,col.names=FALSE)
 write.table(gentobarr.ulim,file=paste(gddir,fileoutputname,sep=""),append=TRUE,sep=",",eol="\n",
 row.names=TRUE,col.names=FALSE)
 if (mgram.gentobarr.ans=='Y')
 {
 # File folder header
 fileoutputname1 <- paste(landfileon,"MGramMCgentobarr.csv",sep="")
 write.table(t(data.frame(gentobarr.mgram$mgram[,3])),file=paste(gddir,fileoutputname1,sep=""),append=TRUE,sep=",",eol=",",
 row.names=TRUE,col.names=FALSE)
 write.table(t(data.frame(gentobarr.mgram$mgram[,1])),file=paste(gddir,fileoutputname1,sep=""),append=TRUE,sep=",",eol=",",
 row.names=TRUE,col.names=FALSE)
 write.table(t(data.frame(gentobarr.mgram$mgram[,4])),file=paste(gddir,fileoutputname1,sep=""),append=TRUE,sep=",",eol="\n",
 row.names=TRUE,col.names=FALSE)
 }
 }

 # Simple genetic ~ landscape
 if (gentoland.ans=='Y')
 {
 gentoland.mr <- t(data.frame(gentoland.mr))
 gentoland.pv1 <- t(data.frame(gentoland.pv1))
 gentoland.pv2 <- t(data.frame(gentoland.pv2))
 gentoland.pv3 <- t(data.frame(gentoland.pv3))
 gentoland.llim <- t(data.frame(gentoland.llim))
 gentoland.ulim <- t(data.frame(gentoland.ulim))
 # File name
 fileoutputname <- paste(landfileon,"MRMCgentoland.csv",sep="")
 write.table(paste(batchfiledir,mcfiledir,landfileon,sep=""),file=paste(gddir,fileoutputname,sep=""),append=TRUE,sep=",",eol=",",
 row.names=FALSE,col.names=FALSE)
 write.table(gentoland.mr,file=paste(gddir,fileoutputname,sep=""),append=TRUE,sep=",",eol=",",
 row.names=TRUE,col.names=FALSE)
 write.table(gentoland.pv1,file=paste(gddir,fileoutputname,sep=""),append=TRUE,sep=",",eol=",",
 row.names=TRUE,col.names=FALSE)
 write.table(gentoland.pv2,file=paste(gddir,fileoutputname,sep=""),append=TRUE,sep=",",eol=",",
 row.names=TRUE,col.names=FALSE)
 write.table(gentoland.pv3,file=paste(gddir,fileoutputname,sep=""),append=TRUE,sep=",",eol=",",
 row.names=TRUE,col.names=FALSE)
 write.table(gentoland.llim,file=paste(gddir,fileoutputname,sep=""),append=TRUE,sep=",",eol=",",
 row.names=TRUE,col.names=FALSE)
 write.table(gentoland.ulim,file=paste(gddir,fileoutputname,sep=""),append=TRUE,sep=",",eol="\n",
 row.names=TRUE,col.names=FALSE)
 if (mgram.gentoland.ans=='Y')
 {
 # File folder header
 fileoutputname1 <- paste(landfileon,"MGramMCgentoland.csv",sep="")
 write.table(t(data.frame(gentoland.mgram$mgram[,3])),file=paste(gddir,fileoutputname1,sep=""),append=TRUE,sep=",",eol=",",
 row.names=TRUE,col.names=FALSE)
 write.table(t(data.frame(gentoland.mgram$mgram[,1])),file=paste(gddir,fileoutputname1,sep=""),append=TRUE,sep=",",eol=",",
 row.names=TRUE,col.names=FALSE)
 write.table(t(data.frame(gentoland.mgram$mgram[,4])),file=paste(gddir,fileoutputname1,sep=""),append=TRUE,sep=",",eol="\n",
 row.names=TRUE,col.names=FALSE)
 }
 }

 # Partial genetic ~ distance|barrier
 if (gentodist.barr.ans=='Y')
 {
 gentodist.barr.mr <- t(data.frame(gentodist.barr.mr))
 gentodist.barr.pv1 <- t(data.frame(gentodist.barr.pv1))
 gentodist.barr.pv2 <- t(data.frame(gentodist.barr.pv2))
 gentodist.barr.pv3 <- t(data.frame(gentodist.barr.pv3))
 gentodist.barr.llim <- t(data.frame(gentodist.barr.llim))
 gentodist.barr.ulim <- t(data.frame(gentodist.barr.ulim))
 # File name
 fileoutputname <- paste(landfileon,"MRMCgentodist.barr.csv",sep="")
 write.table(paste(batchfiledir,mcfiledir,landfileon,sep=""),file=paste(gddir,fileoutputname,sep=""),append=TRUE,sep=",",eol=",",
 row.names=FALSE,col.names=FALSE)
 write.table(gentodist.barr.mr,file=paste(gddir,fileoutputname,sep=""),append=TRUE,sep=",",eol=",",
 row.names=TRUE,col.names=FALSE)
 write.table(gentodist.barr.pv1,file=paste(gddir,fileoutputname,sep=""),append=TRUE,sep=",",eol=",",
 row.names=TRUE,col.names=FALSE)
 write.table(gentodist.barr.pv2,file=paste(gddir,fileoutputname,sep=""),append=TRUE,sep=",",eol=",",
 row.names=TRUE,col.names=FALSE)
 write.table(gentodist.barr.pv3,file=paste(gddir,fileoutputname,sep=""),append=TRUE,sep=",",eol=",",
 row.names=TRUE,col.names=FALSE)
 write.table(gentodist.barr.llim,file=paste(gddir,fileoutputname,sep=""),append=TRUE,sep=",",eol=",",
 row.names=TRUE,col.names=FALSE)
 write.table(gentodist.barr.ulim,file=paste(gddir,fileoutputname,sep=""),append=TRUE,sep=",",eol="\n",
 row.names=TRUE,col.names=FALSE)
 if (mgram.gentodist.barr.ans=='Y')
 {
 # File folder header
 fileoutputname1 <- paste(landfileon,"MGramMCgentodist.barr.csv",sep="")
 write.table(t(data.frame(gentodist.barr.mgram$mgram[,3])),file=paste(gddir,fileoutputname1,sep=""),append=TRUE,sep=",",eol=",",
 row.names=TRUE,col.names=FALSE)
 write.table(t(data.frame(gentodist.barr.mgram$mgram[,1])),file=paste(gddir,fileoutputname1,sep=""),append=TRUE,sep=",",eol=",",
 row.names=TRUE,col.names=FALSE)
 write.table(t(data.frame(gentodist.barr.mgram$mgram[,4])),file=paste(gddir,fileoutputname1,sep=""),append=TRUE,sep=",",eol="\n",
 row.names=TRUE,col.names=FALSE)
 }
 }

 # Partial genetic ~ distance|landscape
 if (gentodist.land.ans=='Y')
 {
 gentodist.land.mr <- t(data.frame(gentodist.land.mr))
 gentodist.land.pv1 <- t(data.frame(gentodist.land.pv1))
 gentodist.land.pv2 <- t(data.frame(gentodist.land.pv2))
 gentodist.land.pv3 <- t(data.frame(gentodist.land.pv3))
 gentodist.land.llim <- t(data.frame(gentodist.land.llim))
 gentodist.land.ulim <- t(data.frame(gentodist.land.ulim))
 # File name
 fileoutputname <- paste(landfileon,"MRMCgentodist.land.csv",sep="")
 write.table(paste(batchfiledir,mcfiledir,landfileon,sep=""),file=paste(gddir,fileoutputname,sep=""),append=TRUE,sep=",",eol=",",
 row.names=FALSE,col.names=FALSE)
 write.table(gentodist.land.mr,file=paste(gddir,fileoutputname,sep=""),append=TRUE,sep=",",eol=",",
 row.names=TRUE,col.names=FALSE)
 write.table(gentodist.land.pv1,file=paste(gddir,fileoutputname,sep=""),append=TRUE,sep=",",eol=",",
 row.names=TRUE,col.names=FALSE)
 write.table(gentodist.land.pv2,file=paste(gddir,fileoutputname,sep=""),append=TRUE,sep=",",eol=",",
 row.names=TRUE,col.names=FALSE)
 write.table(gentodist.land.pv3,file=paste(gddir,fileoutputname,sep=""),append=TRUE,sep=",",eol=",",
 row.names=TRUE,col.names=FALSE)
 write.table(gentodist.land.llim,file=paste(gddir,fileoutputname,sep=""),append=TRUE,sep=",",eol=",",
 row.names=TRUE,col.names=FALSE)
 write.table(gentodist.land.ulim,file=paste(gddir,fileoutputname,sep=""),append=TRUE,sep=",",eol="\n",
 row.names=TRUE,col.names=FALSE)
 if (mgram.gentodist.land.ans=='Y')
 {
 # File folder header
 fileoutputname1 <- paste(landfileon,"MGramMCgentodist.land.csv",sep="")
 write.table(t(data.frame(gentodist.land.mgram$mgram[,3])),file=paste(gddir,fileoutputname1,sep=""),append=TRUE,sep=",",eol=",",
 row.names=TRUE,col.names=FALSE)
 write.table(t(data.frame(gentodist.land.mgram$mgram[,1])),file=paste(gddir,fileoutputname1,sep=""),append=TRUE,sep=",",eol=",",
 row.names=TRUE,col.names=FALSE)
 write.table(t(data.frame(gentodist.land.mgram$mgram[,4])),file=paste(gddir,fileoutputname1,sep=""),append=TRUE,sep=",",eol="\n",
 row.names=TRUE,col.names=FALSE)
 }
 }
 # Partial genetic ~ barrier|landscape
 if (gentobarr.land.ans=='Y')
 {
 gentobarr.land.mr <- t(data.frame(gentobarr.land.mr))
 gentobarr.land.pv1 <- t(data.frame(gentobarr.land.pv1))
 gentobarr.land.pv2 <- t(data.frame(gentobarr.land.pv2))
 gentobarr.land.pv3 <- t(data.frame(gentobarr.land.pv3))
 gentobarr.land.llim <- t(data.frame(gentobarr.land.llim))
 gentobarr.land.ulim <- t(data.frame(gentobarr.land.ulim))
 # File name
 fileoutputname <- paste(landfileon,"MRMCgentobarr.land.csv",sep="")
 write.table(paste(batchfiledir,mcfiledir,landfileon,sep=""),file=paste(gddir,fileoutputname,sep=""),append=TRUE,sep=",",eol=",",
 row.names=FALSE,col.names=FALSE)
 write.table(gentobarr.land.mr,file=paste(gddir,fileoutputname,sep=""),append=TRUE,sep=",",eol=",",
 row.names=TRUE,col.names=FALSE)
 write.table(gentobarr.land.pv1,file=paste(gddir,fileoutputname,sep=""),append=TRUE,sep=",",eol=",",
 row.names=TRUE,col.names=FALSE)
 write.table(gentobarr.land.pv2,file=paste(gddir,fileoutputname,sep=""),append=TRUE,sep=",",eol=",",
 row.names=TRUE,col.names=FALSE)
 write.table(gentobarr.land.pv3,file=paste(gddir,fileoutputname,sep=""),append=TRUE,sep=",",eol=",",
 row.names=TRUE,col.names=FALSE)
 write.table(gentobarr.land.llim,file=paste(gddir,fileoutputname,sep=""),append=TRUE,sep=",",eol=",",
 row.names=TRUE,col.names=FALSE)
 write.table(gentobarr.land.ulim,file=paste(gddir,fileoutputname,sep=""),append=TRUE,sep=",",eol="\n",
 row.names=TRUE,col.names=FALSE)
 if (mgram.gentobarr.land.ans=='Y')
 {
 # File folder header
 fileoutputname1 <- paste(landfileon,"MGramMCgentobarr.land.csv",sep="")
 write.table(t(data.frame(gentobarr.land.mgram$mgram[,3])),file=paste(gddir,fileoutputname1,sep=""),append=TRUE,sep=",",eol=",",
 row.names=TRUE,col.names=FALSE)
 write.table(t(data.frame(gentobarr.land.mgram$mgram[,1])),file=paste(gddir,fileoutputname1,sep=""),append=TRUE,sep=",",eol=",",
 row.names=TRUE,col.names=FALSE)
 write.table(t(data.frame(gentobarr.land.mgram$mgram[,4])),file=paste(gddir,fileoutputname1,sep=""),append=TRUE,sep=",",eol="\n",
 row.names=TRUE,col.names=FALSE)
 }
 }

 # Partial genetic ~ barrier|distance
 if (gentobarr.dist.ans=='Y')
 {
 gentobarr.dist.mr <- t(data.frame(gentobarr.dist.mr))
 gentobarr.dist.pv1 <- t(data.frame(gentobarr.dist.pv1))
 gentobarr.dist.pv2 <- t(data.frame(gentobarr.dist.pv2))
 gentobarr.dist.pv3 <- t(data.frame(gentobarr.dist.pv3))
 gentobarr.dist.llim <- t(data.frame(gentobarr.dist.llim))
 gentobarr.dist.ulim <- t(data.frame(gentobarr.dist.ulim))
 # File name
 fileoutputname <- paste(landfileon,"MRMCgentobarr.dist.csv",sep="")
 write.table(paste(batchfiledir,mcfiledir,landfileon,sep=""),file=paste(gddir,fileoutputname,sep=""),append=TRUE,sep=",",eol=",",
 row.names=FALSE,col.names=FALSE)
 write.table(gentobarr.dist.mr,file=paste(gddir,fileoutputname,sep=""),append=TRUE,sep=",",eol=",",
 row.names=TRUE,col.names=FALSE)
 write.table(gentobarr.dist.pv1,file=paste(gddir,fileoutputname,sep=""),append=TRUE,sep=",",eol=",",
 row.names=TRUE,col.names=FALSE)
 write.table(gentobarr.dist.pv2,file=paste(gddir,fileoutputname,sep=""),append=TRUE,sep=",",eol=",",
 row.names=TRUE,col.names=FALSE)
 write.table(gentobarr.dist.pv3,file=paste(gddir,fileoutputname,sep=""),append=TRUE,sep=",",eol=",",
 row.names=TRUE,col.names=FALSE)
 write.table(gentobarr.dist.llim,file=paste(gddir,fileoutputname,sep=""),append=TRUE,sep=",",eol=",",
 row.names=TRUE,col.names=FALSE)
 write.table(gentobarr.dist.ulim,file=paste(gddir,fileoutputname,sep=""),append=TRUE,sep=",",eol="\n",
 row.names=TRUE,col.names=FALSE)
 if (mgram.gentobarr.dist.ans=='Y')
 {
 # File folder header
 fileoutputname1 <- paste(landfileon,"MGramMCgentobarr.dist.csv",sep="")
 write.table(t(data.frame(gentobarr.dist.mgram$mgram[,3])),file=paste(gddir,fileoutputname1,sep=""),append=TRUE,sep=",",eol=",",
 row.names=TRUE,col.names=FALSE)
 write.table(t(data.frame(gentobarr.dist.mgram$mgram[,1])),file=paste(gddir,fileoutputname1,sep=""),append=TRUE,sep=",",eol=",",
 row.names=TRUE,col.names=FALSE)
 write.table(t(data.frame(gentobarr.dist.mgram$mgram[,4])),file=paste(gddir,fileoutputname1,sep=""),append=TRUE,sep=",",eol="\n",
 row.names=TRUE,col.names=FALSE)
 }
 }

 # Partial genetic ~ landscape|distance
 if (gentoland.dist.ans=='Y')
 {
 gentoland.dist.mr <- t(data.frame(gentoland.dist.mr))
 gentoland.dist.pv1 <- t(data.frame(gentoland.dist.pv1))
 gentoland.dist.pv2 <- t(data.frame(gentoland.dist.pv2))
 gentoland.dist.pv3 <- t(data.frame(gentoland.dist.pv3))
 gentoland.dist.llim <- t(data.frame(gentoland.dist.llim))
 gentoland.dist.ulim <- t(data.frame(gentoland.dist.ulim))
 # File name
 fileoutputname <- paste(landfileon,"MRMCgentoland.dist.csv",sep="")
 write.table(paste(batchfiledir,mcfiledir,landfileon,sep=""),file=paste(gddir,fileoutputname,sep=""),append=TRUE,sep=",",eol=",",
 row.names=FALSE,col.names=FALSE)
 write.table(gentoland.dist.mr,file=paste(gddir,fileoutputname,sep=""),append=TRUE,sep=",",eol=",",
 row.names=TRUE,col.names=FALSE)
 write.table(gentoland.dist.pv1,file=paste(gddir,fileoutputname,sep=""),append=TRUE,sep=",",eol=",",
 row.names=TRUE,col.names=FALSE)
 write.table(gentoland.dist.pv2,file=paste(gddir,fileoutputname,sep=""),append=TRUE,sep=",",eol=",",
 row.names=TRUE,col.names=FALSE)
 write.table(gentoland.dist.pv3,file=paste(gddir,fileoutputname,sep=""),append=TRUE,sep=",",eol=",",
 row.names=TRUE,col.names=FALSE)
 write.table(gentoland.dist.llim,file=paste(gddir,fileoutputname,sep=""),append=TRUE,sep=",",eol=",",
 row.names=TRUE,col.names=FALSE)
 write.table(gentoland.dist.ulim,file=paste(gddir,fileoutputname,sep=""),append=TRUE,sep=",",eol="\n",
 row.names=TRUE,col.names=FALSE)
 if (mgram.gentoland.dist.ans=='Y')
 {
 # File folder header
 fileoutputname1 <- paste(landfileon,"MGramMCgentoland.dist.csv",sep="")
 write.table(t(data.frame(gentoland.dist.mgram$mgram[,3])),file=paste(gddir,fileoutputname1,sep=""),append=TRUE,sep=",",eol=",",
 row.names=TRUE,col.names=FALSE)
 write.table(t(data.frame(gentoland.dist.mgram$mgram[,1])),file=paste(gddir,fileoutputname1,sep=""),append=TRUE,sep=",",eol=",",
 row.names=TRUE,col.names=FALSE)
 write.table(t(data.frame(gentoland.dist.mgram$mgram[,4])),file=paste(gddir,fileoutputname1,sep=""),append=TRUE,sep=",",eol="\n",
 row.names=TRUE,col.names=FALSE)
 }
 }

 # Partial genetic ~ landscape|barrier
 if (gentoland.barr.ans=='Y')
 {
 gentoland.barr.mr <- t(data.frame(gentoland.barr.mr))
 gentoland.barr.pv1 <- t(data.frame(gentoland.barr.pv1))
 gentoland.barr.pv2 <- t(data.frame(gentoland.barr.pv2))
 gentoland.barr.pv3 <- t(data.frame(gentoland.barr.pv3))
 gentoland.barr.llim <- t(data.frame(gentoland.barr.llim))
 gentoland.barr.ulim <- t(data.frame(gentoland.barr.ulim))
 # File name
 fileoutputname <- paste(landfileon,"MRMCgentoland.barr.csv",sep="")
 write.table(paste(batchfiledir,mcfiledir,landfileon,sep=""),file=paste(gddir,fileoutputname,sep=""),append=TRUE,sep=",",eol=",",
 row.names=FALSE,col.names=FALSE)
 write.table(gentoland.barr.mr,file=paste(gddir,fileoutputname,sep=""),append=TRUE,sep=",",eol=",",
 row.names=TRUE,col.names=FALSE)
 write.table(gentoland.barr.pv1,file=paste(gddir,fileoutputname,sep=""),append=TRUE,sep=",",eol=",",
 row.names=TRUE,col.names=FALSE)
 write.table(gentoland.barr.pv2,file=paste(gddir,fileoutputname,sep=""),append=TRUE,sep=",",eol=",",
 row.names=TRUE,col.names=FALSE)
 write.table(gentoland.barr.pv3,file=paste(gddir,fileoutputname,sep=""),append=TRUE,sep=",",eol=",",
 row.names=TRUE,col.names=FALSE)
 write.table(gentoland.barr.llim,file=paste(gddir,fileoutputname,sep=""),append=TRUE,sep=",",eol=",",
 row.names=TRUE,col.names=FALSE)
 write.table(gentoland.barr.ulim,file=paste(gddir,fileoutputname,sep=""),append=TRUE,sep=",",eol="\n",
 row.names=TRUE,col.names=FALSE)
 if (mgram.gentoland.barr.ans=='Y')
 {
 # File folder header
 fileoutputname1 <- paste(landfileon,"MGramMCgentoland.barr.csv",sep="")
 write.table(t(data.frame(gentoland.barr.mgram$mgram[,3])),file=paste(gddir,fileoutputname1,sep=""),append=TRUE,sep=",",eol=",",
 row.names=TRUE,col.names=FALSE)
 write.table(t(data.frame(gentoland.barr.mgram$mgram[,1])),file=paste(gddir,fileoutputname1,sep=""),append=TRUE,sep=",",eol=",",
 row.names=TRUE,col.names=FALSE)
 write.table(t(data.frame(gentoland.barr.mgram$mgram[,4])),file=paste(gddir,fileoutputname1,sep=""),append=TRUE,sep=",",eol="\n",
 row.names=TRUE,col.names=FALSE)
 }
 }

 # Partial genetic ~ landscape|landscape
 if (gentoland.land.ans=='Y')
 {
 gentoland.land.mr <- t(data.frame(gentoland.land.mr))
 gentoland.land.pv1 <- t(data.frame(gentoland.land.pv1))
 gentoland.land.pv2 <- t(data.frame(gentoland.land.pv2))
 gentoland.land.pv3 <- t(data.frame(gentoland.land.pv3))
 gentoland.land.llim <- t(data.frame(gentoland.land.llim))
 gentoland.land.ulim <- t(data.frame(gentoland.land.ulim))
 # File name
 fileoutputname <- paste(landfileon,"MRMCgentoland.land.csv",sep="")
 write.table(paste(batchfiledir,mcfiledir,landfileon,sep=""),file=paste(gddir,fileoutputname,sep=""),append=TRUE,sep=",",eol=",",
 row.names=FALSE,col.names=FALSE)
 write.table(gentoland.land.mr,file=paste(gddir,fileoutputname,sep=""),append=TRUE,sep=",",eol=",",
 row.names=TRUE,col.names=FALSE)
 write.table(gentoland.land.pv1,file=paste(gddir,fileoutputname,sep=""),append=TRUE,sep=",",eol=",",
 row.names=TRUE,col.names=FALSE)
 write.table(gentoland.land.pv2,file=paste(gddir,fileoutputname,sep=""),append=TRUE,sep=",",eol=",",
 row.names=TRUE,col.names=FALSE)
 write.table(gentoland.land.pv3,file=paste(gddir,fileoutputname,sep=""),append=TRUE,sep=",",eol=",",
 row.names=TRUE,col.names=FALSE)
 write.table(gentoland.land.llim,file=paste(gddir,fileoutputname,sep=""),append=TRUE,sep=",",eol=",",
 row.names=TRUE,col.names=FALSE)
 write.table(gentoland.land.ulim,file=paste(gddir,fileoutputname,sep=""),append=TRUE,sep=",",eol="\n",
 row.names=TRUE,col.names=FALSE)
 if (mgram.gentoland.land.ans=='Y')
 {
 # File folder header
 fileoutputname1 <- paste(landfileon,"MGramMCgentoland.land.csv",sep="")
 write.table(t(data.frame(gentoland.land.mgram$mgram[,3])),file=paste(gddir,fileoutputname1,sep=""),append=TRUE,sep=",",eol=",",
 row.names=TRUE,col.names=FALSE)
 write.table(t(data.frame(gentoland.land.mgram$mgram[,1])),file=paste(gddir,fileoutputname1,sep=""),append=TRUE,sep=",",eol=",",
 row.names=TRUE,col.names=FALSE)
 write.table(t(data.frame(gentoland.land.mgram$mgram[,4])),file=paste(gddir,fileoutputname1,sep=""),append=TRUE,sep=",",eol="\n",
 row.names=TRUE,col.names=FALSE)
 }
 } # End of last write out file

 }# Monte Carlo Loop End
 }# End second landscape loop
 }# Pt/First landscape loop End

 }# Batch Loop End

}# Function End

The partial.cor function calculates the partial correlation between two distance matrices 'xmat' and 'ymat' after partialling out the effects of a third matrix 'zmat' via linear regression. For right now this function only returns the residuals.

partial.cor <- function(xmat,ymat,zmat)
{
 xval <- xmat[row(xmat)>col(xmat)]
 yval <- ymat[row(ymat)>col(ymat)]
 zval <- zmat[row(zmat)>col(zmat)]
 xresid <- lm(xval~zval)$resid
 yresid <- lm(yval~zval)$resid
 parcor.resid <- lm(xresid~yresid)$resid
 list(parcor.resid=parcor.resid)
}

# R code running mantel.mc Mantel test function

05.25.2012-08:34 (ell) version Multiple Landscapes -- Modified for general studies for testing multiple landscapes. Added option for log transformation testing. 09.24.2010-10:04 (ell) version Animal Movement -- Sent to Animal movement group to test 135 landscapes X 6 point cominations. Options include genetic ~ landscape1|landscape{N} as well as genetic ~ landscape1|distance1. version SAM2 -- Sent to S.Cushman Feb 23, 2010 to test 36 landscapes. This version runs through a list of landscapes, running partial mantel on genetic ~ landscape1|landscape2. Erin L. Landguth MRMCRun.R
 Project Description: an example script to run the function mantel.mc Libraries Needed: 1. Spatial 2. Ecodist Project Input: 1. Function parameter inputs... 2. MRMCFun.R (mantel.mc function) must be loaded into workspace before this script is ran.
 Creation Date: December 10, 2008

Load Library

library(spatial)
library(ecodist)

1. Function parameter inputs

# This is the number of CDPOP simulations you ran at your Institution
# Only do one at a time, will have to rename folders everytime you
# run a this code needs to be '...'batchrun0'...', one quick way to do this
# is to add unique batchrun number (e.g., 0, 1, 2, 3...) to the batchstring
# parameter in next line (e.g., batchstring0, batchstring1, ...):


batchno <- 1

# This is the 'dos' style unique time stamp header in front of
# batchrun{}mcrun{}, copy and paste this here with quotes around it:

batchstring <- '1409940034'

# Specify the number of Monte Carlo runs used for each CDPOP simulation
mcrunno <- 50
mcstart <- 1 # In case your runs stop and you can restart at given monte carlo run
iptstart <- 1 # In case your runs stop and you can restart at given landscape loop

# The total individuals in each file
N <- 1000

# Uncomment one of these styles for nthfile analysis, the files written out
nthfile <- c(25,500)

# Genetic distance file location
gddir <- "C:/cdpop/10U_truth/rep5/5R5N05_10Ulastone/"

# Genetci distance file name (do not change)
gdfilename <- 'Gdmatrix'

# Barrier distance file location
barrdir <- "/blank/"

# Barrier distance file name
barrfilename <- c("ModelMatrix1000.csv")

# Do you want to test for the effect of the barrfilename? 'Y' or 'N'
barrans <- 'N'

# Euclidean distance file location
distdir <- paste("C:/cdpop/10U_truth/rep5/5R5N05_10Ulastone/"
,batchstring,"batchrun0mcrun0/",sep="")

# Euclidean distance file name(s) - Enter ED files here.
distfilename <- c("Edmatrix1000.csv")

# Do you want to test for the effects of distfilename? 'Y' or 'N'
distans <- 'Y'

# Landscape distance file location
landdir <- "C:/cdpop/10U_truth/rep5/5R5N05_10Ulastone/"

# Landscape distance file name(s)
landfilename <- c("5R5N05Cont10U.txt_xydata_5R5N05Cont10U.csv",
 "5R5N05Cont10SQ.txt_xydata_5R5N05Cont10U.csv",
 "5R5N05Cont100U.txt_xydata_5R5N05Cont10U.csv",
 "5R5N05Cont100SQ.txt_xydata_5R5N05Cont10U.csv",
 "T5R5N05Cont10U.txt_xydata_5R5N05Cont10U.csv")

# Do you want to test for the effect of the landfilename? 'Y' or 'N'
landans <- 'Y'

# Do you want to test gd ~ log(landscape)
logans <- 'Y'

# Now specify the sample style to use:
# known = a known set of indeces to be read from a file
# random = a random n draw from the total
# all = run analysis on all points
samplestyle <- 'all'
# If samplestyle = 'random'
sampleno <- 200
# Else if samplestyle = 'known'
sampledir <- "C:/BLANK.csv"

# Here specify Mantel Test run information 9 of them:
# Y or N to the specific simple and partial Mantel Tests
# Define number of permutations for significance test
gentodist.ans <- 'N' # Simple genetic ~ distance
gentobarr.ans <- 'N' # Simple genetic ~ barrier
gentoland.ans <- 'N' # Simple genetic ~ landscape
gentodist.barr.ans <- 'N' # Partial genetic ~ distance|barrier
gentodist.land.ans <- 'N' # Partial genetic ~ distance|landscape
gentobarr.dist.ans <- 'N' # Partial genetic ~ barrier|distance
gentobarr.land.ans <- 'N' # Partial genetic ~ barrier|landscape
gentoland.dist.ans <- 'N' # Partial genetic ~ landscape|distance
gentoland.barr.ans <- 'N' # Partial genetic ~ landscape|barrier
gentoland.land.ans <- 'Y' # Partial genetic ~ landscape|landscape
mperms <- 1999 # Mantel permutations
mrank <- FALSE # False for Pearson correlation, TRUE for Spearman correlation
nboot <- 0 # Number of bootstrap samples, 0 to turn off
pboot <- 0.9 # Level to resample bootstrap
cboot <- 0.95 # CI estimates

# Here specifiy Mantel Correlogram to run
# Y or N to the specific Mantel correlogram
# Define year to run test at
mgram.gentodist.ans <- 'N' # Simple genetic ~ distance
mgram.gentobarr.ans <- 'N' # Simple genetic ~ barrier
mgram.gentoland.ans <- 'N' # Simple genetic ~ landscape
mgram.gentodist.barr.ans <- 'N' # Partial genetic ~ distance|barrier
mgram.gentodist.land.ans <- 'N' # Partial genetic ~ distance|landscape
mgram.gentobarr.dist.ans <- 'N' # Partial genetic ~ barrier|distance
mgram.gentobarr.land.ans <- 'N' # Partial genetic ~ barrier|landscape
mgram.gentoland.dist.ans <- 'N' # Partial genetic ~ landscape|distance
mgram.gentoland.barr.ans <- 'N' # Partial genetic ~ landscape|barrier
mgram.gentoland.land.ans <- 'N' # Partial genetic ~ landscape|landscape
mgramruntime <- 100

Run the Mantel Function code

mantel.mc(batchno,mcrunno,N,nthfile,gddir,gdfilename,barrdir,barrfilename,barrans,
 distdir,distfilename,distans,landdir,landfilename,landans,samplestyle,sampleno,sampledir,gentodist.ans,
 gentobarr.ans,gentoland.ans,gentodist.barr.ans,gentodist.land.ans,gentobarr.dist.ans,gentobarr.land.ans,
 gentoland.dist.ans,gentoland.barr.ans,gentoland.land.ans,mperms,mgram.gentodist.ans,mgram.gentobarr.ans,mgram.gentoland.ans,
 mgram.gentodist.barr.ans,mgram.gentodist.land.ans,mgram.gentobarr.dist.ans,mgram.gentobarr.land.ans,
 mgram.gentoland.dist.ans,mgram.gentoland.barr.ans,mgram.gentoland.land.ans,mgramruntime,batchstring,logans,mrank,
 nboot, pboot, cboot,mc

# R code for Mantel-based Tests and Method Performance

User inputs

alpha <- 0.005 # set significance level for Mantel tests

MCs <- 50 # set number of MC runs in CDPOP

equilibrium <- FALSE # specify generation (TRUE = equilibrium, FALSE = generation 25)

if(equilibrium==TRUE) { # specify column number in Mantel csv files that contains p-values and Mantel r values at the desired generation
 pvals.column <- 7
 mantelr.column <- 4
} else {
 pvals.column <- 6
 mantelr.column <- 3
}

truth.element <- 2 # specify which element from landscapes vectors (below) is the true landscape (= 2 if 10U is truth)

test.element <- 5 # specifiy which element from landscapes vectors is the test (i.e., discriminant) landscape

nclusters <- 4 # number of landscape clusters

nreps <- 5 # number of replicates per cluster

logtrans <- TRUE # specify whether cost distances are log transformed (TRUE = logged, FALSE = not logged)

folder <- "C:/Users/Creecht/Desktop/DGS Simulation Study/Mantel runs/Truth10U/" # specify outer folder where Mantel files are stored

Combine landscape names for each cluster and each replicate

clusters <- c("R5N0", "R5N05", "R100N0", "R100N05")
C1.rep1.landscapes <- c("1R5N0Cont10SQ", "1R5N0Cont10U", "1R5N0Cont100SQ", "1R5N0Cont100U","T1R5N0Cont10U")
C1.rep2.landscapes <- c("2R5N0Cont10SQ", "2R5N0Cont10U", "2R5N0Cont100SQ", "2R5N0Cont100U","T2R5N0Cont10U")
C1.rep3.landscapes <- c("3R5N0Cont10SQ", "3R5N0Cont10U", "3R5N0Cont100SQ", "3R5N0Cont100U","T3R5N0Cont10U")
C1.rep4.landscapes <- c("4R5N0Cont10SQ", "4R5N0Cont10U", "4R5N0Cont100SQ", "4R5N0Cont100U","T4R5N0Cont10U")
C1.rep5.landscapes <- c("5R5N0Cont10SQ", "5R5N0Cont10U", "5R5N0Cont100SQ", "5R5N0Cont100U","T5R5N0Cont10U")
C2.rep1.landscapes <- c("1R5N05Cont10SQ", "1R5N05Cont10U", "1R5N05Cont100SQ", "1R5N05Cont100U","T1R5N05Cont10U")
C2.rep2.landscapes <- c("2R5N05Cont10SQ", "2R5N05Cont10U", "2R5N05Cont100SQ", "2R5N05Cont100U","T2R5N05Cont10U")
C2.rep3.landscapes <- c("3R5N05Cont10SQ", "3R5N05Cont10U", "3R5N05Cont100SQ", "3R5N05Cont100U","T3R5N05Cont10U")
C2.rep4.landscapes <- c("4R5N05Cont10SQ", "4R5N05Cont10U", "4R5N05Cont100SQ", "4R5N05Cont100U","T4R5N05Cont10U")
C2.rep5.landscapes <- c("5R5N05Cont10SQ", "5R5N05Cont10U", "5R5N05Cont100SQ", "5R5N05Cont100U","T5R5N05Cont10U")
C3.rep1.landscapes <- c("1R100N0Cont10SQ", "1R100N0Cont10U", "1R100N0Cont100SQ", "1R100N0Cont100U","T1R100N0Cont10U")
C3.rep2.landscapes <- c("2R100N0Cont10SQ", "2R100N0Cont10U", "2R100N0Cont100SQ", "2R100N0Cont100U","T2R100N0Cont10U")
C3.rep3.landscapes <- c("3R100N0Cont10SQ", "3R100N0Cont10U", "3R100N0Cont100SQ", "3R100N0Cont100U","T3R100N0Cont10U")
C3.rep4.landscapes <- c("4R100N0Cont10SQ", "4R100N0Cont10U", "4R100N0Cont100SQ", "4R100N0Cont100U","T4R100N0Cont10U")
C3.rep5.landscapes <- c("5R100N0Cont10SQ", "5R100N0Cont10U", "5R100N0Cont100SQ", "5R100N0Cont100U","T5R100N0Cont10U")
C4.rep1.landscapes <- c("1R100N05Cont10SQ", "1R100N05Cont10U", "1R100N05Cont100SQ", "1R100N05Cont100U","T1R100N05Cont10U")
C4.rep2.landscapes <- c("2R100N05Cont10SQ", "2R100N05Cont10U", "2R100N05Cont100SQ", "2R100N05Cont100U","T2R100N05Cont10U")
C4.rep3.landscapes <- c("3R100N05Cont10SQ", "3R100N05Cont10U", "3R100N05Cont100SQ", "3R100N05Cont100U","T3R100N05Cont10U")
C4.rep4.landscapes <- c("4R100N05Cont10SQ", "4R100N05Cont10U", "4R100N05Cont100SQ", "4R100N05Cont100U","T4R100N05Cont10U")
C4.rep5.landscapes <- c("5R100N05Cont10SQ", "5R100N05Cont10U", "5R100N05Cont100SQ", "5R100N05Cont100U","T5R100N05Cont10U")

Combine file pathways for each replicate within a cluster

if(logtrans==TRUE) { # for logY files
 C1.dirs <- c(paste(folder,"Rep1/rep1cluster1logY/",sep=""), paste(folder,"Rep2/rep2cluster1logY/",sep=""), paste(folder,"Rep3/rep3cluster1logY/",sep=""), paste(folder,"Rep4/rep4cluster1logY/",sep=""), paste(folder,"Rep5/rep5cluster1logY/",sep=""))
 C2.dirs <- c(paste(folder,"Rep1/rep1cluster2logY/",sep=""), paste(folder,"Rep2/rep2cluster2logY/",sep=""), paste(folder,"Rep3/rep3cluster2logY/",sep=""), paste(folder,"Rep4/rep4cluster2logY/",sep=""), paste(folder,"Rep5/rep5cluster2logY/",sep=""))
 C3.dirs <- c(paste(folder,"Rep1/rep1cluster3logY/",sep=""), paste(folder,"Rep2/rep2cluster3logY/",sep=""), paste(folder,"Rep3/rep3cluster3logY/",sep=""), paste(folder,"Rep4/rep4cluster3logY/",sep=""), paste(folder,"Rep5/rep5cluster3logY/",sep=""))
 C4.dirs <- c(paste(folder,"Rep1/rep1cluster4logY/",sep=""), paste(folder,"Rep2/rep2cluster4logY/",sep=""), paste(folder,"Rep3/rep3cluster4logY/",sep=""), paste(folder,"Rep4/rep4cluster4logY/",sep=""), paste(folder,"Rep5/rep5cluster4logY/",sep=""))
} else { # for logN files
 C1.dirs <- c(paste(folder,"Rep1/rep1cluster1logN/",sep=""), paste(folder,"Rep2/rep2cluster1logN/",sep=""), paste(folder,"Rep3/rep3cluster1logN/",sep=""), paste(folder,"Rep4/rep4cluster1logN/",sep=""), paste(folder,"Rep5/rep5cluster1logN/",sep=""))
 C2.dirs <- c(paste(folder,"Rep1/rep1cluster2logN/",sep=""), paste(folder,"Rep2/rep2cluster2logN/",sep=""), paste(folder,"Rep3/rep3cluster2logN/",sep=""), paste(folder,"Rep4/rep4cluster2logN/",sep=""), paste(folder,"Rep5/rep5cluster2logN/",sep=""))
 C3.dirs <- c(paste(folder,"Rep1/rep1cluster3logN/",sep=""), paste(folder,"Rep2/rep2cluster3logN/",sep=""), paste(folder,"Rep3/rep3cluster3logN/",sep=""), paste(folder,"Rep4/rep4cluster3logN/",sep=""), paste(folder,"Rep5/rep5cluster3logN/",sep=""))
 C4.dirs <- c(paste(folder,"Rep1/rep1cluster4logN/",sep=""), paste(folder,"Rep2/rep2cluster4logN/",sep=""), paste(folder,"Rep3/rep3cluster4logN/",sep=""), paste(folder,"Rep4/rep4cluster4logN/",sep=""), paste(folder,"Rep5/rep5cluster4logN/",sep=""))
}

Combine Moran's I values for surfaces for each rep and each cluster NOTE: order of values is different than in Table 1 - this is because landscapes in this code are listed in different order than in Table 1

C1.rep1.MoranI <- c(0.887,0.892,0.887,0.892,0.903)
C1.rep2.MoranI <- c(0.882,0.891,0.882,0.891,0.903)
C1.rep3.MoranI <- c(0.884,0.891,0.884,0.891,0.903)
C1.rep4.MoranI <- c(0.883,0.891,0.883,0.891,0.900)
C1.rep5.MoranI <- c(0.881,0.888,0.881,0.888,0.904)
C2.rep1.MoranI <- c(0.420,0.434,0.420,0.434,0.494)
C2.rep2.MoranI <- c(0.420,0.435,0.420,0.435,0.496)
C2.rep3.MoranI <- c(0.420,0.432,0.420,0.432,0.498)
C2.rep4.MoranI <- c(0.327,0.341,0.327,0.341,0.492)
C2.rep5.MoranI <- c(0.321,0.336,0.321,0.336,0.497)
C3.rep1.MoranI <- c(0.990,0.991,0.990,0.991,0.991)
C3.rep2.MoranI <- c(0.987,0.990,0.987,0.990,0.992)
C3.rep3.MoranI <- c(0.990,0.991,0.990,0.991,0.993)
C3.rep4.MoranI <- c(0.992,0.992,0.992,0.992,0.991)
C3.rep5.MoranI <- c(0.989,0.990,0.989,0.990,0.992)
C4.rep1.MoranI <- c(0.472,0.485,0.472,0.485,0.532)
C4.rep2.MoranI <- c(0.450,0.457,0.450,0.457,0.528)
C4.rep3.MoranI <- c(0.456,0.468,0.456,0.468,0.543)
C4.rep4.MoranI <- c(0.360,0.381,0.360,0.381,0.524)
C4.rep5.MoranI <- c(0.342,0.370,0.342,0.370,0.528)

Combine Pearson correlations of cost-distances between the true and alternative surfaces for each rep of each cluster

C1.rep1.Pearson <- c(0.997,1.000,0.957,0.992,0.940)
C1.rep2.Pearson <- c(0.996,1.000,0.944,0.992,0.953)
C1.rep3.Pearson <- c(0.998,1.000,0.960,0.994,0.959)
C1.rep4.Pearson <- c(0.998,1.000,0.963,0.993,0.966)
C1.rep5.Pearson <- c(0.998,1.000,0.963,0.993,0.966)
C2.rep1.Pearson <- c(0.999,1.000,0.969,0.993,0.966)
C2.rep2.Pearson <- c(0.999,1.000,0.949,0.991,0.968)
C2.rep3.Pearson <- c(0.999,1.000,0.972,0.995,0.977)
C2.rep4.Pearson <- c(0.998,1.000,0.972,0.995,0.973)
C2.rep5.Pearson <- c(0.972,1.000,0.972,0.995,0.973)
C3.rep1.Pearson <- c(0.990,1.000,0.901,0.987,0.797)
C3.rep2.Pearson <- c(0.988,1.000,0.877,0.986,0.823)
C3.rep3.Pearson <- c(0.991,1.000,0.928,0.989,0.895)
C3.rep4.Pearson <- c(0.989,1.000,0.915,0.993,0.912)
C3.rep5.Pearson <- c(0.989,1.000,0.915,0.993,0.912)
C4.rep1.Pearson <- c(0.998,1.000,0.905,0.979,0.873)
C4.rep2.Pearson <- c(0.997,1.000,0.874,0.977,0.874)
C4.rep3.Pearson <- c(0.998,1.000,0.923,0.982,0.924)
C4.rep4.Pearson <- c(0.997,1.000,0.904,0.975,0.797)
C4.rep5.Pearson <- c(0.997,1.000,0.904,0.975,0.797)

Create data frame to store surface performance results (one row per cluster/surface/replicate combo)

my.df <- data.frame(cluster=rep(NA,(length(C1.rep1.landscapes)*nclusters*nreps)), landscape=rep(NA,(length(C1.rep1.landscapes)*nclusters*nreps)), replicate=rep(NA,(length(C1.rep1.landscapes)*nclusters*nreps)), folderpath=rep(NA,(length(C1.rep1.landscapes)*nclusters*nreps)), MoranI=rep(NA,(length(C1.rep1.landscapes)*nclusters*nreps)), Pearson=rep(NA,(length(C1.rep1.landscapes)*nclusters*nreps)),mean.consistent=rep(NA,(length(C1.rep1.landscapes)*nclusters*nreps)), prop.perfect.p=rep(NA,(length(C1.rep1.landscapes)*nclusters*nreps)), mean.RS=rep(NA,(length(C1.rep1.landscapes)*nclusters*nreps)), prop.perfect.r=rep(NA,(length(C1.rep1.landscapes)*nclusters*nreps)))

my.df$cluster <- rep(clusters, each=(length(C1.rep1.landscapes)*nreps))

my.df$landscape <- c(C1.rep1.landscapes,C1.rep2.landscapes,C1.rep3.landscapes,C1.rep4.landscapes,C1.rep5.landscapes,C2.rep1.landscapes,C2.rep2.landscapes,C2.rep3.landscapes,C2.rep4.landscapes,C2.rep5.landscapes,C3.rep1.landscapes,C3.rep2.landscapes,C3.rep3.landscapes,C3.rep4.landscapes,C3.rep5.landscapes,C4.rep1.landscapes,C4.rep2.landscapes,C4.rep3.landscapes,C4.rep4.landscapes,C4.rep5.landscapes)

my.df$replicate <- rep(rep(c(1:nreps), each=length(C1.rep1.landscapes)),nclusters)

my.df$folderpath <- c(rep(C1.dirs, each=length(C1.rep1.landscapes)),rep(C2.dirs, each=length(C2.rep1.landscapes)),rep(C3.dirs, each=length(C3.rep1.landscapes)),rep(C4.dirs, each=length(C4.rep1.landscapes)))

my.df$MoranI <- c(C1.rep1.MoranI,C1.rep2.MoranI,C1.rep3.MoranI,C1.rep4.MoranI,C1.rep5.MoranI,C2.rep1.MoranI,C2.rep2.MoranI,C2.rep3.MoranI,C2.rep4.MoranI,C2.rep5.MoranI,C3.rep1.MoranI,C3.rep2.MoranI,C3.rep3.MoranI,C3.rep4.MoranI,C3.rep5.MoranI,C4.rep1.MoranI,C4.rep2.MoranI,C4.rep3.MoranI,C4.rep4.MoranI,C4.rep5.MoranI)

my.df$Pearson <- c(C1.rep1.Pearson,C1.rep2.Pearson,C1.rep3.Pearson,C1.rep4.Pearson,C1.rep5.Pearson,C2.rep1.Pearson,C2.rep2.Pearson,C2.rep3.Pearson,C2.rep4.Pearson,C2.rep5.Pearson,C3.rep1.Pearson,C3.rep2.Pearson,C3.rep3.Pearson,C3.rep4.Pearson,C3.rep5.Pearson,C4.rep1.Pearson,C4.rep2.Pearson,C4.rep3.Pearson,C4.rep4.Pearson,C4.rep5.Pearson)

Create data frame to store proportion of unequivocal successes in 50 MC runs (one row per cluster/replicate combo)

success.df <- data.frame(cluster=rep(NA, (nclusters*nreps)), replicate=rep(NA, (nclusters*nreps)), unequivocal.p=rep(NA, (nclusters*nreps)), unequivocal.p.test=rep(NA, (nclusters*nreps)), unequivocal.p.test.just2=rep(NA, (nclusters*nreps)), unequivocal.p.leastsim.just2=rep(NA, (nclusters*nreps)), unequivocal.RS=rep(NA, (nclusters*nreps)), unequivocal.RS.test=rep(NA, (nclusters*nreps)), unequivocal.RS.test.just2=rep(NA, (nclusters*nreps)), unequivocal.RS.leastsim.just2=rep(NA, (nclusters*nreps)), unequivocal.r.simple=rep(NA, (nclusters*nreps)), unequivocal.r.simple.test=rep(NA, (nclusters*nreps)), unequivocal.r.simple.leastsim=rep(NA, (nclusters*nreps)))

success.df$cluster <- rep(clusters, each=nreps)

success.df$replicate <- rep(c(1:nreps),nclusters)

Conduct Causal Modeling, Relative Support, and simple Mantel r methods

for(y in 1:nclusters) { # loop through clusters
 for(z in 1:nreps) { # loop through replicates
 # set working directory to folder holding Mantel csv files for appropriate cluster and replicate
 directory <- unique(my.df$folderpath[which(my.df$cluster==clusters[y] & my.df$replicate==z)])
 setwd(directory)

 # vector of landscapes associated with cluster y
 landscapes <- unique(my.df$landscape[which(my.df$cluster==clusters[y] & my.df$replicate==z)])

 # preallocate arrays to hold results for causal modeling (rows and columns are landscapes + Euclidean distance, third dimension is MC replicate)
 pval.array <- array(NA, dim=c(length(landscapes)+1, length(landscapes)+1, MCs)) # p-values
 mantelr.array <- array(NA, dim=c(length(landscapes)+1, length(landscapes)+1, MCs)) # mantel R values
 filenames <- list.files(getwd()) # get vector of file names for Mantel csv files

 for(i in 1:length(landscapes)) {
 for(j in 1:length(landscapes)) {
 for(k in 1:MCs) {
 if(i == j) {
 # fill in main diagonal of matrix with mantel tests of genetic ~ landscape (nothing partialed out)
 L1files <- list.files(pattern=paste("L1",landscapes[i], sep="")) # vector of files with correct L1
 testfiles <- list.files(pattern="gentoland.csv") # vector of files with correct mantel test
 selectfile <- L1files[L1files %in% testfiles] # file with correct L1 and mantel test
 selectfile <- selectfile[1] # if there are duplicates, use only the first file
 data <- read.csv(selectfile, header = FALSE, sep = ",")
 pvals <- data[,pvals.column]
 mantelrvals <- data[,mantelr.column]
 pval.array[i,j,k] <- pvals[k]
 mantelr.array[i,j,k] <- mantelrvals[k]

 } else {
 # fill in rest of matrix with partial mantel tests of genetic ~ landcape1 | landscape2 (for all possible combinations of landscapes)
 L1files <- list.files(pattern=paste("L1",landscapes[i], sep="")) # vector of files with correct L1
 L2files <- list.files(pattern=paste("L2",landscapes[j], sep="")) # vector of files with correct L2
 testfiles <- list.files(pattern="gentoland.land.csv") # vector of files with correct mantel test
 L1L2files <- L2files[L2files %in% L1files] # vector of files with correct L1 and L2
 selectfile <- L1L2files[L1L2files %in% testfiles] # file with correct L1, L2, and mantel test
 data <- read.csv(selectfile, header = FALSE, sep = ",")
 pvals <- data[,pvals.column]
 mantelrvals <- data[,mantelr.column]
 pval.array[i,j,k] <- pvals[k]
 mantelr.array[i,j,k] <- mantelrvals[k]
 }
 }
 }
 }

 # fill in last column (these are the partial mantel tests of genetic ~ landscape | distance)
 for(i in 1:length(landscapes)) {
 for(j in 1:MCs) {
 L1files <- list.files(pattern=paste("L1",landscapes[i], sep="")) # vector of files with correct L1
 testfiles <- list.files(pattern="gentoland.dist.csv")
 selectfile <- L1files[L1files %in% testfiles] # file with correct L1 and mantel test
 selectfile <- selectfile[1] # if there are duplicates, use only the first file
 data <- read.csv(selectfile, header = FALSE, sep = ",")
 pvals <- data[,pvals.column]
 mantelrvals <- data[,mantelr.column]
 pval.array[i,dim(pval.array)[2],j] <- pvals[j]
 mantelr.array[i,dim(mantelr.array)[2],j] <- mantelrvals[j]
 }
 }

 # fill in last row (these are the partial mantel tests of genetic ~ distance | landscape)
 for(i in 1:length(landscapes)) {
 for(j in 1:MCs) {
 L1files <- list.files(pattern=paste("L1",landscapes[i], sep="")) # vector of files with correct L1
 testfiles <- list.files(pattern="gentodist.land.csv")
 selectfile <- L1files[L1files %in% testfiles] # file with correct L1, L2, and mantel test
 selectfile <- selectfile[1] # if there are duplicates, use only the first file
 data <- read.csv(selectfile, header = FALSE, sep = ",")
 pvals <- data[,pvals.column]
 mantelrvals <- data[,mantelr.column]
 pval.array[dim(pval.array)[1],i,j] <- pvals[j]
 mantelr.array[dim(mantelr.array)[1],i,j] <- mantelrvals[j]
 }
 }

 # fill in the last element (this is the simple mantel test of genetic ~ distance)
 for(i in 1:MCs) {
 L1files <- list.files(pattern=paste("L1",landscapes[1], sep="")) # choose any L1 (doesn't matter)
 testfiles <- list.files(pattern="gentodist.csv")
 selectfile <- L1files[L1files %in% testfiles] # file with correct L1 and mantel test
 selectfile <- selectfile[1] # if there are duplicates, use only the first file
 data <- read.csv(selectfile, header = FALSE, sep = ",")
 pvals <- data[,pvals.column]
 mantelrvals <- data[,mantelr.column]
 pval.array[dim(pval.array)[1],dim(pval.array)[2],i] <- pvals[i]
 mantelr.array[dim(mantelr.array)[1],dim(mantelr.array)[2],i] <- mantelrvals[i]
 }

 ###############################################################################
 # matrix showing the # of consistent tests for each landscape/MC run combo
 consistent.mat <- matrix(NA, nrow=MCs, ncol=(length(landscapes)+1)) # preallocate matrix to hold # of consistent tests for each landscape/MC run combo
 for(i in 1:MCs) { # outer loop through each MC run (i.e., each set of genetic data)
 for(j in 1:(length(landscapes)+1)) { # loop through each landscape (including Eucl. distance)
 sig.elements <- as.numeric(as.vector(pval.array[j,-j,i]))
 nsig.elements <- as.numeric(as.vector(pval.array[-j,j,i]))
 consistent.mat[i,j] <- length(which(sig.elements<=alpha)) + length(which(nsig.elements>alpha))
 }
 }

 # matrix showing number of consistant tests if candidate set includes only the truth and the discriminant surface
 consistent.mat.just2 <- matrix(NA, nrow=MCs, ncol=2) # preallocate matrix to hold # of consistent tests for only truth and discriminant
 pval.array.just2 <- pval.array[c(truth.element, test.element), c(truth.element, test.element),] # subset pval.array to only rows/columns for truth and discriminant
 for(i in 1:MCs) { # outer loop through each MC run (i.e., each set of genetic data)
 for(j in 1:2) { # loop through each landscape (only truth and discriminant)
 sig.elements <- as.numeric(as.vector(pval.array.just2[j,-j,i]))
 nsig.elements <- as.numeric(as.vector(pval.array.just2[-j,j,i]))
 consistent.mat.just2[i,j] <- length(which(sig.elements<=alpha)) + length(which(nsig.elements>alpha))
 }
 }

 # matrix showing number of consistant tests if candidate set includes only the truth and the least similar surface based on same underlying variable ("leastsim")
 consistent.mat.leastsim <- matrix(NA, nrow=MCs, ncol=2) # preallocate matrix to hold # of consistent tests for only truth and leastsim
 pval.array.leastsim <- pval.array[c(truth.element, leastsim.element), c(truth.element, leastsim.element),] # subset pval.array to only rows/columns for truth and leastsim
 for(i in 1:MCs) { # outer loop through each MC run (i.e., each set of genetic data)
 for(j in 1:2) { # loop through each landscape (only truth and discriminant)
 sig.elements <- as.numeric(as.vector(pval.array.leastsim[j,-j,i]))
 nsig.elements <- as.numeric(as.vector(pval.array.leastsim[-j,j,i]))
 consistent.mat.leastsim[i,j] <- length(which(sig.elements<=alpha)) + length(which(nsig.elements>alpha))
 }
 }

 # vectors showing consistent runs for each landscape
 prop.perfect.p.vec <- rep(NA, (length(landscapes)+1)) # proportion of MC runs in which all possible tests were consistent with CM expection
 mean.consistent.vec <- rep(NA, (length(landscapes)+1)) # mean number of tests consistent with expectation (across MC runs)
 se.consistent.vec <- rep(NA, (length(landscapes)+1)) # standard error of # consistent tests
 require(plotrix)
 for(i in 1:(length(landscapes)+1)) {
 subtests <- as.numeric(as.vector(consistent.mat[,i]))
 prop.perfect.p.vec[i] <- length(which(subtests==(2*length(landscapes))))/MCs
 mean.consistent.vec[i] <- mean(subtests)
 se.consistent.vec[i] <- std.error(subtests)
 }
 # same as above, but for candidate set that only includes truth and discriminant
 prop.perfect.p.vec.just2 <- rep(NA, 2) # proportion of MC runs in which all possible tests were consistent with CM expection, for candidate set including only truth and discriminant
 for(i in 1:2) {
 subtests <- as.numeric(as.vector(consistent.mat.just2[,i]))
 prop.perfect.p.vec.just2[i] <- length(which(subtests==2))/MCs
 }
 # same as above, but for candidate set that only includes truth and leastsim
 prop.perfect.p.vec.leastsim <- rep(NA, 2) # proportion of MC runs in which all possible tests were consistent with CM expection, for candidate set including only truth and leastsim
 for(i in 1:2) {
 subtests <- as.numeric(as.vector(consistent.mat.leastsim[,i]))
 prop.perfect.p.vec.leastsim[i] <- length(which(subtests==2))/MCs
 }

 unequivocal.p.vec <- rep(0, MCs) # vector for proportion of MC runs for which the true surface has 10/10 consistent tests and all other surfaces have <10
 unequivocal.p.vec.test <- rep(0, MCs) # vector for proportion of MC runs for which the true surface has 10/10 consistent tests and the discriminant surface has <10
 unequivocal.p.vec.test.just2 <- rep(0, MCs) # vector for proportion of MC runs for which the true surface has 2/2 consistent tests and the discriminant surface has <2
 unequivocal.p.vec.leastsim.just2 <- rep(0, MCs) # vector for proportion of MC runs for which the true surface has 2/2 consistent tests and the leastsim surface has <2
 for(a in 1:MCs) {
 if(consistent.mat[a,truth.element]==10 & max(consistent.mat[a,-truth.element])<10) unequivocal.p.vec[a] <- 1
 if(consistent.mat[a,truth.element]==10 & consistent.mat[a,test.element]<10) unequivocal.p.vec.test[a] <- 1
 if(consistent.mat.just2[a,1]==2 & consistent.mat.just2[a,2]<2) unequivocal.p.vec.test.just2[a] <- 1
 if(consistent.mat.leastsim[a,1]==2 & consistent.mat.leastsim[a,2]<2) unequivocal.p.vec.leastsim.just2[a] <- 1
 }
 success.df$unequivocal.p[which(success.df$cluster==clusters[y] & success.df$replicate==z)] <- mean(unequivocal.p.vec)
 success.df$unequivocal.p.test[which(success.df$cluster==clusters[y] & success.df$replicate==z)] <- mean(unequivocal.p.vec.test)
 success.df$unequivocal.p.test.just2[which(success.df$cluster==clusters[y] & success.df$replicate==z)] <- mean(unequivocal.p.vec.test.just2)
 success.df$unequivocal.p.leastsim.just2[which(success.df$cluster==clusters[y] & success.df$replicate==z)] <- mean(unequivocal.p.vec.leastsim.just2)

 # calculate difference in Mantel r values for complementary partial mantels tests
 RS.array <- array(NA, dim=c(length(landscapes)+1, length(landscapes)+1, MCs)) # preallocate array of relative Mantel r
 # fill in each array element with difference between avg mantel r values from tests of 1) row surface | column surface and 2) column surface | row surface
 for(i in 1:dim(RS.array)[1]){
 for(j in 1:dim(RS.array)[2]){
 for(k in 1:dim(RS.array)[3]){
 RS.array[i,j,k] <- mantelr.array[i,j,k] - mantelr.array[j,i,k]
 }
 }
 }
 RS.array.just2 <- RS.array[c(truth.element,test.element), c(truth.element,test.element),] # pull out RS values for just the truth and discriminant
 RS.array.leastsim <- RS.array[c(truth.element,leastsim.element), c(truth.element,leastsim.element),] # pull out RS values for just the truth and leastsim

 avg.RS.mat <- matrix(NA, nrow=MCs, ncol=length(landscapes)+1) # preallocate matrix showing mean r diff for each landscape/MC run combo
 prop.RS.mat <- matrix(NA, nrow=MCs, ncol=length(landscapes)+1) # preallocate matrix showing proportion of r diffs that are positive for each landscape/MC run combo
 simple.r.mat <- matrix(NA, nrow=MCs, ncol=length(landscapes)+1) # preallocate matrix showing simple Mantel r for each landscape/MC run combo
 for(l in 1:nrow(avg.RS.mat)){
 for(m in 1:ncol(avg.RS.mat)){
 avg.RS.mat[l,m] <- mean(RS.array[m,-m,l])
 prop.RS.mat[l,m] <- length(which(RS.array[m,-m,l] > 0)) / length(landscapes)
 simple.r.mat[l,m] <- mantelr.array[m,m,l]
 }
 }
 avg.RS.mat.just2 <- matrix(NA, nrow=MCs, ncol=2) # preallocate matrix showing mean r diff for truth and discriminant
 for(l in 1:nrow(avg.RS.mat.just2)){
 for(m in 1:ncol(avg.RS.mat.just2)){
 avg.RS.mat.just2[l,m] <- mean(RS.array.just2[m,-m,l])
 }
 }
 avg.RS.mat.leastsim <- matrix(NA, nrow=MCs, ncol=2) # preallocate matrix showing mean r diff for truth and leastsim
 for(l in 1:nrow(avg.RS.mat.leastsim)){
 for(m in 1:ncol(avg.RS.mat.leastsim)){
 avg.RS.mat.leastsim[l,m] <- mean(RS.array.leastsim[m,-m,l])
 }
 }

 avg.RS.vec <- rep(NA, (length(landscapes)+1)) # vector for mean of the mean r diffs from the MC runs
 prop.perfect.r.vec <- rep(NA, (length(landscapes)+1)) # vector for proportion of the MC runs for which all R diffs were positive for that surface
 mean.simple.r.vec <- rep(NA, (length(landscapes)+1)) # vector for mean of simple mantel r from the MC runs
 for(n in 1:length(avg.RS.vec)){
 avg.RS.vec[n] <- mean(avg.RS.mat[,n])
 prop.perfect.r.vec[n] <- length(which(prop.RS.mat[,n]==1))/MCs
 mean.simple.r.vec[n] <- mean(simple.r.mat[,n])
 }

 unequivocal.RS.vec <- rep(0, MCs) # vector for proportion of MC runs for which the true surface has higher avg R diff than any of the alternative landscapes (i.e., unequivocal success at identifying true surface)
 unequivocal.RS.vec.test <- rep(0, MCs) # vector for proportion of MC runs for which the true surface has higher avg R diff than the discriminant landscape
 unequivocal.RS.vec.test.just2 <- rep(0, MCs) # vector for proportion of MC runs for which the true surface has higher R diff than the discriminant landscape (with no other surfaces in candidate set)
 unequivocal.RS.vec.leastsim.just2 <- rep(0, MCs) # vector for proportion of MC runs for which the true surface has higher R diff than the leastsim landscape (with no other surfaces in candidate set)
 for(v in 1:MCs) {
 if(avg.RS.mat[v,truth.element]==max(avg.RS.mat[v,])) unequivocal.RS.vec[v] <- 1
 if(avg.RS.mat[v,truth.element]==max(avg.RS.mat[v,c(truth.element, test.element)])) unequivocal.RS.vec.test[v] <- 1
 if(avg.RS.mat.just2[v,1]==max(avg.RS.mat.just2[v,])) unequivocal.RS.vec.test.just2[v] <- 1
 if(avg.RS.mat.leastsim[v,1]==max(avg.RS.mat.leastsim[v,])) unequivocal.RS.vec.leastsim.just2[v] <- 1
 }
 success.df$unequivocal.RS[which(success.df$cluster==clusters[y] & success.df$replicate==z)] <- mean(unequivocal.RS.vec)
 success.df$unequivocal.RS.test[which(success.df$cluster==clusters[y] & success.df$replicate==z)] <- mean(unequivocal.RS.vec.test)
 success.df$unequivocal.RS.test.just2[which(success.df$cluster==clusters[y] & success.df$replicate==z)] <- mean(unequivocal.RS.vec.test.just2)
 success.df$unequivocal.RS.leastsim.just2[which(success.df$cluster==clusters[y] & success.df$replicate==z)] <- mean(unequivocal.RS.vec.leastsim.just2)

 unequivocal.r.simple.vec <- rep(0, MCs) # vector for proportion of MC runs for which the true surface has higher mantel r from simple mantel test than any of the alternative landscapes (i.e., unequivocal success at identifying true surface)
 unequivocal.r.simple.vec.test <- rep(0, MCs) # vector for proportion of MC runs for which the true surface has higher mantel r from simple mantel test than the test (discriminant) surface
 unequivocal.r.simple.vec.leastsim <- rep(0, MCs) # vector for proportion of MC runs for which the true surface has higher mantel r from simple mantel test than the leastsim surface
 for(p in 1:MCs) {
 if(simple.r.mat[p,truth.element]==max(simple.r.mat[p,])) unequivocal.r.simple.vec[p] <- 1
 if(simple.r.mat[p,truth.element]==max(simple.r.mat[p,c(truth.element, test.element)])) unequivocal.r.simple.vec.test[p] <- 1
 if(simple.r.mat[p,truth.element]==max(simple.r.mat[p,c(truth.element, leastsim.element)])) unequivocal.r.simple.vec.leastsim[p] <- 1
 }
 success.df$unequivocal.r.simple[which(success.df$cluster==clusters[y] & success.df$replicate==z)] <- mean(unequivocal.r.simple.vec)
 success.df$unequivocal.r.simple.test[which(success.df$cluster==clusters[y] & success.df$replicate==z)] <- mean(unequivocal.r.simple.vec.test)
 success.df$unequivocal.r.simple.leastsim[which(success.df$cluster==clusters[y] & success.df$replicate==z)] <- mean(unequivocal.r.simple.vec.leastsim)

 my.df$mean.consistent[which(my.df$cluster==clusters[y] & my.df$replicate==z)] <- mean.consistent.vec[-length(mean.consistent.vec)]
 my.df$prop.perfect.p[which(my.df$cluster==clusters[y] & my.df$replicate==z)] <- prop.perfect.p.vec[-length(prop.perfect.p.vec)]
 my.df$mean.RS[which(my.df$cluster==clusters[y] & my.df$replicate==z)] <- avg.RS.vec[-length(avg.RS.vec)]
 my.df$prop.perfect.r[which(my.df$cluster==clusters[y] & my.df$replicate==z)] <- prop.perfect.r.vec[-length(prop.perfect.r.vec)]
 my.df$mean.simple.r[which(my.df$cluster==clusters[y] & my.df$replicate==z)] <- mean.simple.r.vec[-length(mean.simple.r.vec)]

 } # close loop Z (landscape replicate)
} # close loop y (cluster)

Final Results Tables

my.df[,-4] # raw performance metrics
success.df # unequiv
